# Metasurface‐Based Multidimensional Light‐Field Manipulation: From Technologies to Applications

**DOI:** 10.1002/advs.78390

**Published:** 2026-10-28

**Authors:** Heliang Yin, Shuo Sun, Jiahao Zhang, Jianuo Hu, Yubin Qiao, Liang Chen, Jin Li

**Affiliations:** ^1^ College of Optical and Electronic Technology China Jiliang University Hangzhou China; ^2^ School of Instrumentation and Optoelectronic Engineering Beihang University Beijing China

**Keywords:** metasurface, multidimensional light‐field manipulation, optical information processing, optical multiplexing, structured light, wavefront engineering

## Abstract

Metasurfaces composed of subwavelength meta‐atoms provide an ultrathin and highly integrable platform for manipulating optical wavefronts and have reshaped the development of flat optics and meta‐optics. Beyond conventional single‐parameter phase engineering, recent advances have enabled coordinated control of multiple degrees of freedom of light, including phase, amplitude, polarization, wavelength, spatiotemporal mode, and orbital angular momentum (OAM). This review summarizes the physical principles, implementation strategies, and representative applications of metasurface‐based multidimensional light‐field manipulation. We first discuss the fundamental mechanisms of metasurface‐based light‐field manipulation, including generalized Snell's law, phase discontinuity, meta‐atom scattering, and the modulation of basic optical degrees of freedom. We then review major multidimensional design strategies, such as joint phase‐amplitude, phase‐polarization, phase‐wavelength, phase‐OAM, spatial‐polarization, temporal‐phase, and higher‐dimensional control. Representative applications in optical information processing, computational imaging, holographic and near‐eye display, optical communication, structured‐light generation, and optical manipulation are further discussed. Finally, we highlight current challenges associated with efficiency, bandwidth, crosstalk, dynamic reconfigurability, fabrication tolerance, scalable manufacturing, and system‐level integration, and outline future opportunities in metasurface‐based multidimensional light‐field manipulation.

## Graphical abstract

Multidimensional metasurfaces enable versatile manipulation of multiple optical degrees of freedom, including phase, amplitude, polarization, wavelength, and orbital angular momentum. This review summarizes their fundamental mechanisms, emerging strategies for multidimensional light‐field control, and key applications in optical computing, imaging, communication, and structured‐light manipulation, while highlighting future opportunities toward high‐dimensional, integrated, and intelligent meta‐optics.

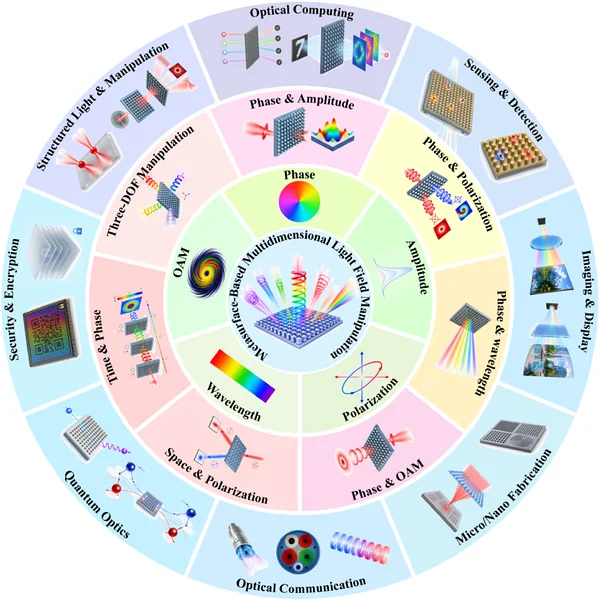

## 1 Introduction

Light is one of the most important carriers of information and energy in nature and underpins optical communication, optical imaging, precision metrology, information processing, and quantum technologies. Precise manipulation of light fields has therefore remained a central question in optics and photonics. A light field is not defined by intensity alone; it also encompasses multiple degrees of freedom, including amplitude, phase, polarization, wavelength, spatiotemporal mode, and orbital angular momentum (OAM) [1, 2]. These degrees of freedom jointly determine light propagation, focusing, diffraction, imaging, and information‐carrying capacity. Coordinated control of multiple optical degrees of freedom can substantially increase the encoding dimensionality and transmission capacity of optical information, thereby supporting high‐capacity optical communication [3], multifunctional optical devices [4], optical computing [5], and emerging display technologies [6]. With optical systems evolving toward greater integration, multifunctionality, and intelligence, multidimensional light‐field manipulation has emerged as an important frontier in modern optics.

Conventional optical systems usually rely on bulk optical components, such as lenses, prisms, wave plates, and polarizers, as well as programmable devices such as spatial light modulators. These components tailor wavefront, polarization, and intensity distributions through propagation‐phase accumulation or cascaded multi‐element assemblies. Although such approaches have been widely used in imaging, measurement, and communication, their bulky form factors, structural complexity, and limited integration density impose clear constraints on miniaturized and integrated optical systems [7, 8]. In scenarios requiring simultaneous control of multiple optical degrees of freedom, conventional systems often require cascaded discrete components, increasing the system footprint and alignment complexity [9], while potentially introducing additional loss, aberrations, and inter‐channel crosstalk. Even planar diffractive optical elements can be limited by diffraction efficiency, chromatic dispersion, and insufficient control dimensionality [10, 11]. Therefore, developing ultrathin, compact, and integrable optical platforms capable of jointly controlling multiple degrees of freedom of light has become an important route to overcoming the limitations of conventional optical systems.

The emergence of metasurfaces has provided a new solution for light‐field manipulation. A metasurface is typically composed of artificial nanostructural elements at the subwavelength scale, commonly referred to as meta‐atoms [12]. By rationally designing their geometry, material composition, spatial arrangement, and orientation angle, metasurfaces can locally and controllably modulate the phase, amplitude, and polarization of incident light on an ultrathin two‐dimensional interface [13]. Unlike conventional optical elements that rely primarily on phase accumulation along an optical path, metasurfaces introduce phase discontinuities at an interface, enabling precise wavefront engineering. According to generalized Snell's law, a spatial phase gradient at an interface imparts additional transverse momentum to incident light, thereby enabling anomalous reflection and refraction that deviate from the predictions of the conventional laws of reflection and refraction [14]. This principle helped establish the field of flat optics and was subsequently applied to beam steering, metalenses, holographic imaging, polarization modulation, and structured‐light generation [15].

Early metasurface studies focused primarily on wavefront manipulation through spatial phase engineering. By arranging meta‐atoms with different phase responses on a two‐dimensional plane, dielectric metasurfaces can control optical wavefronts at the subwavelength scale and realize beam steering, wavefront shaping, ultrathin lenses, vortex‐beam generation, and holographic imaging [16]. With advances in nanofabrication, material platforms, and design methodologies, the functionality of metasurfaces has expanded from phase‐only modulation to the coordinated manipulation of multiple optical degrees of freedom. In this review, metasurface‐based multidimensional light‐field manipulation refers to the joint design, multiplexing, and control of phase, amplitude, polarization, wavelength, spatiotemporal mode, OAM, and related optical degrees of freedom within a single metasurface or an integrated metasurface platform.

Compared with single‐degree‐of‐freedom control, multidimensional manipulation allows different optical degrees of freedom to serve as independent or coupled information channels, thereby increasing control dimensionality, information capacity, and device functional density. Joint phase‐amplitude control enables complex‐amplitude encoding and improves holographic reconstruction and complex beam shaping. Combining geometric and propagation phases enables polarization‐selective wavefront control and polarization multiplexing. Dispersion engineering enables metasurfaces to exhibit distinct optical responses at different wavelengths, supporting multicolor displays, achromatic imaging, and spectral multiplexing. Joint phase‐OAM control provides new routes for vortex optical communication, high‐dimensional optical information encoding, and capacity expansion. Metasurfaces are therefore evolving from single‐function wavefront shapers into multichannel, multifunctional, and highly integrated light‐field manipulation platforms.

Despite substantial progress, metasurface‐based multidimensional light‐field manipulation still faces key challenges, including efficiency enhancement and broadband operation [17], as well as multifunctional integration and dynamic reconfigurability [18, 19]. When several degrees of freedom are jointly manipulated within the same metasurface, complex coupling between channels can lead to crosstalk, efficiency degradation, and control errors, limiting performance in holographic displays, spectral multiplexing, and information demultiplexing [20, 21]. Multifunctional integration also entails trade‐offs among efficiency, bandwidth, numerical aperture (NA), field of view, and structural complexity. For example, broadband achromatic metalenses must simultaneously satisfy phase and group‐delay matching at different wavelengths, and are therefore constrained by trade‐offs among bandwidth, numerical aperture, device aperture, and focusing efficiency [22]. In addition, many multidimensional metasurfaces rely on complex nanostructures and large‐scale optimization algorithms, placing stringent demands on computational resources, fabrication precision, and process stability during design, fabrication, and characterization. Dynamic tunability, environmental stability, fabrication tolerance, and compatibility with existing optoelectronic systems also remain central barriers to practical deployment [23, 24]. Addressing these challenges will be essential for developing efficient, low‐crosstalk, broadband, reconfigurable, and manufacturable multidimensional metasurfaces.

Several representative reviews have addressed metasurface‐based multidimensional optical‐field manipulation from complementary perspectives, including the evolution from single‐dimensional to multidimensional control [25], optical multiplexing and multifunctional metasurfaces [26], design strategies and applications of dimensional optical‐field manipulation [27], and optical‐field information manipulation based on nonlocal metasurfaces from passive to active systems [28]. Taken together, these studies provide complementary perspectives on dimensional evolution, multiplexing and multifunctionality, design strategies and optical dimensions, and nonlocal information manipulation, respectively. Building on these perspectives, the present review is positioned along three related dimensions. In terms of scope, it considers phase, amplitude, polarization, wavelength, OAM, space, and time as key degrees of freedom for multidimensional light‐field manipulation. In terms of organization, it adopts a mechanism‐to‐strategy‐to‐application framework that connects fundamental physical mechanisms with multidimensional control strategies and representative applications. From a scientific perspective, these optical degrees of freedom are treated as designable, encodable, coupled, and multiplexable information channels for multidimensional light‐field control.

Accordingly, the overall scope and conceptual framework of this review are schematically summarized in Figure 1. We first introduce the fundamental principles of metasurfaces and the underlying mechanisms of light‐field manipulation, including generalized Snell's law, phase discontinuities, the scattering mechanisms of meta‐atoms, and approaches for controlling fundamental optical degrees of freedom such as phase, amplitude, polarization, wavelength, and OAM. We then review the major strategies for multidimensional manipulation, including joint phase‐amplitude, phase‐polarization, phase‐wavelength, phase‐OAM, spatial‐polarization, temporal‐phase, and joint control of multiple degrees of freedom. Representative applications span optical information processing and computing, imaging and displays, optical communication and information transmission, structured optical‐field manipulation and optical manipulation, optical micro‐ and nanofabrication, sensing and detection, quantum optics and quantum information, as well as optical security, anti‐counterfeiting, and encryption. Among these areas, this review focuses primarily on optical information processing and computing, imaging and displays, optical communication and information transmission, and structured optical‐field manipulation and optical manipulation. Finally, we summarize the key challenges facing multidimensional metasurfaces and discuss future opportunities in high‐dimensional optical information control, integrated photonic systems, and intelligent meta‐optical devices.

**FIGURE 1:**
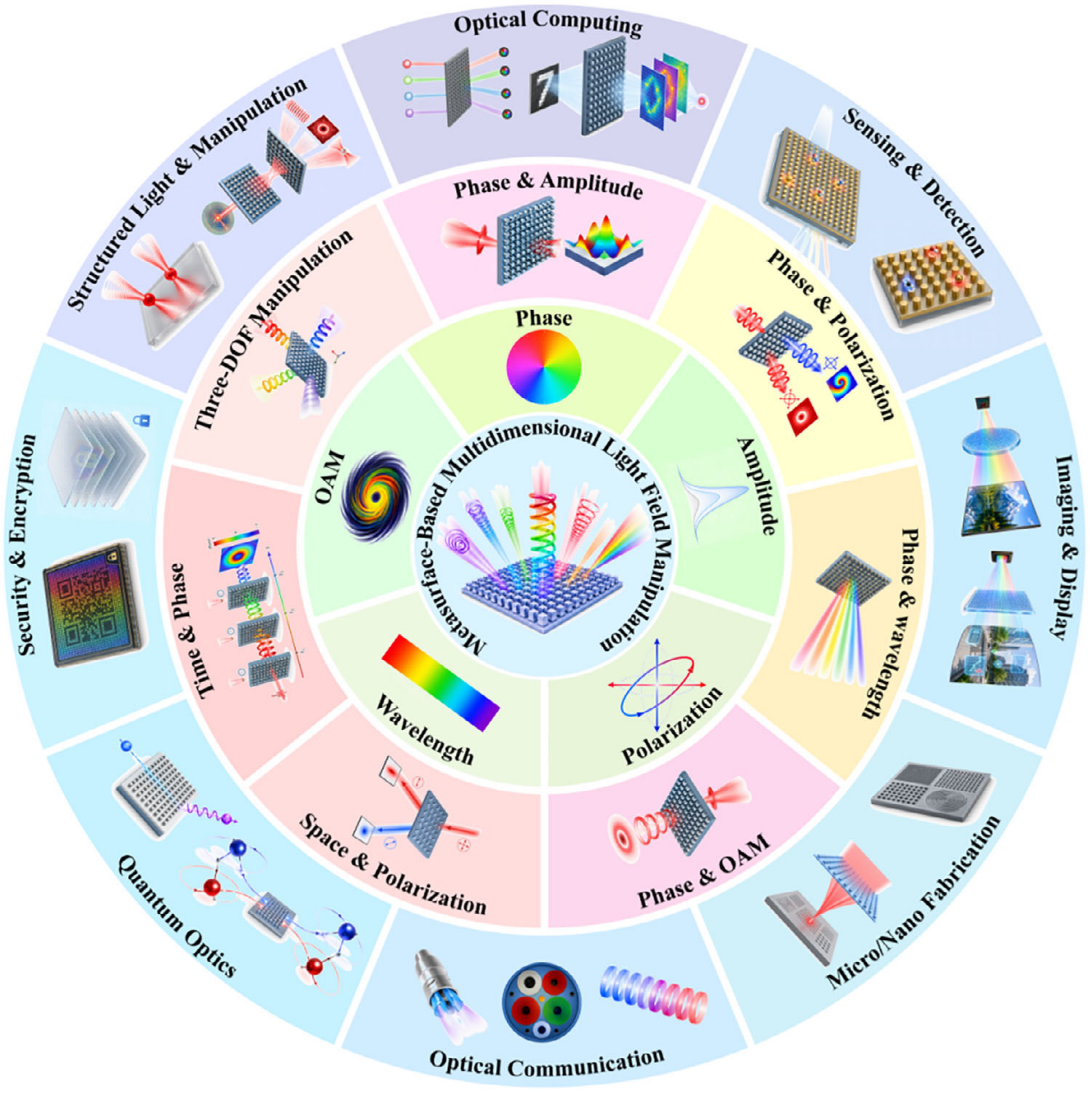
Schematic overview of metasurface‐based multidimensional light‐field manipulation by metasurfaces. The illustration was made using Blender (version 4.0.2).

## 2 Fundamental Principles and Light‐Field Manipulation Mechanisms

### 2.1 Fundamentals of Metasurfaces

#### 2.1.1 Generalized Snell's Law

The conventional form of Snell's law describes the refraction of electromagnetic waves at an interface between two homogeneous media, with the propagation direction of the refracted wave determined by the angle of incidence and the refractive indices of the two media. Because this law assumes that no additional phase modulation is introduced at the interface, it offers limited flexibility in controlling the propagation direction of electromagnetic waves.

In 2011, Capasso et al. proposed generalized Snell's law to overcome this limitation and achieved control over the propagation directions of reflected and refracted waves by introducing a spatially varying phase discontinuity at an interface [14], as illustrated in Figure 2a. When light is incident on the interface between two media at an angle θ_*i*_, and the metasurface imposes a phase profile Φ(*x*) along the interfacial direction *x*, the refracted wave satisfies:

(1) $$\begin{array}{c} n_{t}\sin\theta_{t}\;-\;n_{i}\;\sin\theta_{i}=\frac{\lambda_{0}}{2\pi }\;\frac{d\Phi \left(x\right)}{dx} \end{array}$$

here, *n_i_* and *n_t_* are the refractive indices of the incident and transmitted media, respectively; θ_*i*_ and θ_*t*_ denote the angles of incidence and refraction, respectively; λ_0_ is the vacuum wavelength; and *d*Φ(*x*)/*dx* represents the phase gradient introduced by the metasurface along the interface. Compared with conventional Snell's law, generalized Snell's law contains an additional term arising from the transverse momentum imparted by the interfacial phase gradient. Consequently, the propagation direction of the refracted wave depends not only on the refractive indices of the two media and the angle of incidence, but also on the designed phase gradient of the metasurface. For the reflection process, the generalized law of reflection can be expressed as:

(2) $$\begin{array}{c} \sin\theta_{r}-\sin\theta_{i}=\frac{\lambda_{0}}{2\pi n_{i}}\;\frac{d\Phi \left(x\right)}{dx} \end{array}$$

where θ_*r*_ denotes the angle of reflection. This relation indicates that, in the presence of a nonzero interfacial phase gradient, the angle of reflection no longer obeys the conventional specular‐reflection condition θ_*r*_  =  θ_*i*_, resulting in anomalous reflection. In 2013, Sun et al. further derived the generalized laws of reflection and refraction from Fermat's principle and electromagnetic boundary conditions. They showed that when *d*Φ(*x*)/*dx*  =  0, the generalized laws reduce to the conventional law of reflection and Snell's law, respectively [29]. Their analysis clarified the effects of the phase gradient, angle of incidence, and refractive indices of the media on anomalous reflection and refraction, providing further theoretical insight into light propagation at phase‐discontinuous interfaces.

**FIGURE 2:**
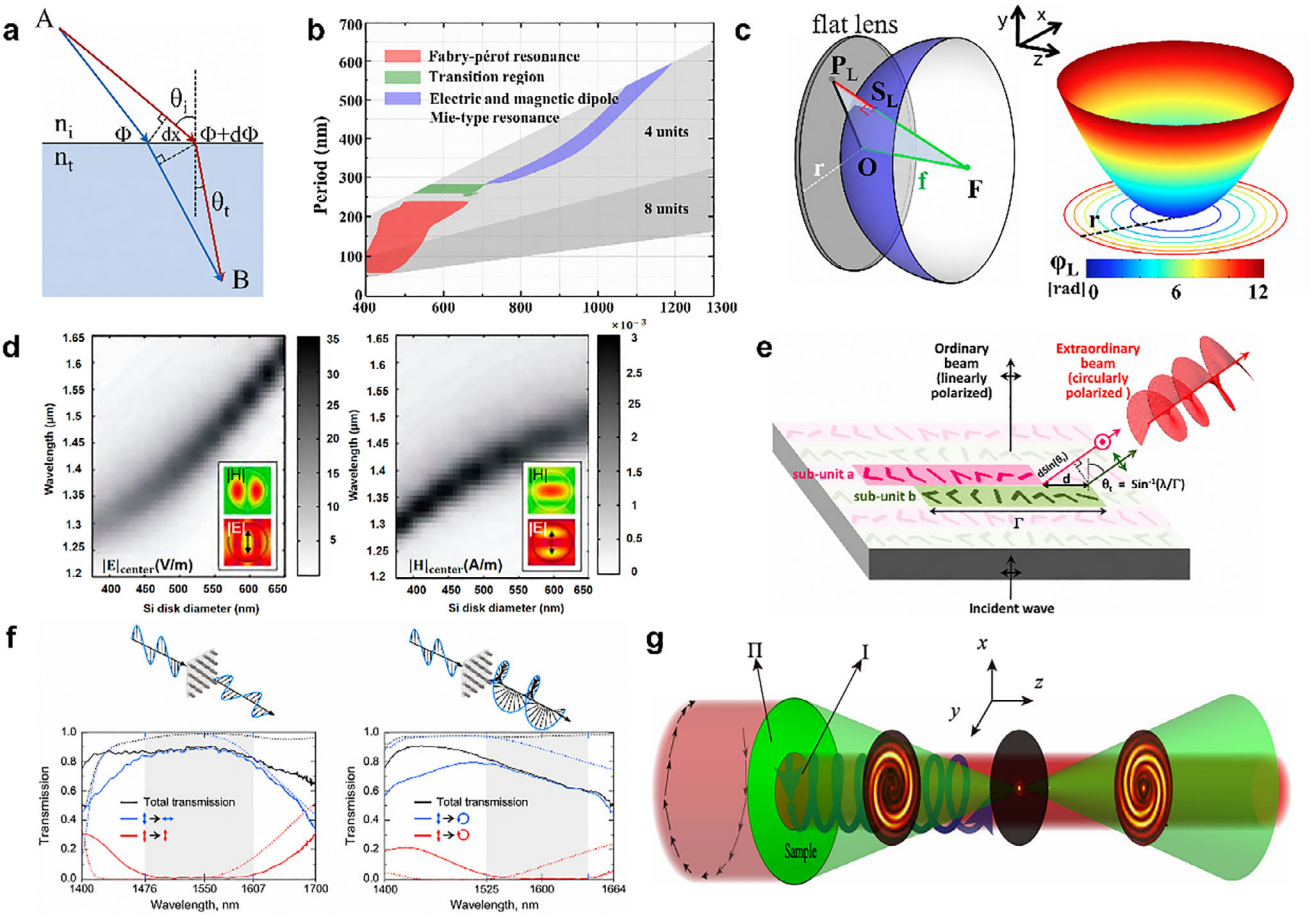
Fundamental physical mechanisms and representative functions of metasurface‐based multidimensional light‐field manipulation. (a) Physical model of generalized Snell's law at a phase‐discontinuous interface. Reprinted with permission [14]. Copyright 2011, The American Association for the Advancement of Science. (b) Main physical regimes for achieving 2*π* phase modulation in dielectric metasurfaces. Reprinted with permission [40]. Copyright 2019, Optical Society of America. (c) Flat lens and its hyperboloidal radial phase distribution. Reprinted with permission [41]. Copyright 2012, American Chemical Society. (d) Electric and magnetic dipole resonances in silicon nanodisks. Reprinted with permission [43]. Copyright 2013, American Chemical Society. (e) Background‐suppressed quarter‐wave plate based on a plasmonic metasurface. Reprinted with permission [64]. Copyright 2012, American Chemical Society. (f) Broadband polarization conversion of all‐dielectric half‐ and quarter‐wave plates. Reprinted under the terms of the CC BY 4.0 license [68]. Copyright 2016, The Authors. (g) orbital angular momentum (OAM) generation and detection using an elliptical nanohole metasurface. Reprinted under the terms of the CC BY 4.0 license [79]. Copyright 2016, The Authors.

From the perspective of momentum matching, a metasurface can be regarded as a two‐dimensional engineered interface whose spatial phase gradient imparts an additional in‐plane wave‐vector component, thereby modifying the tangential wave‐vector matching condition while conserving energy. For the transmission process, in which light passes through the metasurface and subsequently propagates in the transmitted medium, the tangential wave‐vector matching condition can be expressed as [14, 30]:

(3) $$\begin{array}{c} \;k_{t,∥}=k_{i,∥}+\nabla_{∥}\Phi \end{array}$$

where *k*_*i*,∥_ and *k*_*t*,∥_ denote the tangential wave‐vector components of the incident and transmitted waves, respectively, and ∇_∥_Φ represents the in‐plane phase gradient imparted by the metasurface. By engineering the local phase profile, metasurfaces enable control over the propagation directions of reflected and refracted waves with a device thickness well below the operating wavelength.

Generalized Snell's law provides the basic theoretical framework for metasurface wavefront engineering. A linear phase gradient enables anomalous refraction and reflection, whereas more complex spatially varying phase profiles enable focusing, beam shaping, vortex‐beam generation, and holographic reconstruction. In 2012, Belardini et al. used curved gold nanowire metasurfaces to investigate asymmetric transmission and anomalous refraction, confirming that resonant metallic nanostructures can introduce pronounced phase discontinuities at an interface [31]. In practice, however, continuous phase profiles are approximated by discrete meta‐atom arrays. Finite phase quantization, lattice spacing, local impedance mismatch, near‐field coupling, and undesired diffraction channels can all reduce efficiency. Recent work has further shown that the conventional Nyquist sampling criterion alone is insufficient to fully determine aliasing‐free operation in metasurfaces, because the occurrence of unintended diffraction also depends on the geometric relationship between the spectrum morphology and the sampling lattice. In 2025, Kim et al. developed a lattice‐based diffraction analysis and demonstrated anti‐aliasing strategies for high‐NA metasurfaces from the visible to the near‐ultraviolet regime [32]. More broadly, impedance‐matching strategies, nonlocal scattering control, and low‐loss dielectric meta‐atoms have also been explored to enable high‐efficiency wavefront manipulation [33, 34, 35].

#### 2.1.2 Phase Discontinuity and Wavefront Engineering

Within the framework of generalized Snell's law, an interfacial phase gradient imparts additional tangential momentum to incident waves, enabling flexible control over reflected and transmitted beams. Unlike conventional optical components, which rely on phase accumulation over a finite propagation distance, metasurfaces introduce local phase discontinuities through subwavelength meta‐atoms, thereby imposing prescribed spatial phase distributions within an ultrathin layer. Achieving full 0–2π phase coverage with subwavelength spatial sampling is therefore a key requirement for metasurface wavefront engineering.

These local phase discontinuities are implemented using subwavelength artificial building blocks, commonly referred to as meta‐atoms. By tailoring their geometrical dimensions, shapes, and orientations, prescribed phase shifts can be imparted at an interface. The principal mechanisms used to generate these local phase responses include propagation, geometric, and resonant phases.

The propagation‐phase results from phase accumulation as electromagnetic waves propagate through a meta‐atom and is determined by the effective optical path length, which can be expressed as:

(4) $$\Phi =k_{0}\;n_{\mathrm{eff}}L$$

where Φ is the accumulated propagation phase, *k*_0_ is the free‐space wave number, *n*_eff_ is the effective refractive index of the structure, and *L* is the propagation length. By varying the geometrical dimensions or thickness of the meta‐atoms, the effective optical path length can be adjusted, thereby enabling continuous control over the transmission phase. In 2016, Zhan et al. demonstrated low‐index‐contrast dielectric metasurface lenses and vortex‐beam generators in the visible regime using cylindrical silicon nitride meta‐atoms, illustrating geometry‐dependent phase modulation in a low‐index‐contrast dielectric platform [36].

The Pancharatnam–Berry (PB) phase is generally introduced by controlling the orientations of anisotropic meta‐atoms, with the phase response determined by the local orientation angle. In 2015, Wang et al. designed a dual‐polarity plasmonic metalens based on optical‐axis rotation, for which incident circularly polarized light with opposite handedness experienced focusing and divergence, respectively [37]. In 2024, Gan et al. extended this concept by employing the generalized PB‐phase to realize broadband spin‐selective wavefront manipulation [38]. These studies illustrate how the orientation‐dependent phase response of meta‐atoms can couple the polarization state of light to the resulting wavefront transformation.

The resonant phase originates from localized or coupled electromagnetic resonances supported by the meta‐atoms and can be tailored by adjusting the structural dimensions, lattice period, material parameters, and mode‐coupling conditions. In 2015, Decker et al. demonstrated dielectric Huygens metasurfaces based on silicon nanodisks, in which spectrally overlapping electric‐ and magnetic‐dipole resonances enabled full transmission‐phase coverage with high transmission [39]. In 2019, Li et al. systematically investigated the mechanisms underlying complete 0–2*π* phase coverage in dielectric metasurfaces and identified distinct wavelength‐period regimes associated with Fabry–Pérot resonances, Mie‐type electric and magnetic dipole resonances, and the transition region between them, as illustrated in Figure 2b [40].

Spatially arranging these local phase responses across an interface translates microscopic phase modulation into macroscopic wavefront reconstruction. In 2012, Aieta et al. designed an ultrathin planar lens and an axicon based on arrays of plasmonic V‐shaped nanoantennas, using radial phase discontinuities to generate a focused spherical wavefront and an approximately non‐diffracting Bessel beam, respectively [41]. Among these devices, the planar lens shown in Figure 2c encodes a hyperboloidal radial phase profile across the metasurface, causing the incident plane wave to converge coherently at a prescribed focal plane. In 2015, Wang et al. further proposed nonuniform dielectric terahertz lenses in which the required radial phase distributions were constructed by varying the thickness of polyimide meta‐atoms, with full‐wave simulations confirming the focusing of transmitted terahertz waves [42].

Collectively, these studies highlight the direct link between local phase discontinuities and global wavefront transformations. Phase discontinuities provide the microscopic physical basis for generalized Snell's law at engineered interfaces, while their spatial encoding establishes a general framework for metasurface wavefront engineering.

#### 2.1.3 Scattering Mechanisms of Meta‐Atoms

The ability of a metasurface to manipulate light ultimately originates from scattering interactions between meta‐atoms and incident electromagnetic waves. Because meta‐atoms are subwavelength structures, their geometry, material refractive index, lattice period, and local resonances jointly determine phase, amplitude, and polarization responses. Understanding meta‐atom scattering at the microscopic level is therefore essential for explaining wavefront control.

In dielectric metasurfaces, Mie resonances are a major source of meta‐atom response. High‐index dielectric structures can support electric and magnetic dipole resonances that tailor the amplitude and phase of transmitted and reflected light. In 2013, Staude et al. studied electric and magnetic dipole resonances in subwavelength silicon nanodisks and showed that tuning the disk aspect ratio changes resonance positions and their interference, thereby suppressing backward scattering and enhancing forward scattering, as illustrated in Figure 2d [43]. Optimizing the three‐dimensional geometry of meta‐atoms further improves phase coverage and transmittance. In 2017, Donda and Hegde proposed double‐layer stepped conical silicon nanoantennas and showed that they can achieve full 0–2π phase coverage with higher transmission than conventional cylindrical elements in the 700–800 nm visible range [44].

Huygens meta‐atoms are particularly important for high‐efficiency metasurfaces. Their principle is to excite properly balanced electric and magnetic dipoles so that forward scattering is enhanced and backward scattering is suppressed. Under the Huygens condition, cooperative multipolar responses determine not only local phase modulation but also overall wavefront‐control efficiency. In 2022, Nguyen and Byun proposed a Huygens metasurface for wide‐angle refraction by independently controlling electric and magnetic dipole responses in line‐ring structures, experimentally directing more than 83% of the incident power toward the target direction at refraction angles up to 65° [45]. In the same year, Wang et al. designed angle‐stable Huygens meta‐atoms that maintained high transmission amplitude and small phase variation over 0°–60° incidence for holographic metasurfaces [46]. In 2023, Zhou et al. proposed bilayer Huygens meta‐atoms with cooperative electric and magnetic dipole responses for dual‐polarized metalens antennas, achieving near‐complete phase coverage and high transmission [47].

### 2.2 Basic Degrees of Freedom in Metasurface Light‐Field Manipulation

#### 2.2.1 Phase Modulation

Phase is a central physical quantity describing the wavefront distribution of an optical field and directly influences the propagation direction, focusing characteristics, and interference behavior of electromagnetic waves. For a monochromatic optical field, its complex amplitude *E*(*x*, *y*) can be expressed as:

(5) $$E\left(x,y\right)=A\left(x,y\right)\exp\left[i\Phi \left(x,y\right)\right]$$

where *A*(*x*, *y*) denotes the amplitude distribution, Φ(x, y) represents the spatial phase distribution, *x* and *y* are the transverse spatial coordinates, and *i* is the imaginary unit. By arranging meta‐atoms with different phase responses across a two‐dimensional interface, metasurfaces can construct prescribed local phase distributions at the subwavelength scale, thereby enabling control over transmitted or reflected wavefronts. Phase modulation therefore constitutes one of the most fundamental and widely used degrees of freedom in metasurface‐based light‐field manipulation.

Depending on the form of the spatial phase distribution, metasurface phase modulation can be broadly categorized into linear phase‐gradient modulation and nonlinear spatial phase modulation. A linear phase gradient is one of the most fundamental and representative forms of phase modulation. It imparts an additional tangential wave‐vector component to the incident light, thereby altering the propagation directions of the reflected or transmitted waves and enabling beam steering, anomalous reflection, and anomalous refraction [48]. Beyond linear phase gradients, more complex spatial phase profiles, including lens, axicon, helical, and holographic phase profiles, can be encoded into metasurfaces to realize focusing, collimation, Bessel‐beam generation, vortex‐beam generation, and holographic wavefront reconstruction [41, 49, 50, 51]. Thus, phase modulation not only redirects optical beams but also reconstructs their wavefronts according to prescribed phase functions, constituting one of the central mechanisms of metasurface wavefront engineering.

Early metasurface phase modulation relied predominantly on the plasmonic resonances of metallic nanoantennas, with different local phase delays obtained by tailoring their geometrical parameters and orientations. However, metallic structures are generally subject to Ohmic losses [52], while some early transmissive schemes relied on cross‐polarized scattering channels for phase encoding [14], thereby limiting the overall phase‐modulation efficiency. To improve the modulation efficiency, low‐loss dielectric metasurfaces gradually emerged as an important research direction. In 2014, Lin et al. reported dielectric gradient‐metasurface optical elements operating in the visible regime [53]. Using arrays of silicon nanobeams with a thickness of approximately 100 nm, they realized ultrathin gratings, lenses, and axicons, demonstrating the applicability of dielectric structures to transmissive visible‐light wavefront manipulation.

For high‐efficiency transmissive phase modulation, full 0–2π transmission‐phase coverage is a key prerequisite for synthesizing prescribed wavefronts. In 2015, Yu et al. reported a high‐transmission dielectric metasurface that achieved full 0–2π transmission‐phase coverage in the visible regime by varying the radii of silicon nanodisks, and further constructed a single‐layer gradient metasurface [54]. In the same year, Shalaev et al. reported an all‐dielectric metasurface based on silicon nanoblocks. By tailoring the geometrical dimensions of the nanoblocks, the metasurface achieved 0–2π transmission‐phase coverage in the near‐infrared regime and demonstrated beam steering and vortex‐beam generation [55]. These studies showed that tailoring the structural parameters and electric and magnetic resonances of high‐index dielectric meta‐atoms can provide full phase coverage and relatively high transmission efficiency while substantially reducing Ohmic losses, thereby establishing an important foundation for high‐efficiency transmissive metasurface phase modulation.

At the meta‐atom level, two representative strategies are widely employed to realize such local phase modulation: propagation‐phase modulation based on geometry‐dependent optical‐path accumulation and PB‐phase modulation based on the orientation of anisotropic meta‐atoms. In the propagation‐phase approach, as described by Equation (4), the accumulated phase is governed by the effective optical path within the meta‐atom. High‐aspect‐ratio dielectric nanopillars can be approximately regarded as truncated waveguides supporting confined optical modes. Their lateral dimensions, such as the radius or diameter, modify the modal confinement and consequently the effective refractive index *n*_eff_, whereas the height determines the propagation length. Therefore, by adjusting the geometric parameters of meta‐atoms, the required local phase responses can be selected from a pre‐designed 0–2π phase library. In 2016, Khorasaninejad et al. demonstrated polarization‐insensitive metalenses at visible wavelengths using TiO_2_ nanopillars, where varying the nanopillar diameter modified the effective refractive index of the propagating mode, and the waveguiding effect was identified as the dominant mechanism for phase realization [56]. This approach can provide full phase coverage together with high transmittance in low‐loss dielectric platforms, while rotationally symmetric meta‐atoms can additionally enable polarization‐insensitive operation. However, the phase response is dependent on wavelength and structural dimensions and is therefore sensitive to fabrication variations. Moreover, achieving complete 2π phase coverage generally requires sufficiently tall, high‐aspect‐ratio nanostructures, which increases fabrication difficulty, particularly at shorter visible wavelengths.

A conceptually different strategy is geometric‐phase modulation, commonly referred to as the PB‐phase. Instead of continuously varying the lateral dimensions of the meta‐atoms, this approach controls the local phase through the in‐plane orientation of anisotropic structures. For circularly polarized illumination, the cross‐circularly polarized component acquires a PB‐phase:

(6) $$\begin{array}{c} \Phi_{\mathrm{PB}}=2\sigma \theta \end{array}$$

where Φ_PB_ denotes the PB‐phase, σ =   ± 1 denotes the spin state of the incident circularly polarized light, and θ is the in‐plane orientation angle of the meta‐atom. Consequently, rotating otherwise identical anisotropic meta‐atoms over an angular range of 0–π provides complete 0–2π phase coverage. In the same year, Khorasaninejad et al. applied this PB‐phase principle to high‐NA TiO_2_ metalenses operating at visible wavelengths, in which spatially varying orientations of anisotropic nanofins encoded the required focusing phase [57]. Because the phase is mapped directly to the orientation angle, PB‐phase modulation offers a particularly simple phase‐encoding rule and reduces the need for a large library of meta‐atoms with different lateral dimensions. However, efficient operation requires the anisotropic meta‐atoms to provide high polarization‐conversion efficiency and to operate approximately as half‐wave plates. Imperfect retardation reduces the power transferred to the desired cross‐polarized component. In addition, the sign of the PB‐phase reverses with the incident helicity, making conventional PB‐phase devices intrinsically spin‐dependent. Although the PB‐phase itself is determined by the orientation angle, the polarization‐conversion efficiency and overall device response remain wavelength‐dependent, and chromatic aberration can therefore persist in practical devices.

Building on these phase‐control strategies, propagation and PB‐phases can be further combined with polarization‐selective responses to enable more flexible and multifunctional wavefront manipulation. In 2017, Mueller et al. combined propagation and PB‐phases to independently control the spatial phase profiles imparted to any pair of orthogonal polarization states, including linear, circular, and elliptical polarizations, and demonstrated chiral holograms and elliptical‐polarization beam splitters [20]. In 2018, Zhang et al. further designed a range of planar optical elements based on an amorphous TiO_2_ dielectric metasurface platform, including beam deflectors, polarization‐insensitive metalenses, wave plates, and polarization beam splitters [58]. These studies demonstrate that the coordinated control of phase response and anisotropic response can extend metasurface phase modulation from single‐channel wavefront shaping to polarization‐dependent and multifunctional light‐field manipulation.

#### 2.2.2 Amplitude Modulation

In Equation (5), the amplitude *A*(*x*, *y*) determines the intensity distribution and energy allocation of a light field. While phase modulation controls propagation, focusing, and interference, phase‐only modulation cannot independently prescribe the output intensity profile. Amplitude modulation controls the strength of transmitted or reflected light at different spatial positions and is therefore another essential degree of freedom.

Metasurface amplitude modulation can be implemented through absorption‐based modulation, scattering‐channel engineering, and polarization‐dependent response control. In absorption‐based schemes, the resonance characteristics, impedance‐matching conditions, or material losses are tailored to selectively suppress the transmitted or reflected intensity within specific spectral ranges. In 2014, Yao et al. integrated a graphene‐loaded optical‐antenna metasurface into a subwavelength optical cavity to realize an electrically tunable perfect absorber, in which gate‐voltage control of the graphene conductivity enabled near‐complete modulation of mid‐infrared absorption [59]. In 2024, Zhang et al. proposed a multifunctional graphene/VO_2_ terahertz metamaterial device [60]. The phase state of VO_2_ enabled switching between broadband absorption and high‐transmission modes, while tuning the graphene Fermi level and VO_2_ conductivity provided further control over the absorption bandwidth and response amplitude. These studies demonstrate dynamically reconfigurable amplitude modulation based on critical‐coupling control and active‐material tuning. However, absorption‐based modulation inherently dissipates a portion of the incident electromagnetic energy, which may limit its suitability for applications requiring high power efficiency.

Amplitude can also be controlled by tailoring scattering channels and polarization‐dependent responses. In 2016, Jia et al. proposed an ultrathin broadband reflective metasurface based on I‐shaped metallic elements, in which the geometrical parameters and orientation angles enabled independent control over the amplitude and phase of the cross‐polarized reflected wave [61]. In 2020, Dai et al. used Malus‐law‐assisted metasurfaces to convert orientation‐dependent anisotropy into grayscale intensity patterns and to encode two independent binary grayscale image channels [62]. In the same year, Fan et al. used birefringent dielectric meta‐atoms and polarization‐dependent interference to independently control two amplitude distributions in arbitrary orthogonal polarization bases, enabling chiral grayscale images and structured‐light shadow rendering [63]. In practice, amplitude modulation is often combined with phase and polarization to achieve high‐fidelity complex‐amplitude modulation.

#### 2.2.3 Polarization Modulation

Polarization describes the oscillation direction and temporal trajectory of the electric‐field vector and is a key degree of freedom of optical fields. Metasurfaces can convert between linear, circular, and elliptical polarizations, and can assign distinct transmission, reflection, phase, or amplitude responses to different polarization channels. Polarization modulation therefore underpins polarization multiplexing, polarization imaging, spin‐selective control, and multichannel information transmission. In general, polarization manipulation by a metasurface can be described using a Jones matrix:

(7) $$\left(\begin{array}{c} E_{x}^{\prime }\\ E_{y}^{\prime } \end{array}\right)=\left(\begin{array}{cc} t_{xx} & t_{xy}\\ t_{yx} & t_{yy} \end{array}\right)\;\left(\begin{array}{c} E_{x}\\ E_{y} \end{array}\right)$$

where *E_x_* and *E_y_* denote the orthogonal polarization components of the incident field, while $E_{X}^{\prime }$ and $E_{y}^{\prime }$ represent the corresponding polarization components of the output field. The diagonal elements *t_xx_* and *t_yy_* are the co‐polarized transmission coefficients, whereas the off‐diagonal elements *t_xy_* and *t_yx_* describe cross‐polarization conversion. By tailoring the amplitude and phase responses of these matrix elements, metasurfaces can realize diverse polarization transformations and polarization‐channel control.

Metasurface polarization modulation is fundamentally enabled by the anisotropic phase retardation of meta‐atoms. Because anisotropic meta‐atoms exhibit different effective refractive indices and electromagnetic responses along two orthogonal axes, they can impart distinct phase delays and amplitude responses to the corresponding orthogonal polarization components. When the phase difference approaches π/2, the metasurface functions as a quarter‐wave plate; when it approaches π, it functions as a half‐wave plate. In 2012, Yu et al. proposed a broadband background‐suppressed plasmonic metasurface quarter‐wave plate using displaced V‐antenna subunits, converting linearly polarized light into circularly polarized light with nearly equal amplitudes and an approximately π/2 phase difference [64], as shown in Figure 2e. In 2018, Zhu et al. designed an all‐dielectric metasurface based on Mie‐type electromagnetically induced transparency for terahertz linear‐to‐circular polarization conversion [65].

Control of polarization‐conversion efficiency constitutes another key mechanism of metasurface polarization modulation. In 2013, Grady et al. demonstrated ultrathin terahertz metamaterial polarization converters that achieved broadband and high‐efficiency linear polarization conversion by exploiting constructive interference of the cross‐polarized components and destructive interference of the co‐polarized components in a Fabry–Pérot‐like cavity [66]. In 2015, Ding et al. proposed a plasmonic metasurface half‐wave plate with broadband, efficient, background‐suppressed linear polarization conversion [67]. In 2016, Kruk et al. designed broadband, highly transmissive all‐dielectric metasurfaces based on multipolar interference and the generalized Huygens principle to realize efficient half‐ and quarter‐wave plates, as shown in Figure 2f [68]. Overall, polarization‐conversion efficiency can be effectively enhanced by engineering multiple‐interference effects, employing supercell‐based phase‐gradient designs, and exploiting multipolar interference in dielectric meta‐atoms.

Beyond conversion efficiency, polarization‐selective response provides an additional degree of freedom for metasurface polarization manipulation. By tailoring the anisotropy, chirality, or spatial orientation of meta‐atoms, metasurfaces can impart distinct phase, amplitude, and polarization‐conversion responses to different incident polarization states. In 2015, Arbabi et al. used high‐contrast dielectric elliptical nanopillars to efficiently control phase and polarization, demonstrating polarization splitting, polarization‐switchable holography, and vector‐beam generation [69]. In 2022, Chen et al. designed a reflective terahertz metasurface with a relative bandwidth of 94% and a conversion efficiency exceeding 90%, enabling broadband linear‐to‐linear, linear‐to‐circular, and circular‐to‐linear polarization conversion [70].

#### 2.2.4 Wavelength Control

Wavelength is another fundamental degree of freedom. Different wavelengths correspond to distinct spectral channels, and meta‐atom transmission, reflection, absorption, and scattering responses are intrinsically wavelength‐dependent. To explicitly describe the wavelength dependence of the metasurface response, Equation (5) can be generalized as:

(8) $$\begin{array}{c} E\left(x,y,\lambda \right)=A\left(x,y,\lambda \right)exp\left[i\Phi \left(x,y,\lambda \right)\right] \end{array}$$

where *A*(*x*, *y*, λ) and Φ(*x*, *y*, λ) denote the wavelength‐dependent amplitude and phase responses at each spatial position, respectively; λ denotes the optical wavelength. By designing geometry, material dispersion, and resonant behavior, metasurfaces can selectively manipulate light at different wavelengths. The central task is to engineer spectral and dispersion responses for wavelength‐selective absorption, spectral filtering, structural color, dynamic spectral modulation, and wavelength‐selective information encoding.

Resonance‐based spectral selectivity constitutes one of the primary routes to wavelength control. When the incident wavelength approaches a plasmonic, Mie‐type, or Fano resonance supported by the metasurface, its transmission, reflection, absorption, or scattering response can vary markedly with wavelength, thereby enabling selective control over specific wavelengths or spectral bands. In 2011, Aydin et al. reported an ultrathin plasmonic superabsorber based on metal‐dielectric‐metal resonances, achieving broadband polarization‐independent absorption in the visible range [71]. In 2012, Kats et al. proposed an ultrathin tunable perfect absorber based on VO_2_ phase‐change materials, using the insulator–metal transition to dynamically tune the absorption spectrum [72]. In 2016, Proust et al. employed electric and magnetic Mie‐type resonances supported by silicon nanoparticles to realize all‐dielectric structural coloration across the visible spectrum [73]. In the same year, Campione et al. introduced symmetry‐broken dielectric resonators to generate high‐Q Fano resonances with narrow spectral features, providing an effective route toward highly selective spectral control [74]. Overall, plasmonic resonances, dielectric Mie‐type resonances, Fano interference, and phase‐change materials provide complementary routes to broadband absorption, structural coloration, narrowband spectral selection, and dynamic wavelength control.

Structural color generation exemplifies the ability of metasurfaces to selectively control distinct wavelength channels. Its underlying principle is to tailor the geometrical dimensions, material refractive indices, and resonant modes of meta‐atoms such that different structural elements exhibit selective transmission, reflection, or scattering at prescribed wavelengths, thereby mapping distinct spectral channels onto visible colors and enabling the encoding of color and spectral information. In 2017, Sun et al. proposed a full‐color printing approach based on TiO_2_ dielectric metasurfaces. By varying the geometrical dimensions of the dielectric meta‐atoms, the electric and magnetic resonances in the visible regime were tailored so that different elements produced selective reflection peaks at target wavelengths, enabling low‐loss and high‐resolution structural color printing [75]. In the same year, Vashistha et al. developed all‐dielectric color pixels based on cross‐shaped silicon resonators. By independently varying the lengths and widths of the nanoantennas, the selective resonance wavelengths were tuned to achieve structural color generation with an extended color gamut [76]. In 2020, Yang et al. further proposed a high‐performance all‐dielectric structural‐color metasurface, in which the synergistic design of a silicon metasurface and an index‐matching layer reduced background reflection and optimized the reflection spectrum, resulting in structural colors with high brightness, high saturation, and high spatial resolution [77]. Engineering the resonant modes and wavelength‐selective scattering responses of dielectric meta‐atoms enables precise control over the intensity of individual spectral channels, thereby supporting structural color generation, spectral filtering, and wavelength‐selective information encoding.

#### 2.2.5 OAM Modulation

Beyond phase, amplitude, polarization, and wavelength, the spatial mode of an optical field constitutes another important degree of freedom for light‐field manipulation. In particular, vortex beams with an azimuthal phase factor exp(*il*φ) can carry OAM, and their helical phase distribution is commonly expressed as follows:

(9) $$\begin{array}{c} \Phi_{\mathrm{OAM}}\left(x,y\right)=l\varphi \end{array}$$

where Φ_OAM_(*x*,*y*) denotes the OAM‐associated helical phase distribution, *l* denotes the topological charge, and φ is the azimuthal angle. For l≠0, the vortex beam exhibits a phase singularity at its center and typically possesses an annular intensity profile [78]. Under ideal conditions, OAM modes with different topological charges form a mutually orthogonal mode set, thereby providing an additional spatial‐mode degree of freedom for structured‐light generation, optical communications, information multiplexing, optical manipulation, and high‐dimensional light‐field encoding.

Helical phase encoding provides the most direct route to metasurface‐based OAM modulation. By constructing an azimuthally varying phase distribution across a two‐dimensional interface, a metasurface can convert an incident plane wave or Gaussian beam into an OAM‐carrying vortex beam. In 2012, Genevet et al. proposed an ultrathin plasmonic optical vortex plate based on phase discontinuities, enabling the generation of vortex beams with different topological charges [49]. This work demonstrated that ultrathin planar metasurfaces can provide a compact alternative to conventional bulk spiral phase plates, offering an effective route toward integrated OAM beam generation. Beyond OAM generation, metasurfaces can also be employed for OAM mode detection. In 2016, Jin et al. designed a metasurface comprising an array of elliptical nanoholes, which converted circularly polarized incident light into an opposite‐helicity vortex beam and enabled OAM topological‐charge detection through interference with an integrated reference wave [79]. As shown in Figure 2g, the central region generates the vortex beam, whereas the surrounding annular region produces a spherical reference wave. The resulting spiral interference fringes allow both the magnitude and sign of the topological charge to be identified, demonstrating that metasurfaces can simultaneously perform OAM mode generation and recognition.

PB‐phase also provides an important route to spin‐dependent OAM modulation. As described by Equation (6), the PB‐phase is directly determined by the in‐plane orientation of anisotropic meta‐atoms. When the orientation angle is varied azimuthally across the metasurface, the resulting PB‐phase forms a helical phase distribution, thereby enabling spin‐dependent OAM beam generation. In 2014, Karimi et al. experimentally demonstrated PB‐phase‐mediated spin‐to‐orbital angular momentum conversion in the visible regime using an array of plasmonic gold nanoantennas with spatially varying orientations [80]. The metasurface converted the incident circularly polarized light into an opposite‐helicity component and imparted a topological charge of *l*  =   ± 2, with the sign determined by the handedness of the incident polarization. In 2017, Liu et al. designed planar PB‐phase elements for generating perfect vortex beams and further extended the approach to the generation of perfect vector beams [81]. This work showed that PB‐phase elements can integrate vortex‐ and vector‐beam generation within planar optical structures, providing an effective route toward spin‐dependent OAM modulation and integrated structured‐light sources.

Beyond spin‐dependent OAM generation, metasurfaces have also been developed to support OAM multiplexing, demultiplexing, and multichannel generation. In 2017, Li et al. proposed a single‐metasurface scheme based on off‐axis phase design for OAM multiplexing and demultiplexing, demonstrating the feasibility of combining and separating distinct OAM channels through spatial phase encoding [82]. In 2022, P. Liu et al. proposed a monolayer reflective metallic metasurface composed of semicircular nanoslits [83]. By employing detour‐phase encoding, the device enabled the generation of individual OAM modes, OAM arrays, and spin‐dependent OAM states, with a maximum experimental conversion efficiency of 30.44%. In the same year, W. Liu et al. developed a multichannel terahertz quasi‐perfect vortex‐beam generator based on a multifunctional all‐dielectric metasurface [84]. By superimposing multiple spatial phase functions, the device generated and focused quasi‐perfect vortex beams with different topological charges in distinct spatial channels, while providing a controllable divergence angle that was largely independent of the topological charge. By optimizing the meta‐atom geometry, spatial phase distribution, and channel‐multiplexing strategy, metasurfaces can therefore enable high‐efficiency, multimode, and multichannel OAM manipulation.

Metasurface‐based OAM modulation supports vortex‐beam generation, topological‐charge control, mode detection, and multichannel multiplexing, while offering compact form factors, high design flexibility, and compatibility with photonic integration. Nevertheless, further improvements are required in mode purity, conversion efficiency, inter‐channel crosstalk, operating bandwidth, and the stability and robustness of high‐order OAM modes under practical propagation conditions. OAM modulation extends metasurface‐based light‐field manipulation into the spatial‐mode dimension and, together with phase, amplitude, polarization, and wavelength control, forms an important foundation for multidimensional light‐field manipulation.

The physical mechanisms, representative implementations, key performance metrics, advantages, and limitations of individual optical degrees of freedom are comparatively summarized in Table 1.

**TABLE 1:** Comparison of mechanisms and characteristics of metasurface‐based manipulation of individual optical degrees of freedom.

Optical degree of freedom	Control objective	Underlying physical mechanism	Typical structures and materials	Key design parameters	Representative functions	Key performance metrics	Advantages and limitations	Refs.
Phase	Full 0–2*π* phase coverage and prescribed spatial phase‐profile control	Propagation‐phase; PB‐phase; resonance‐mediated phase response via Fabry–Pérot/Mie resonances and Huygens‐type multipolar interference	High‐index dielectric nanopillars/nanofins and metallic anisotropic nanoantennas	Meta‐atom geometry, height, period, orientation angle, and resonant mode	Beam steering, focusing and collimation, wavefront shaping, and structured‐light generation	Phase coverage, modulation efficiency^a^, wavefront error, operating bandwidth, and quantization error	Advantages: subwavelength control; ultrathin form factor; high design flexibility Limitations: strong dispersion; phase‐quantization error; coupling and fabrication sensitivity; limited PB‐phase efficiency	[37, 39, 40, 54, 80]
Amplitude	Control of transmitted/reflected intensity and spatial energy distribution	Dissipative/resonant amplitude control: resonant absorption and critical coupling; scattering‐mediated control: redistribution among scattering channels; polarization‐mediated control: polarization‐dependent interference/projection in anisotropic meta‐atoms; active‐material tuning where applicable	Metal–dielectric–metal absorbers, dielectric birefringent meta‐atoms, and graphene‐ or VO_2_‐based tunable metasurfaces	Meta‐atom geometry, material loss, and impedance matching	Grayscale display, intensity shaping, energy distribution, and dynamic modulation	Modulation depth, extinction ratio, grayscale levels, operating bandwidth, and inter‐channel crosstalk	Advantages: direct control of intensity and energy distribution; support for complex‐amplitude encoding Limitations: absorption loss, channel dependence, and incomplete amplitude‐phase decoupling	[60, 61, 62, 63]
Polarization	Control of polarization state, polarization conversion, and polarization‐selective response	Anisotropy‐induced birefringent phase retardation between orthogonal eigenpolarizations; polarization‐channel interference between co‐ and cross‐polarized components; multipolar/Huygens interference for high‐efficiency polarization conversion	Dielectric elliptical nanopillars, silicon nanopillars, and plasmonic V‐shaped/nanobrick antenna arrays	Birefringence, phase delay between orthogonal components, and orientation angle	Ultrathin waveplates, polarization conversion, polarization beam splitting, and vector‐beam generation	Polarization‐conversion efficiency, polarization purity, transmittance/reflectance, and operating bandwidth	Advantages: ultrathin integration of polarization conversion, polarization selectivity, and wavefront control Limitations: input‐polarization dependence, stringent amplitude‐phase matching between orthogonal components, and fabrication sensitivity	[64, 67, 68, 69]
Wavelength	Wavelength‐selective spectral response and dynamic spectral tuning	Resonance‐mediated spectral selectivity: plasmonic, Mie‐type, and Fano resonances; material‐state‐dependent spectral modulation: changes in refractive index and/or loss induced by active or phase‐change materials	Si/TiO_2_ dielectric resonators, metal–dielectric–metal absorbers, and VO_2_‐based phase‐change platforms	Meta‐atom geometry, period, and refractive index	Structural color printing, spectral filtering, wavelength‐selective information encoding, and dynamic spectral tuning	Resonance wavelength, peak reflectance/absorption, spectral linewidth, tuning range, and color gamut	Advantages: wavelength‐selective responses enable structural color generation and tunable spectral control Limitations: limited resonant bandwidth, efficiency‐linewidth trade‐offs, and spectral shifts induced by material dispersion and fabrication errors	[71, 72, 73, 74, 75, 77]
OAM	Generation, conversion, multiplexing/demultiplexing, and detection of OAM modes	Azimuthal phase winding and phase singularity associated with the helical phase *e* ^*il*φ^ for OAM generation; spin–orbit interaction mediated by PB‐phase for spin‐dependent OAM conversion; modal orthogonality and interferometric phase comparison for OAM multiplexing/demultiplexing and detection	V‐shaped plasmonic nanoantennas, metallic elliptical nanoapertures and nanogrooves, and all‐dielectric silicon meta‐atoms	Azimuthal phase, topological charge, and radial phase	Vortex‐beam generation, topological‐charge control, OAM multiplexing and demultiplexing, and OAM mode detection	OAM mode purity, conversion efficiency, inter‐channel crosstalk, and channel count	Advantages: Orthogonal OAM modes enable high‐dimensional multiplexing and increased channel capacity Limitations: Limited conversion efficiency and mode purity; inter‐channel crosstalk; mode‐dependent divergence; reduced robustness of high‐order modes	[49, 79, 80, 82, 83, 84]

^a^ Depending on the device type, modulation efficiency may refer to transmission efficiency, reflection efficiency, or target‐channel conversion efficiency.

## 3 Multidimensional Manipulation Strategies

### 3.1 Joint Phase‐Amplitude Control

In complex light‐field design, controlling phase or amplitude alone is generally insufficient to simultaneously satisfy the requirements of wavefront accuracy, spatial energy distribution, and reconstruction fidelity. As expressed in Equation (5), an arbitrary monochromatic scalar optical field is jointly characterized by its amplitude *A*(*x*, *y*) and phase Φ(*x*, *y*), where the local intensity satisfies *I*(*x*, *y*)  =  |*A*(*x*, *y*)|^2^, while the phase governs optical propagation, interference, and wavefront evolution. The central objective of joint phase‐amplitude control is therefore to establish, within a single metasurface, a mapping between the geometrical parameters of the meta‐atoms and the desired complex transmission or reflection coefficients, while minimizing residual coupling between amplitude and phase. Compared with phase‐only modulation, complex‐amplitude modulation enables more faithful reconstruction of the target optical field and provides a foundation for improving holographic fidelity, optimizing energy distribution among diffraction channels, and generating complex structured light.

A representative strategy for complex‐amplitude encoding is to decouple amplitude and phase by jointly tailoring the geometrical parameters and orientation angles of the meta‐atoms. In 2018, Lee et al. proposed a broadband complex‐amplitude holographic metasurface based on X‐shaped meta‐atoms formed by superimposing two nanorods with different orientation angles [85]. For the cross‐polarized transmitted light, the amplitude was predominantly determined by the difference between the two orientation angles, whereas the phase was governed by their sum, thereby enabling simultaneous amplitude and phase modulation and complex‐amplitude light‐field encoding in the visible regime, as shown in Figure 3a. In 2019, Overvig et al. developed an all‐dielectric metasurface in which the form birefringence and rotation angle of the dielectric meta‐atoms were engineered to establish a direct mapping between the target amplitude and phase and the corresponding structural parameters, enabling complete and independent control of the optical amplitude and phase [86]. As illustrated in Figure 3b, the geometrical parameters of the meta‐atoms were used to tailor the polarization‐conversion amplitude, while their rotation angles controlled the output phase, providing an effective route to independent complex‐amplitude encoding on a low‐loss dielectric platform. In 2022, Jiang et al. proposed an ultrathin dielectric metasurface in which the geometrical dimensions of the nanorods were varied to tailor the multipolar resonances and hence the output amplitude, while the phase was controlled through the PB‐phase, ultimately enabling arbitrary complex‐amplitude encoding in transmission at visible wavelengths [87]. Collectively, these studies demonstrate that the coordinated design of meta‐atom orientation angles, geometrical dimensions, form birefringence, multipolar resonances, and the PB‐phase can enable relatively independent amplitude and phase modulation within specified input‐output polarization channels, thereby establishing an important foundation for high‐fidelity holography, complex wavefront shaping, and complete optical‐field reconstruction.

**FIGURE 3:**
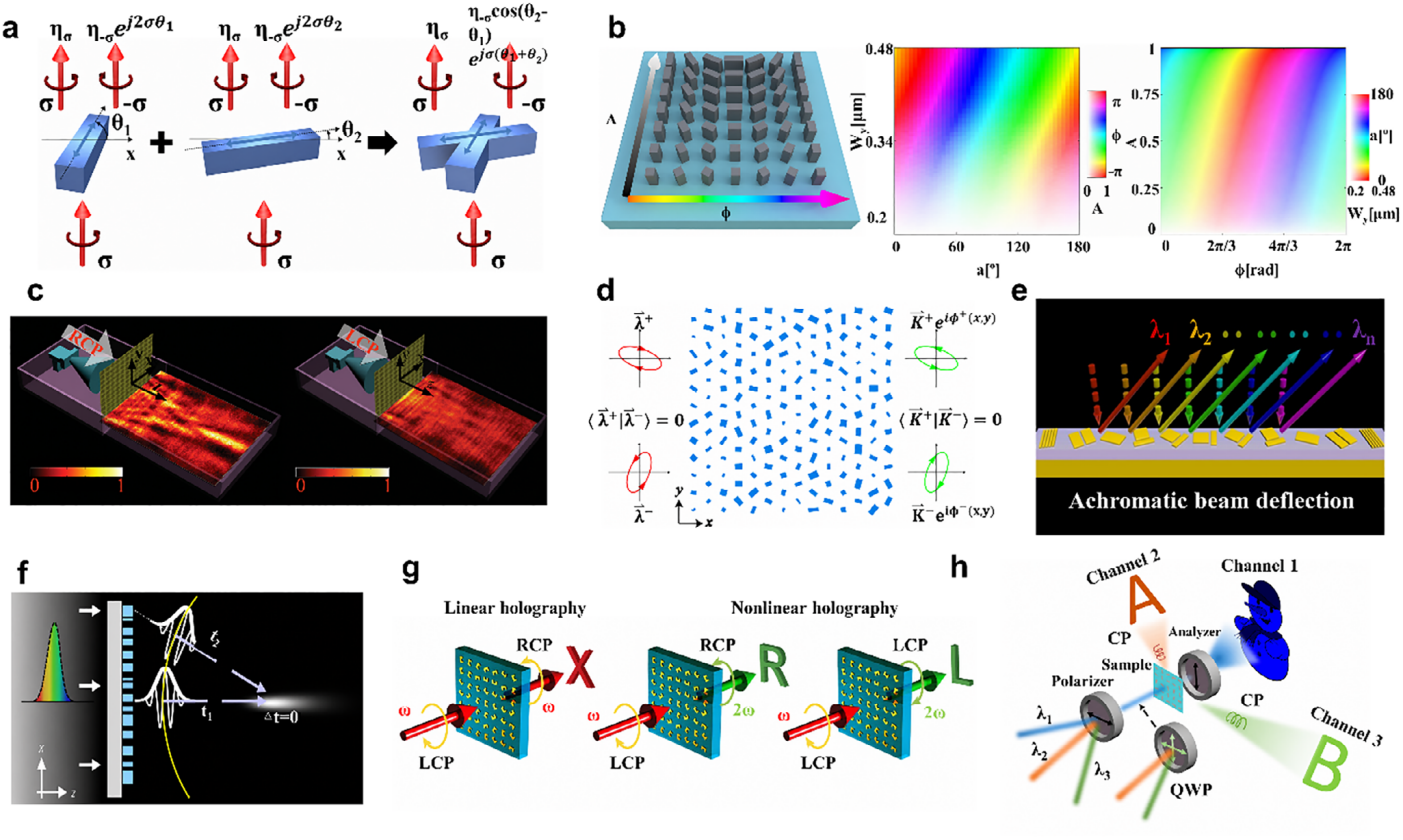
Strategies for joint control of phase, amplitude, polarization, and wavelength in metasurfaces. (a) Principle of broadband complex‐amplitude control using X‐shaped meta‐atoms. Reprinted with permission [85]. Copyright 2018, The Royal Society of Chemistry. (b) Independent amplitude‐phase encoding through shape birefringence and rotation angle. Reprinted under the terms of the CC BY 4.0 license [86]. Copyright 2019, The Authors. (c) Handedness‐controlled bifunctional converging/diverging Pancharatnam–Berry (PB) phase metalens. Reprinted with permission [98]. Copyright 2014, WILEY‐VCH Verlag GmbH & Co. KGaA, Weinheim. (d) Independent phase control for arbitrary orthogonal polarization states. Reprinted with permission [20]. Copyright 2017, American Physical Society. (e) Achromatic beam deflection at a wavelength‐independent angle. Reprinted under the terms of the CC BY 4.0 license [108]. Copyright 2017, The Authors. (f) Phase and group‐delay compensation in broadband achromatic metalenses. Reprinted with permission [109]. Copyright 2018, The Authors, under exclusive licence to Macmillan Publishers Limited, part of Springer Nature. (g) Readout channels in spin‐ and wavelength‐multiplexed nonlinear metasurface holography. Reprinted under the terms of the CC BY 4.0 license [116]. Copyright 2016, The Authors. (h) Three‐channel multiwavelength nanoprinting and holography. Reprinted under the terms of the CC BY 4.0 license [117]. Copyright 2022, The Authors.

Building on single‐channel complex‐amplitude decoupling, research has increasingly shifted toward constructing multiple mutually independent complex‐amplitude channels by exploiting polarization, frequency, and output polarization components, thereby increasing the information capacity and functional density of metasurface devices. In 2021, Liu et al. proposed a transmission‐mode all‐dielectric multifunctional metasurface platform in which single‐layer TiO_2_ linearly birefringent nanopillars were assembled into super‐pixels [88]. By using a Jones‐matrix framework to establish the mapping between the target complex‐amplitude responses and the nanopillar geometrical dimensions and orientation angles, the platform enabled simultaneous and independent control of amplitude and phase for two orthogonal polarization states. Its multifunctionality was further demonstrated through polarization‐switchable grayscale nanoprinting, nonuniform cylindrical lensing, and complex‐amplitude holography. In the same year, Zheng et al. developed an all‐dielectric dual‐channel complex‐amplitude metasurface that combined propagation‐phase with the PB‐phase to independently modulate amplitude and phase in two circular‐polarization channels, and demonstrated switchable, power‐controllable bifocal metalenses [89]. In 2022, Xie et al. proposed a dual‐channel PB‐phase metasurface based on bi‐spectral, single‐substrate‐layer meta‐atoms containing two nearly decoupled resonant elements [90]. Independent PB‐phase modulation and multilevel amplitude modulation were achieved at 10 and 15 GHz, respectively, and experimentally validated through dual‐frequency complex‐amplitude holographic reconstruction. In 2023, Yang et al. further demonstrated a terahertz multichannel all‐dielectric metasurface that encoded multiple sets of complex‐amplitude information into the co‐ and cross‐polarization channels under orthogonal linear‐polarization incidence, thereby enabling independent multichannel control of amplitude and phase [91]. Overall, polarization‐basis multiplexing, frequency‐selective resonant decoupling, and the coordinated design of propagation and PB‐phases provide effective routes for increasing the number of complex‐amplitude channels and expanding the functional degrees of freedom of metasurface devices.

Joint phase‐amplitude control can also be exploited to optimize diffractive energy allocation and improve holographic reconstruction quality. In 2018, Song et al. proposed a complex‐amplitude selective‐diffraction scheme based on a dielectric metasurface [92]. By simultaneously tailoring the amplitude and phase responses of the meta‐atoms, the approach selectively enhanced the desired diffraction orders while effectively suppressing undesired ones. In 2019, Jiang et al. further employed a complex‐amplitude metasurface to improve holographic image quality, demonstrating that complex‐amplitude holography can encode both the amplitude and phase information of the target light field [93]. Compared with phase‐only holography, this approach effectively suppresses zero‐order noise and twin‐image artifacts, thereby improving the fidelity of the reconstructed image. Collectively, these studies demonstrate that joint phase‐amplitude control not only improves the reconstruction accuracy of complex light fields, but also enables more efficient energy allocation, flexible redistribution among diffraction channels, and reduced crosstalk from undesired channels.

Joint phase‐amplitude control is advancing along three complementary directions: broadband response, dynamic reconfigurability, and data‐driven design. In 2022, Hao et al. reported a broadband transmissive amplitude‐and‐phase metasurface in which the opening angle and orientation angle were tailored to control the amplitude and phase, respectively, of the cross‐polarized transmitted wave, thereby achieving full complex‐amplitude coverage over a broad frequency range [94]. The proposed scheme was further applied to high‐purity vortex‐beam generation and dual‐plane holography, demonstrating that complex‐amplitude modulation can simultaneously improve the mode quality of structured optical fields and the fidelity of holographic reconstruction. In 2024, Han et al. developed a guided‐wave‐driven programmable complex‐amplitude metasurface by integrating p‐type‐intrinsic‐n‐type (PIN) diodes into the meta‐atoms, enabling 1‐bit amplitude switching between radiating and nonradiating states and 1‐bit phase switching between 0° and 180° [95]. Using the same low‐profile platform, low‐sidelobe‐level beam scanning and Airy‐beam generation were experimentally demonstrated. In the same year, Miao et al. introduced neural networks into the design of polarization‐multiplexed complex‐amplitude holograms [96]. Their end‐to‐end framework established a direct mapping between the geometrical dimensions of the meta‐atoms and the reconstructed images, while independently encoding amplitude and phase distributions into the left‐ and right‐handed circular polarization channels, thereby improving the reconstruction quality of dual‐channel holography. Collectively, these studies have advanced complex‐amplitude metasurfaces in terms of spectral coverage, electrically programmable functionality, and the efficiency of high‐dimensional design and optimization. Accordingly, the field is evolving from fixed, single spatial complex‐transmission functions toward broadband operation, electrically reconfigurable complex‐amplitude encoding, and data‐driven multichannel inverse design. Future progress will rely on the integration of multibit modulation, low‐loss broadband meta‐atoms, closed‐loop feedback, and fast optimization algorithms, enabling spatial complex responses to be updated in real time, with high precision and across multiple channels, according to the target light field.

Joint phase‐amplitude control is evolving from static, single‐channel complex‐field encoding toward broadband, multichannel, and reconfigurable complex responses, thereby enabling more complete light‐field manipulation for high‐fidelity holography and complex structured‐light generation.

### 3.2 Joint Phase‐Polarization Control

In multifunctional metasurface design, polarization serves not only as a degree of freedom to be manipulated, but also as an independent information channel for differentiating distinct wavefront functionalities. By encoding tailored phase profiles for different incident or output polarization states, a single metasurface can enable polarization multiplexing, functional switching, spin‐selective focusing, polarization‐dependent holography, and multichannel wavefront control. The joint manipulation of phase and polarization therefore provides a powerful strategy for increasing the information capacity and functional integration of metasurfaces.

PB‐phase provides a fundamental basis for the joint control of phase and polarization. In 2001, Bomzon et al. extended the concept of the PB‐phase from the evolution of spatially uniform polarization states to spatially varying polarization transformations [97]. They demonstrated that spatial variations in the local polarization state are accompanied by a position‐dependent PB‐phase and experimentally verified, using subwavelength gratings, its influence on the far‐field distribution of vector beams. In 2015, Ding et al. developed an ultrathin PB‐phase metasurface with a thickness of approximately *λ*/1000, achieving a cross‐polarized anomalous‐transmission efficiency of approximately 24.7% in the microwave regime‐close to the theoretical upper limit of 25% for an infinitesimally thin, passive, reciprocal, single‐layer metasurface [98]. As shown in Figure 3c, the same metalens generated a converging wavefront under right‐handed circularly polarized illumination and a diverging wavefront under left‐handed circularly polarized illumination, thereby enabling helicity‐controlled switching between focusing and defocusing. Collectively, these studies establish that the PB‐phase can map the helicity of circularly polarized light onto distinct wavefront responses through the spatial orientation of meta‐atoms, providing a simple yet effective route to spin‐selective control and polarization multiplexing. In practice, however, the attainable modulation efficiency is primarily limited by the cross‐polarization conversion efficiency.

Building on the cross‐circular‐polarization conversion mechanism described above, rotated anisotropic meta‐atoms impart PB‐phases of opposite signs to circularly polarized light with opposite helicities. By engineering the spatial orientation of the meta‐atoms, left‐ and right‐handed circularly polarized waves can therefore be encoded with distinct, or even mutually inverted, phase profiles, enabling spin‐dependent wavefront control. In 2012, Chen et al. proposed a bipolar plasmonic metalens operating in the visible regime [99]. By exploiting helicity‐dependent phase discontinuities, the same planar metalens functioned as either a positive or a negative lens under opposite circularly polarized illumination, thereby realizing spin‐selective focusing. In 2013, Yin et al. observed the photonic spin Hall effect in a metasurface, demonstrating that a phase‐discontinuous interface can induce spin‐dependent beam steering and providing experimental evidence for metasurface‐enabled control of photonic spin‐orbit interactions [100]. Together, these studies show that polarization can be coupled to phase modulation such that a single metasurface supports distinct wavefront functionalities in different circular‐polarization channels, offering a versatile route to spin‐selective focusing, polarization multiplexing, and spin‐dependent wavefront engineering.

However, PB‐phase modulation alone generally imparts PB‐phases of opposite signs to left‐ and right‐handed circularly polarized channels. The resulting conjugate or mirror‐symmetric relationship between the two spin channels constrains their fully independent design. To overcome this limitation, propagation and PB‐phases have been combined such that the propagation‐phase controls the common phase component of the two circular‐polarization channels, whereas the PB‐phase determines their differential phase. This concept can be expressed as:

(10) $$\begin{array}{c} \Phi_{\pm }\left(x,y\right)=\Phi_{d}\;\left(x,y\right)\pm 2\theta \left(x,y\right) \end{array}$$

where Φ_d_(*x*,*y*) denotes the propagation‐phase profile introduced by the meta‐atom geometry, θ(*x*, *y*) is the spatial distribution of the meta‐atom orientation angle, and Φ_±_(*x*,*y*) denotes the phase responses of the two opposite circular‐polarization channels. Accordingly, the propagation‐phase and orientation angle can be determined from the average and half‐difference, respectively, of the two target phase profiles, thereby enabling independent wavefront encoding in the two spin channels. In 2017, Devlin et al. introduced a dielectric J‐plate metasurface in which the birefringent propagation‐phase and optical‐axis orientation of the meta‐atoms were jointly engineered to convert two orthogonal spin states into OAM states with independently specified topological charges [101]. This approach overcame the restriction of conventional PB‐phase elements, which typically generate conjugate pairs of OAM modes. This advance demonstrated that the synergistic control of propagation and PB‐phases can remove the intrinsic phase correlation between opposite circular‐polarization channels, enabling independent mapping between optical spin states and spatial modes. In 2020, Xu et al. extended this spin‐decoupling strategy to an all‐dielectric terahertz metasurface [102]. By simultaneously tailoring the structural dimensions and rotation angles of the meta‐atoms, they independently encoded the phase profiles of the two circularly polarized components and generated terahertz vector beams with customizable polarization states and phase structures. In 2022, Wu et al. further implemented the combined control of propagation and PB‐phases on an all‐silicon terahertz platform and experimentally demonstrated two classes of spin‐multiplexed metasurfaces [103]. One independently produced a Bessel beam and half‐wave‐plate‐like polarization conversion under orthogonal circularly polarized illumination, whereas the other generated a Bessel beam and a vortex beam in the two respective spin channels. These results further highlighted the capability of low‐loss dielectric platforms to integrate spin‐selective polarization transformations with spatial wavefront functionalities.

Beyond circular‐polarization channels, orthogonal linear‐polarization states can also serve as independent information carriers for wavefront encoding. In 2021, Wang et al. proposed an all‐dielectric metasurface comprising asymmetric, polarization‐decoupled meta‐atoms [104]. By independently tailoring the geometrical parameters of each meta‐atom along two orthogonal axes, they achieved independent control over the propagation phases of the *x*‐ and *y*‐polarized channels, and demonstrated independent asymmetric deflection of orthogonally polarized beams together with dual‐channel polarization‐multiplexed holography. More generally, Jones‐matrix‐based design provides a unified theoretical framework for independent phase modulation over arbitrary pairs of orthogonal polarization states. In 2017, Mueller et al. introduced a scheme based on linearly birefringent metasurface elements that enables independent phase profiles to be imparted to orthogonal polarization channels, including linear, circular, and elliptical polarization states [20]. By combining propagation and PB‐phases, this approach overcame the inherent constraints imposed by conventional PB‐phase modulation on channel independence, thereby enabling independent phase encoding across distinct polarization channels. As illustrated in Figure 3d, this strategy substantially expands the design degrees of freedom available for arbitrary‐polarization multiplexing and multichannel wavefront control.

Beyond the polarization‐conversion limitation associated with PB‐phase modulation, joint phase‐polarization control is further constrained by the finite structural degrees of freedom available for simultaneously satisfying multiple polarization‐dependent responses. In spin‐decoupled or linear‐polarization‐decoupled designs, these constraints can lead to residual inter‐channel crosstalk, restricted operating bandwidth, and increased sensitivity to fabrication tolerances. More general Jones‐matrix engineering expands the accessible design space, but can also increase structural complexity and fabrication demands. These constraints have motivated the development of more flexible matrix‐based and high‐capacity polarization‐multiplexing strategies.

In recent years, joint phase‐polarization control has evolved beyond phase multiplexing in a limited set of polarization channels toward full‐parameter Jones‐matrix engineering, high‐capacity multiplexing, and on‐chip integration. Unlike conventional approaches that assign independent phase profiles to different polarization channels, full‐parameter Jones‐matrix control seeks to simultaneously tailor the amplitude and phase of each complex matrix element, thereby enabling more general polarization transformations, polarization multiplexing, and vectorial wavefront manipulation. In 2022, Bao et al. introduced a bilayer metasurface that achieved full‐parameter Jones‐matrix control by exploiting additional structural degrees of freedom and interlayer coupling [105]. This study showed that expanding the accessible structural parameter space can overcome the limited controllability of single‐layer polarization‐multiplexed metasurfaces, offering a more versatile platform for arbitrary polarization conversion and complex wavefront engineering. Building on this expansion of design freedom, subsequent efforts have increasingly focused on on‐chip integration, high‐capacity multiplexing, and intelligent optimization. In 2024, Ji et al. proposed an on‐chip, full‐parameter, multiplexed Jones‐matrix metasurface [106]. By combining detour, geometric, and propagation phases, they independently controlled the four complex elements of the effective Jones matrix and demonstrated multichannel amplitude‐phase modulation together with direction‐multiplexed holography. Deep‐learning approaches have also been employed to explore the limits of polarization multiplexing in metasurfaces, enabling increasingly complex multichannel designs through data‐driven optimization [107]. Joint phase‐polarization control is thus progressing from phase multiplexing across a finite number of polarization channels toward a unified matrix‐based design of polarization transformations, complex‐amplitude wavefronts, and propagation directions. Further advances will depend on achieving a more favorable balance among controllable degrees of freedom, conversion efficiency, inter‐channel crosstalk, operating bandwidth, and fabrication complexity.

### 3.3 Joint Phase‐Wavelength Control

In broadband and multispectral light‐field manipulation, the phase response of a metasurface is inherently wavelength‐dependent and therefore subject to dispersion. When the phase profiles at different wavelengths fail to satisfy the prescribed wavefront requirements, focal shifts, wavefront distortions, and inter‐channel crosstalk can arise. The central objective of joint phase‐wavelength control is therefore to treat wavelength or angular frequency as an explicit design variable and engineer a spatial‐spectral phase response. Depending on how the responses at different wavelengths are related, this strategy can be broadly classified into two paradigms. The first jointly engineers the phase and its frequency derivatives over a continuous spectral range, such that different wavelengths generate either an identical target wavefront or wavefronts that evolve according to a prescribed wavelength‐dependent trajectory. This approach underpins broadband achromatic focusing, imaging, and beam steering. The second encodes mutually independent or approximately independent spatial phase profiles into a discrete set of wavelength channels, enabling multiwavelength wavefront control, spectral‐channel multiplexing, and wavelength‐selective information retrieval.

From a physical perspective, the spatial‐spectral phase response Φ(*x*, *y*, ω) of a metasurface can be Taylor‐expanded in angular frequency ω about the design angular frequency ω_0_ as:

(11) $$\begin{array}{c} \Phi \left(x,y,\omega \right)=\Phi \left(x,y,\omega_{0}\right)+\left(\omega -\omega_{0}\right)\tau \left(x,y\right)+\frac{1}{2}{\left(\omega -\omega_{0}\right)}^{2}GDD\left(x,y\right)+\cdots \end{array}$$

where the first‐ and second‐order derivatives of the spectral phase with respect to angular frequency correspond to the group delay τ(*x*, *y*) and group‐delay dispersion *GDD*(*x*, *y*), respectively:

(12) $$\tau \left(x,y\right)={\left.\frac{\partial \Phi (x,y,\omega )}{\partial \omega }\right|}_{\omega =\omega_{0}},GDD\left(x,y\right)={\left.\frac{\partial^{2}\Phi \left(x,y,\omega \right)}{\partial \omega^{2}}\right|}_{\omega =\omega_{0}}$$

For an ideal achromatic metalens with a wavelength‐independent focal length *f* in free space, the target phase difference ΔΦ_lens_(*r*,ω) at radial coordinate *r*, referenced to the lens center, can be written as:

(13) $$\Delta \Phi_{\mathrm{lens}}\left(r,\omega \right)=\Phi \left(r,\omega \right)-\Phi \left(0,\omega \right)=-\frac{\omega }{c}\left(\sqrt{r^{2}+f^{2}}-f\right)$$

where $r=\sqrt{x^{2}+y^{2}}$ and *c* is the speed of light in vacuum. Equation (13) specifies the spatially differential phase relative to the lens center, which determines the focusing wavefront of the metalens. By differentiating Equation (13) once and twice with respect to angular frequency, respectively, and applying the definitions in Equation (12), the relative group delay Δτ(*r*) and relative group‐delay dispersion Δ*GDD*(*r*) required for an ideal achromatic metalens with a fixed focal length are obtained as follows:

(14) $$\Delta \tau \left(r\right)=-\frac{1}{c}\left(\sqrt{r^{2}+f^{2}}-f\right)$$

(15) $$\begin{array}{c} \Delta GDD\left(r\right)=0 \end{array}$$

The optical‐path‐length difference between different radial positions and the focal point therefore requires the device to provide a spatially varying group delay. By contrast, because the target phase of an ideal fixed‐focal‐length metalens depends linearly on frequency, its spatially differential group‐delay dispersion vanishes in principle. In practical implementations, however, meta‐atoms generally exhibit nonlinear resonant dispersion. Broadband designs must therefore constrain the second‐ and higher‐order spectral‐phase derivatives to suppress residual focal shifts and wavefront distortions. Moreover, when the target focal length varies with frequency, the propagation medium is appreciably dispersive, or more complex spectrally dependent wavefronts are required, the prescribed higher‐order dispersion may itself become spatially varying [108, 109].

Dispersion engineering constitutes a primary strategy for joint phase‐wavelength control. Early studies initially focused on achromatic focusing at a discrete set of selected wavelengths. In 2015, Khorasaninejad et al. employed a periodically arranged, coupled dielectric nanoresonators to independently tailor the scattering phases at 1300, 1550, and 1800 nm, thereby maintaining an identical focal length at all three design wavelengths [110]. Although the device operated only at discrete wavelengths rather than across a continuous spectrum, it provided an early experimental foundation for metasurface‐based dispersion compensation and represented an important step toward broadband achromatic flat optics. Subsequent efforts progressively extended dispersion control from discrete multiwavelength correction to continuous spectral engineering. In 2017, Wang et al. combined PB‐phase with wavelength‐dependent resonant‐phase compensation to realize broadband achromatic focusing and beam steering in the near‐infrared [108]. As shown in Figure 3e, incident light at different wavelengths was reflected by the metasurface at nearly the same anomalous deflection angle. In the same year, Arbabi et al. engineered the dispersion of dielectric metasurface optical elements to achieve positive, near‐zero, and strongly negative dispersion responses [111]. In 2018, Chen et al. reported a broadband visible achromatic metalens operating from 470 to 670 nm by simultaneously controlling the phase, group delay, and group‐delay dispersion [109], as illustrated in Figure 3f. Collectively, these advances drove achromatic metasurfaces from phase matching at a small number of discrete wavelengths toward spatial‐spectral phase and group‐delay engineering over continuous bandwidths, shifting the central design objective from wavelength‐by‐wavelength phase correction to coordinated control of the full spectral response.

To extend the accessible group‐delay range and improve the trade‐off between bandwidth and efficiency, multilayer and hybrid architectures have been increasingly adopted. In 2021, Wang et al. proposed a high‐efficiency broadband achromatic metalens based on bilayer nanopillars, in which the upper and lower layers predominantly provided the focusing phase and dispersion compensation, respectively [112]. The device achieved achromatic focusing over the 1000–1700 nm spectral range, with an average focusing efficiency of approximately 64%. In 2024, Chang et al. further introduced a dispersion‐matching layer to expand the attainable group‐delay range, enabling polarization‐insensitive achromatic focusing and full‐color imaging across the visible spectrum from 400 to 700 nm [113]. These studies demonstrate that increasing the structural degrees of freedom can substantially enlarge the accessible phase‐dispersion design space, albeit at the cost of greater fabrication complexity, more stringent interlayer‐alignment requirements, and increased sensitivity to interlayer coupling.

Joint phase‐wavelength control can also be exploited for spectral‐channel multiplexing. By encoding distinct spatial phase profiles into different wavelength channels, a metasurface can support multiwavelength wavefront manipulation and information multiplexing. In 2016, Wang et al. proposed a visible‐frequency dielectric metasurface in which nanostructures tailored to different operating wavelengths were integrated within a single layer [114]. This architecture enabled phase modulation across multiple spectral channels, including red, green, and blue, and was used to demonstrate both multiwavelength achromatic and highly dispersive holography. In 2019, Zhou et al. introduced a noninteracting multilayer dielectric metasurface, in which interlayer decoupling allowed individual functional layers to address distinct wavelength channels, thereby enabling relatively independent wavefront control at multiple wavelengths within a single device [115]. These studies demonstrate that wavelength‐selective meta‐atom engineering, spatial multiplexing, and multilayer decoupling can provide spectrally distinct channels with largely independent phase responses, thereby expanding the capabilities of metasurfaces for multiwavelength wavefront control and information multiplexing.

For multiwavelength information multiplexing and selective readout, wavelength channels can be combined with additional degrees of freedom—including spin, polarization, near‐ and far‐field distributions, and amplitude responses—such that a single metasurface outputs distinct information under different illumination wavelengths or polarization conditions. In 2016, Ye et al. proposed a nonlinear metasurface holography scheme based on joint spin‐wavelength multiplexing, in which multiple holographic images were encoded into independent channels defined by different spin states at the fundamental frequency and the second harmonic [116]. As shown in Figure 3g, a linear holographic image was reconstructed in the cross‐circularly polarized fundamental‐frequency channel, whereas independent holographic images were separately retrieved from distinct circular‐polarization channels at the second harmonic. This approach demonstrated the synergistic use of wavelength and spin degrees of freedom for multichannel information multiplexing. In 2022, Li et al. further introduced a three‐channel multiwavelength metasurface, using a linearly polarized channel for near‐field nanoprinting and two circularly polarized channels at different wavelengths for far‐field holographic display [117]. As illustrated in Figure 3h, different wavelength and polarization states served as independent readout channels, enabling the same metasurface to reconstruct near‐field nanoprinted images and far‐field holographic images under distinct illumination conditions. This architecture therefore realized joint near‐field–far‐field and multiwavelength information multiplexing.

In recent years, joint phase‐wavelength control has further advanced toward reconfigurable and data‐driven design. Achromatic metasurfaces based on the phase‐change material Ge_2_Sb_2_Te_5_ can dynamically tailor their spectral phase responses through refractive‐index modulation, providing a promising route toward reconfigurable dispersion engineering. Their performance, however, remains limited by optical absorption, cycling stability during phase transitions, and the precision with which local material states can be controlled [118]. In parallel, inverse‐design approaches based on deep‐learning architectures, including Transformers, can accelerate the mapping and optimization between meta‐atom spectral responses and target phase‐dispersion profiles, thereby improving the design efficiency of large‐scale broadband devices [119]. Their broader application will nevertheless require stronger incorporation of physical constraints, improved generalizability across different structural configurations, and greater robustness against fabrication imperfections. Joint phase‐wavelength control is therefore evolving from static dispersion compensation toward reconfigurable spectral responses and physics‐constrained intelligent inverse design.

### 3.4 Joint Phase‐OAM Control

For an ideal paraxial vortex field, the azimuthal phase factor *exp*(*il*φ)determines the topological charge of its OAM. Joint phase‐OAM control is not a simple superposition of two fully independent degrees of freedom. Rather, the helical phase encodes the OAM mode, while additional focusing, deflection, axicon, or holographic phases are incorporated to jointly tailor the topological charge, propagation direction, focal position, and radial intensity profile of the vortex beam. For a given channel m, the target phase can be expressed in a unified form as:

(16) $$\begin{array}{c} \Phi_{m}\left(r,\varphi \right)=l_{m}\;\varphi +\Phi_{\mathrm{aux},m}\left(r,\varphi \right)\;\left(mod\;2\pi \right) \end{array}$$

where m indexes the channels defined by polarization, wavelength, incidence direction, or spatial region; *l*_m_ denotes the topological charge associated with channel m; *l*_m_φ represents the corresponding helical OAM phase; and Φ_aux,m_(*r*, φ) denotes the auxiliary functional phase introduced for focusing, beam steering, axicon transformation, or holographic reconstruction. The total phase is wrapped modulo 2*π*. Within this composite‐phase framework, metasurfaces enable compact OAM‐beam generation, spatial mode shaping, and multichannel multiplexing and demultiplexing at the subwavelength scale.

At the level of functional integration, helical phase profiles are commonly combined with lens, beam‐steering, or other functional phase terms, extending OAM control beyond the generation of isolated vortex modes to include focusing, deflection, and spatial beam shaping. In 2021, Hu et al. developed a dielectric metasurface zone plate that integrated a Fresnel focusing phase with an OAM helical phase on a single platform, enabling the generation of focused vortex beams and providing a compact approach to phase‐encoded OAM‐field control [120]. In 2023, Liang et al. further introduced a reconfigurable metasurface whose programmable phase distribution allowed the near‐field focusing characteristics of vortex beams to be dynamically tailored, thereby enabling flexible control over focused vortex fields [121]. In 2025, Tian et al. proposed a visible‐light coding metasurface composed of TiO_2_ nanocylinders functioning as 3‐bit phase‐encoding elements [122]. By varying the nanocylinder radius, the platform achieved nearly 2*π* phase coverage together with high transmission efficiency. Different coding sequences were then superimposed to enable the generation, deflection, and focusing of vortex beams, as shown. This work demonstrates that coding‐based phase design can integrate OAM‐beam generation with simultaneous control over propagation direction and focusing characteristics within a single metasurface.

Beyond conventional vortex‐beam generation, perfect vortex beams represent another important direction in joint phase‐OAM control. Whereas the annular intensity radius of a conventional vortex beam typically varies with topological charge, that of a perfect vortex beam is, in principle, independent of the topological charge. This property facilitates the spatial overlap and multiplexing of multiple OAM modes. To realize such beams, helical, axicon, and lens phase profiles are commonly integrated within a single metasurface, enabling coordinated control over the OAM topological charge, ring radius, and propagation characteristics. In 2021, Zhou et al. demonstrated broadband perfect‐vortex‐beam generation in the visible regime using a single‐layer TiO_2_ dielectric PB‐phase metasurface [123]. As shown in Figure 4a, the target phase profile was constructed by superimposing helical, axicon, and lens phase terms, thereby combining OAM encoding and perfect‐vortex‐beam shaping within a single‐layer device. In the same year, Xie et al. employed a propagation‐phase dielectric metasurface to encode distinct phase profiles under orthogonal linearly polarized illumination, generating perfect vortex beams with different ring radii or topological charges and thereby enabling polarization‐controlled OAM‐field manipulation [124]. In 2024, Su et al. further proposed a metasurface‐based approach for generating perfect vortex beams with high topological charges, demonstrating that metasurface phase encoding can extend the accessible OAM range and broaden the scope of structured optical‐field manipulation [125]. Collectively, these studies show that integrating the OAM helical phase with axicon and lens phases, together with polarization‐channel engineering, enables metasurfaces to advance from conventional vortex‐beam generation toward perfect‐vortex‐beam shaping and multichannel OAM control.

**FIGURE 4:**
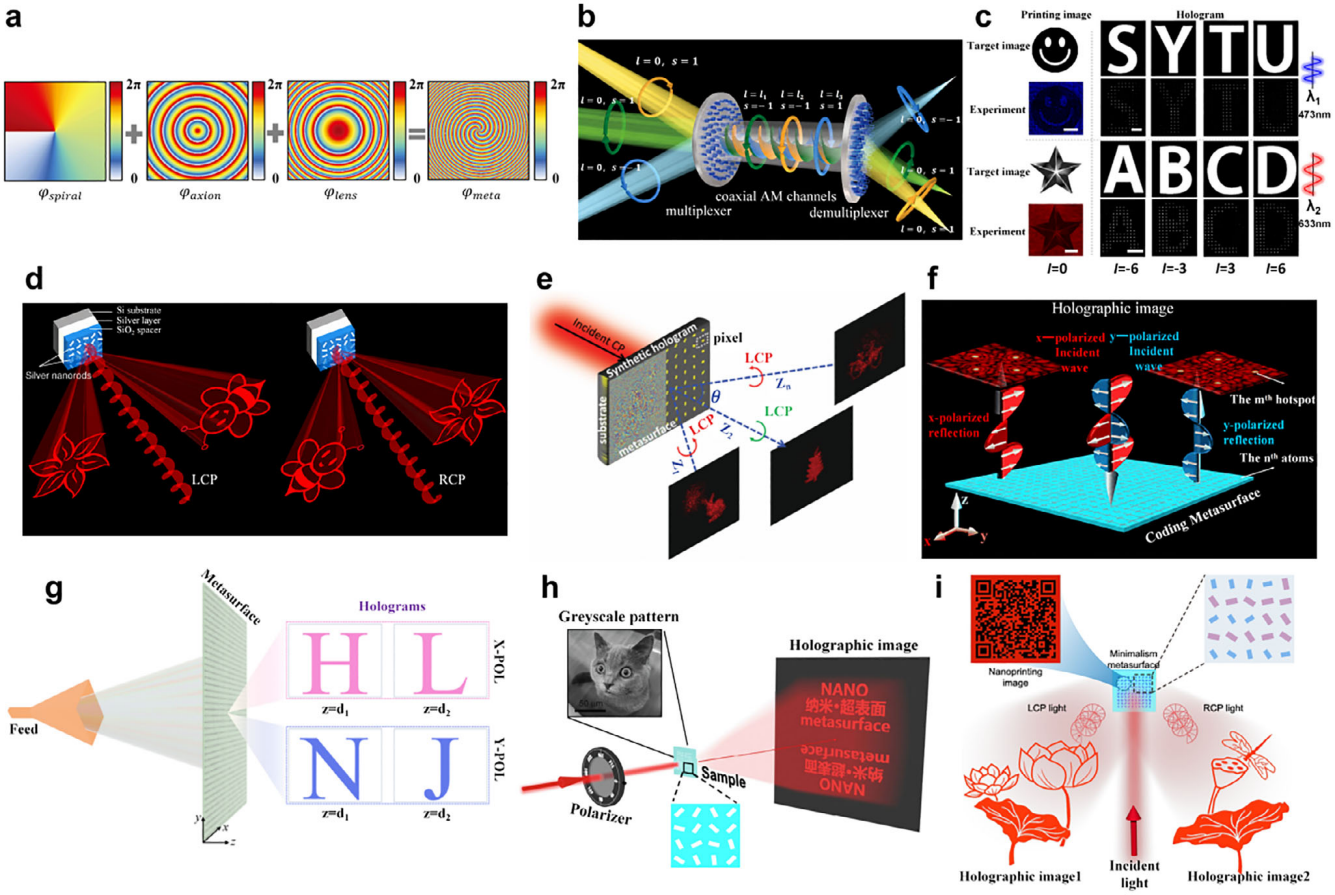
Joint control of phase, OAM, and spatial‐polarization degrees of freedom. (a) Phase superposition of spiral, axicon, and lens phases for perfect vortex beams. Reprinted with permission [123]. Copyright 2021, Wiley‐VCH GmbH. (b) Angular‐momentum multiplexing and demultiplexing with dielectric metasurfaces. Reprinted with permission [129]. Copyright 2020, WILEY‐VCH Verlag GmbH & Co. KGaA, Weinheim. (c) Multichannel printing and holographic reconstruction using OAM‐ and wavelength‐multiplexed metasurfaces. Reprinted under the terms of the CC BY 4.0 license [132]. Copyright 2024, The Authors. (d) Polarization‐selective image switching in helicity‐multiplexed holography. Reprinted under the terms of the CC BY 4.0 license [133]. Copyright 2015, The Authors. (e) Spatial‐polarization hybrid holography using PB‐phase metasurfaces. Reprinted with permission [134]. Copyright 2015, WILEY‐VCH Verlag GmbH & Co. KGaA, Weinheim. (f) Two independent holographic channels in a polarization‐multiplexed coding metasurface. Reprinted with permission [135]. Copyright 2021, AIP Publishing. (g) Dual‐plane holography under *x*/*y* polarization channels using Huygens metasurfaces. Reprinted with permission [137]. Copyright 2025, Optica Publishing Group. (h) Independent near‐field grayscale images and far‐field holography using Malus metasurfaces. Reprinted under the terms of the CC BY 4.0 license [138]. Copyright 2020, The Authors. (i) Minimalist multifunctional metasurface integrating near‐field nanoprinting and helicity‐multiplexed far‐field holography. Reprinted with permission [139]. Copyright 2024, Optica Publishing Group.

At the level of channel expansion, OAM can be jointly encoded with spin and spatial channels, allowing distinct polarization states or spatial regions to carry mutually independent vortex modes. For anisotropic PB‐phase metasurfaces, left‐ and right‐handed circularly polarized illumination generally acquire PB‐phases of opposite signs, making such platforms particularly suitable for spin‐selective OAM generation. In 2016, Guo et al. combined PB‐phase with plasmonic retardation phase in a continuous annular plasmonic metasurface, enabling flexible generation of OAM beams with both integer and fractional topological charges and thereby expanding the design freedom available for phase‐encoded OAM control [126]. In the same year, Mehmood et al. introduced a visible‐light metasurface for vortex‐beam structuring and spatial multiplexing [127]. By integrating lens and helical phase profiles within a single device and assigning distinct sublens designs to different spatial regions, the metasurface generated focused vortex beams with different topological charges at separate focal planes, thus realizing spatially multiplexed control of visible OAM fields. In 2017, Devlin et al. demonstrated a spin‐to‐orbital angular momentum converter based on a TiO_2_ dielectric metasurface [128]. By azimuthally varying the orientation of birefringent nanofins operating as local half‐wave plates, the device introduced a spin‐dependent PB‐phase, simultaneously reversed the handedness of circular polarization, and generated OAM vortex beams whose topological‐charge sign was determined by the incident spin state. The same platform further enabled the generation of high‐order and fractional OAM modes, as well as collinear vortex beams carrying two distinct topological charges. Collectively, these studies establish three complementary routes for expanding OAM channels: spin‐OAM mapping through PB‐phase, access to integer and fractional topological charges through hybrid‐phase encoding, and multiplexing of multiple OAM modes through spatial partitioning or interleaved arrangements. Metasurface‐based OAM control has therefore advanced from single‐topological‐charge generation toward spin‐addressable operation, spatial multiplexing, and multichannel structured‐light encoding.

For information transmission, a reversible mapping between input spatial channels and OAM modes must be established to enable OAM multiplexing and demultiplexing. Through off‐axis phase engineering, a single metasurface can convert fundamental‐mode beams incident from different directions into coaxially propagating OAM beams with distinct topological charges, thereby realizing OAM multiplexing. When operated in reverse, the same device can separate different OAM modes into distinct propagation directions for demultiplexing [82]. In 2020, Li et al. proposed a dielectric‐metasurface platform for angular‐momentum multiplexing and demultiplexing [129]. By combining off‐axis phase design with the photonic spin Hall effect, the device enabled compact multiplexing and demultiplexing of both OAM and spin angular momentum (SAM) channels. As shown in Figure 4b, multiple off‐axis input signals were converted into coaxial angular‐momentum channels, whereas during demultiplexing the different angular‐momentum states were focused onto designated positions in the target plane, facilitating integration with waveguides or photodetectors. In 2023, Wu et al. introduced a polarization‐dependent phase‐modulation metasurface that combined an extended PB‐phase with a Dammann vortex grating, enabling simultaneous multiplexing and demultiplexing of OAM modes and polarization channels and providing an integrated solution for OAM‐channel processing [130]. OAM control can be further combined with wavelength‐selective responses and complex‐amplitude encoding to expand the dimensionality of vortex‐field channels. In 2018, Jin et al. employed a single‐layer PB‐phase metasurface comprising two classes of wavelength‐selective nanoantennas that independently modulated the phase at different wavelengths, thereby generating wavelength‐dependent three‐dimensional arrays of volumetric optical vortices [131]. This work demonstrated that spectral channels can be used to tailor the spatial distribution of OAM vortex fields. In 2024, He et al. proposed a multiplexed OAM‐wavelength manipulation scheme based on arbitrary complex‐amplitude modulation, enabling independent printed or holographic information to be generated under different illumination wavelengths and incident OAM states [132]. As illustrated in Figure 4c, the metasurface produced distinct near‐field printed images at 473 and 633 nm and reconstructed independent far‐field holographic images under illumination by different incident OAM topological charges. These results demonstrate that phase encoding, amplitude modulation, and wavelength‐selective responses can be jointly exploited for multidimensional, multichannel control of OAM light fields.

Joint phase‐OAM control is evolving from simple helical‐phase encoding toward mode‐selective complex‐response engineering across polarization, wavelength, incidence‐angle, and spatial channels. Within this framework, OAM can serve not only as an output spatial mode generated by a metasurface, but also as an input channel for information addressing and multiplexing, while mode‐to‐space conversion enables subsequent demultiplexing. Future progress will therefore depend less on merely increasing the accessible topological‐charge range and more on simultaneously improving mode purity, channel independence, broadband operation, and dynamic reconfigurability. Equally important will be the co‐integration of OAM devices with waveguides, photodetectors, and on‐chip optical communication systems.

### 3.5 Joint Spatial‐Polarization Control

Spatial and polarization degrees of freedom constitute two key dimensions of multidimensional light‐field manipulation by metasurfaces. Spatial degrees of freedom encompass information encoded in transverse position, propagation direction, imaging depth, near‐ and far‐field regions, and spatial modes, whereas polarization describes the vectorial oscillation state of the electric field and can serve as an independent information channel or functional selector. The central principle of joint spatial‐polarization control is to associate a specific polarization state with a prescribed spatial optical response, thereby enabling a single metasurface to perform distinct wavefront transformations, image reconstructions, spatial‐multiplexing operations, or vectorial‐field generation under different input or output polarization conditions.

From the perspective of implementation, joint spatial‐polarization control typically exploits the polarization‐selective Jones‐matrix response of anisotropic meta‐atoms to map different input or output polarization states onto distinct spatial‐phase, amplitude, or imaging channels. At the level of polarization‐selective scalar holography, representative approaches have primarily employed circular or orthogonal linear polarization states as readout conditions, thereby associating different polarization channels with distinct spatial images or reconstruction positions. In 2015, Wen et al. demonstrated helicity‐multiplexed broadband metasurface holography by exploiting the spin dependence of the PB‐phase [133]. Under left‐ and right‐handed circularly polarized illumination, the metasurface imparted phase responses of opposite signs or complex‐conjugate forms, enabling polarization‐selective far‐field holographic reconstruction. As shown in Figure 4d, the device switched between the holographic images “flower” and “bee” under left‐circularly polarized (LCP) and right‐circularly polarized (RCP) illumination, respectively, demonstrating that circular polarization can serve as an effective degree of freedom for spatial‐information readout and image switching. In the same year, Huang et al. reported a broadband hybrid‐multiplexed PB‐phase metasurface holography scheme [134]. By leveraging the helicity dependence of the PB‐phase, they incorporated circular‐polarization state, reconstruction position, and observation angle into the selective retrieval of holographic information, enabling multiple images to be independently multiplexed within a single metasurface. As illustrated in Figure 4e, different images were associated with distinct combinations of circular polarization, reconstruction position, and off‐axis viewing angle, showing that polarization can be jointly encoded with spatial variables such as image position and observation direction for multichannel holographic multiplexing. In 2021, Shang et al. further introduced a polarization‐multiplexed coding‐metasurface holography approach in which independent phase profiles were encoded into two orthogonal linear‐polarization channels, enabling separate reconstruction of different holographic images [135]. As shown in Figure 4f, the coding metasurface reconstructed distinct near‐field holographic images under *x‐* and *y*‐polarized illumination, highlighting the ability of orthogonal linear‐polarization channels to independently address spatial image information. Collectively, these studies demonstrate that polarization states can function not only as switching and addressing conditions for holographic images, but also as components of multidimensional readout schemes when combined with spatial parameters such as reconstruction position and observation angle, thereby providing a foundation for increasing information capacity through joint spatial‐channel multiplexing.

The integration of spatial‐channel and polarization multiplexing represents a particularly prominent strategy for joint spatial‐polarization control. Conventional spatial‐channel multiplexing assigns different spatial regions or subchannels to distinct image information, enabling multiframe holographic display; however, its channel capacity, energy utilization, and crosstalk‐suppression capability remain limited. In 2021, Duan et al. proposed a polarization‐dependent dynamic holographic metasurface that combined spatial‐channel multiplexing with circular‐polarization multiplexing, allowing a metasurface containing *N* spatial channels to support as many as 3^*N*^ holographic frames [136]. Dynamic frame switching was achieved through different combinations of spatial channels, while left‐ and right‐handed circular polarizations further expanded the number of addressable holographic channels. This work demonstrated that the coordinated design of spatial and polarization channels can substantially increase the multiplexing dimensionality of metasurfaces. In 2025, Yang et al. introduced a spatial‐polarization‐multiplexed Huygens metasurface that combined two orthogonal linear‐polarization channels, multiplane near‐field reconstruction, and an improved weighted Gerchberg–Saxton algorithm to achieve high‐efficiency, low‐crosstalk multichannel holographic display [137]. As shown in Figure 4g, the device independently reconstructed distinct holographic images at two different imaging planes under *x‐* and *y*‐polarized illumination, respectively, thereby demonstrating the synergistic control of polarization channels and spatial‐depth multiplexing.

Beyond transverse spatial channels, the spatial degree of freedom can be further extended to near‐ and far‐field regions, longitudinal imaging depth, and three‐dimensional volumetric space. In 2020, Deng et al. exploited the orientational degeneracy inherent in Malus’ law to preserve continuous near‐field intensity modulation while encoding a PB‐phase through the selection of degenerate orientations [138]. Using metasurfaces composed of single‐sized meta‐atoms, they simultaneously realized grayscale near‐field nanoprinting and an independent far‐field holographic reconstruction. As shown in Figure 4h, near‐field intensity information and far‐field holographic phase were jointly encoded into the same set of anisotropic nanostructures, enabling synergistic multiplexing of near‐/far‐field spatial channels and polarization modulation. Near‐/far‐field multiplexing can also be combined with circular‐polarization selectivity. In 2024, Liang et al. introduced a structurally minimal multifunctional metasurface that employed only two types of nanostructures to realize continuous‐amplitude near‐field nanoprinting and helicity‐multiplexed far‐field holography [139]. As illustrated in Figure 4i, Malus‐law‐controlled near‐field nanoprinted images and LCP‐/RCP‐selective far‐field holographic images were integrated within a single metasurface, while the coordinated optimization of geometric and propagation phases enabled holographic‐image switching between the two circular‐polarization channels. This strategy reduced structural complexity while expanding the multiplexing capability across near‐/far‐field spatial responses and circular‐polarization channels. The spatial dimension can be extended further along the longitudinal depth axis. In 2026, Xiong et al. demonstrated polarization‐dependent three‐dimensional holography using gradient‐descent‐based inverse design and a high‐throughput nanoimprinting process based on TiO_2_‐particle‐doped resin, reconstructing multiple three‐dimensional images across different polarization channels and depth planes [140]. As shown in Figure 5a, distinct three‐dimensional holographic information was encoded into the LCP and RCP channels, establishing a coupled relationship between polarization state and imaging depth and thereby enabling polarization‐selective reconstruction of three‐dimensional optical fields. This work demonstrates that joint spatial‐polarization control can progress beyond two‐dimensional image multiplexing toward multidepth, multichannel encoding of volumetric light fields.

**FIGURE 5:**
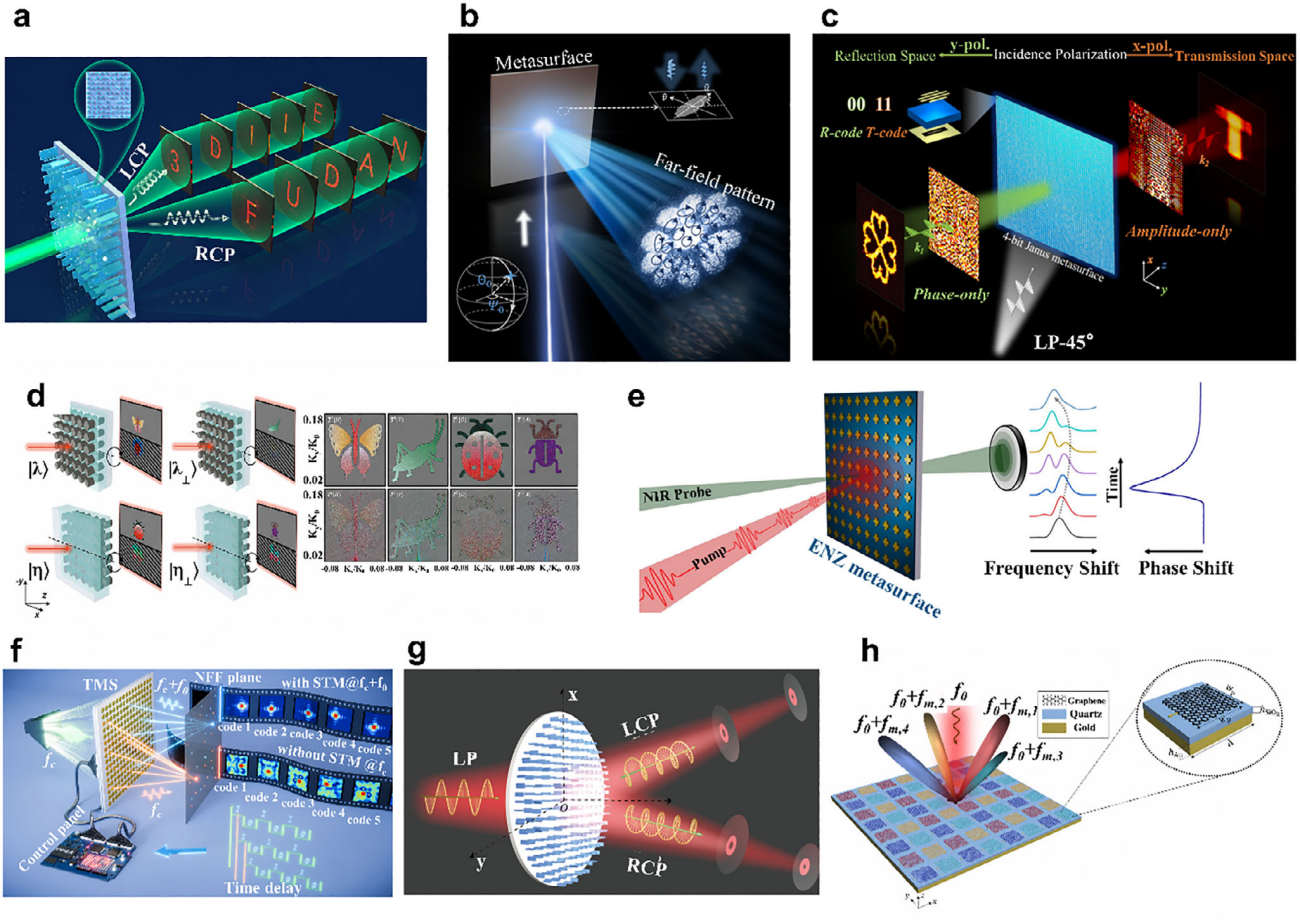
Temporal modulation and multidimensional multiplexing in metasurfaces. (a) Multidepth three‐dimensional (3D) holography under left‐circularly polarized (LCP) and right‐circularly polarized (RCP) channels. Reprinted under the terms of the CC BY 4.0 license [140]. Copyright 2025, The Authors. Advanced Science published by Wiley‐VCH GmbH. (b) Coordinated control of spatial intensity and local polarization in efficient vector holography. Reprinted under the terms of the CC BY 4.0 license [143]. Copyright 2025, The Authors. Published by SPIE and CLP. (c) Full‐space spatial‐polarization‐multiplexed holography enabled by a 4‐bit millimeter‐wave Janus metasurface. Reprinted with permission [148]. Copyright 2024, Optica Publishing Group. (d) Polarization‐direction‐multiplexed vector holography using bilayer Janus metasurfaces. Reprinted with permission [149]. Copyright 2024, The Authors. Advanced Materials published by Wiley‐VCH GmbH. (e) Ultrafast frequency shift and nonlinear phase modulation in time‐varying epsilon‐near‐zero (ENZ) metasurfaces. Reprinted under the terms of the CC BY 4.0 license [159]. Copyright 2026, The Authors. Advanced Optical Materials published by Wiley‐VCH GmbH. (f) Harmonic near‐field focusing using space‐time‐modulated metasurfaces. Reprinted under the terms of the CC BY 4.0 license [163]. Copyright 2025, The Authors. Laser & Photonics Reviews published by Wiley‐VCH GmbH. (g) Spin‐multiplexed dielectric metasurfaces for multidimensional focused vortex beams. Reprinted under the terms of the CC BY 4.0 license [168]. Copyright 2022, The Authors. (h) Multifrequency multibeam scanning using space‐time shared‐aperture metasurfaces. Reprinted under the terms of the CC BY 4.0 license [171]. Copyright 2020, The Authors.

At the level of vectorial light‐field manipulation, joint spatial‐polarization manipulation has progressed beyond image switching across discrete polarization channels toward the coordinated design of multiple input‐output polarization pathways, spatial intensity distributions, and local polarization states. In 2018, Zhao et al. introduced a multichannel vectorial holography scheme in which the cross‐sectional dimensions and orientation angles of birefringent dielectric nanofins were simultaneously engineered to achieve coordinated control of polarization‐dependent phase and Jones‐matrix responses [141]. Distinct input‐output polarization combinations were thereby mapped onto corresponding holographic phase profiles. The device established 12 readout channels based on linear and circular polarization states, enabling the reconstruction of three primary holographic images and multiple composite patterns. Different image components were simultaneously assigned distinct output‐polarization properties, thereby realizing multichannel vectorial holographic display and polarization‐encrypted information encoding. Building on this framework, vectorial holography evolved from image combination across discrete polarization channels toward continuous and coordinated control of spatial intensity and local polarization distributions. In 2021, Song et al. proposed a vectorial Fourier metasurface that employed an improved iterative Fourier‐transform algorithm to achieve broadband and decoupled control over the far‐field intensity profile, local polarization azimuth, and ellipticity distribution [142]. This approach enabled polarization information to be independently embedded within a prescribed spatial‐intensity pattern, facilitating the generation of complex vectorial light fields and highlighting its potential for polarization‐based information encryption. In 2025, Liu et al. further developed a high‐efficiency ultrathin vectorial holographic metasurface by combining the Gerchberg–Saxton algorithm with a wave‐decomposition method, thereby establishing a generalized design strategy applicable to arbitrarily prescribed incident polarization states [143]. As shown in Figure 5b, the metasurface comprised an array of a single meta‐atom type that simultaneously provided reflection‐phase modulation and polarization conversion. It reconstructed far‐field vectorial holographic images satisfying both the target intensity distribution and the prescribed local polarization pattern, thereby improving the efficiency, spatial resolution, and design generality of coordinated intensity‐polarization modulation.

Beyond holographic image reconstruction, joint spatial‐polarization control can also be extended to non‐imaging functionalities, including polarization‐dependent focusing, spatial‐mode conversion, and longitudinal light‐field evolution. By independently encoding focusing, helical, or longitudinal‐evolution phase profiles into different polarization channels, metasurfaces can establish programmable mappings between the incident polarization state and focal length, OAM mode, or propagation dynamics. This capability enables switchable multifocal operation, vector‐vortex‐beam generation, and polarization‐selective evolution of structured light fields [144, 145, 146]. These studies demonstrate that polarization can serve not only as a readout condition for holographic images, but also as a selective addressing degree of freedom for spatial modes and propagation behavior.

The spatial degree of freedom can be further extended to the transmission and reflection half‐spaces, as well as to forward and backward incidence directions. Janus metasurfaces exploit direction‐dependent responses under different propagation directions or output half‐spaces, providing an effective platform for overcoming the limitations of conventional single‐sided control and expanding the available channels for full‐space wavefront manipulation. In 2021, Shang et al. proposed an integrated transmission‐reflection Janus metasurface that reconstructed four independent holographic images under forward and backward y‐polarized illumination by selectively employing the co‐polarized reflection and cross‐polarized transmission channels [147]. This architecture enabled direction‐selective joint multiplexing of output space and polarization channels. In 2024, Zhang et al. introduced a single‐layer 4‐bit millimeter‐wave Janus metasurface that independently controlled the reflection phase and transmission amplitude in two orthogonal polarization channels [148]. Two distinct holographic images were reconstructed in the reflection and transmission half‐spaces, thereby realizing spatial‐polarization‐multiplexed holography, as shown in Figure 5c. In the same year, Kim et al. developed a bilayer Janus metasurface in which the front‐ and back‐side incidence directions and input polarization states served as joint readout conditions [149]. The device enabled bidirectional reconstruction of four independent vectorial holographic images, demonstrating coordinated control over propagation direction, spatial image content, and local polarization distribution, as illustrated in Figure 5d. In 2025, Wu et al. further incorporated polarization multiplexing into one transmission domain and two reflection domains, achieving six‐channel full‐space holographic display [150]. Collectively, these studies show that Janus and integrated transmission‐reflection metasurfaces can establish multiple mappings among polarization state, incidence direction, output half‐space, and propagation domain. Joint spatial‐polarization control is therefore progressing from single‐sided wavefront multiplexing toward bidirectional, multidomain, and high‐capacity full‐space information encoding.

Joint spatial‐polarization control has evolved from polarization‐selective image switching toward multiplane imaging, spatial‐mode manipulation, and full‐space wavefront control. Future advances will require increasing channel capacity while suppressing inter‐channel crosstalk, together with a balanced optimization of polarization purity, spatial resolution, and energy efficiency. These developments will further drive multichannel metasurfaces toward dynamic reconfigurability and on‐chip integration.

### 3.6 Joint Temporal‐Phase Control

Conventional phase‐only metasurfaces establish prescribed spatial phase profiles by tailoring the geometrical dimensions, orientation angles, or resonant properties of their constituent meta‐atoms, thereby enabling beam steering, focusing, holographic imaging, and structured‐light generation. Once fabricated, however, their phase responses are typically fixed, limiting their adaptability to applications such as dynamic beam scanning, reconfigurable communications, and real‐time information processing. To overcome this constraint, the temporal degree of freedom has been incorporated into metasurface design, extending the local phase from a static spatial function,Φ(*x*, *y*), to a spatiotemporal function,Φ(*x*, *y*, *t*). This extension enables dynamic switching among wavefront functionalities and further supports frequency conversion, harmonic manipulation, nonreciprocal propagation, and spatiotemporal coding, thereby driving the transition from static spatial‐phase engineering toward dynamic spatiotemporal wavefront control.

From a physical perspective, joint temporal‐phase control relies on dynamically modifying the effective electromagnetic response of individual meta‐atoms, such that the local phase imparted by the metasurface evolves in time. When metasurface elements at different spatial positions switch among prescribed phase states according to programmed temporal sequences, the output wavefront can be reconfigured in real time or repeatedly without refabricating the device. This capability supports dynamic beam steering, focal‐position scanning, and holographic‐image switching. When the phase modulation is periodic, the interaction between the incident wave and the time‐varying interface involves an exchange of energy, generating frequency sidebands or harmonic components. The metasurface can therefore manipulate spatial momentum and optical frequency simultaneously. Under the *e* ^−*i*ω*t*^ time‐harmonic convention, the local time‐varying complex scattering coefficient can be expanded as [151, 152]:

(17) $$\begin{array}{c} s\left(x,y,t\right)=\sum_{n=-\infty }^{+\infty }S_{n}\left(x,y\right)e^{-in\Omega t} \end{array}$$

where *s*(*x*, *y*, *t*) denotes the local complex reflection or transmission coefficient, *t* is time, Ω is the angular modulation frequency, *n* denotes the temporal harmonic order, and *S_n_* (*x*,*y*) is the complex scattering coefficient associated with the *n*‐th temporal harmonic. The magnitude |*S_n_* (*x*,*y*)| determines the conversion amplitude of the corresponding harmonic component, while *arg*[*S_n_* (*x*,*y*)] defines its spatial phase distribution. For monochromatic illumination at angular frequency ω_0_, the output field contains frequency components at:

(18) $$\begin{array}{c} \;\omega_{n}=\omega_{0}+n\Omega \end{array}$$

where ω_*n*_ denotes the angular frequency of the *n*‐th output frequency component. When the modulation sequence incorporates a spatially varying time delay τ(*x*, *y*), such that *s*(*x*, *y*, *t*)  =  *s*_0_ [*t*  −  τ(*x*, *y*)], with *s*_0_ denoting the unshifted reference temporal scattering waveform, the *n*‐th harmonic acquires an additional phase Φ_*n*_(*x*,*y*):

(19) $$\begin{array}{c} \Phi_{n}\left(x,y\right)=n\Omega \tau \left(x,y\right) \end{array}$$

with the overall sign depending on the adopted definitions of the time‐harmonic convention and temporal delay. A spatial distribution of time delays can therefore be mapped onto a harmonic‐order‐dependent phase profile, allowing temporal coding to perform frequency selection and wavefront shaping simultaneously. Studies of nonreciprocal time‐varying interfaces, temporally modulated graphene meta‐atoms, time‐domain digital coding, and space‐time coding have demonstrated that temporal modulation can extend metasurface control beyond static spatial wavefronts to encompass frequency order, harmonic amplitude and phase, and propagation momentum [152, 153, 154, 155]. Joint temporal‐phase control therefore involves not only the dynamic switching of static phase profiles, but also the direct formation of frequency‐converted fields and spatiotemporally structured wavefronts.

For dynamic phase modulation, tunable materials and integrated electronic circuits provide two representative routes for modifying the local phase response of metasurfaces after fabrication. In 2020, Venkatesh et al. experimentally demonstrated a high‐speed programmable terahertz holographic metasurface assembled from tiled complementary metal‐oxide‐semiconductor (CMOS) chips [156]. The fabricated device comprised 576 individually addressable meta‐atoms with 8‐bit digital control and gigahertz‐speed reconfiguration, enabling programmable joint phase‐amplitude control, dynamic beamforming across ±30°, multibeam generation, and programmable holographic reconstruction at 0.3 THz. In 2021, Wang et al. proposed a doubly resonant graphene–metal hybrid metasurface for full 360° dynamic phase modulation. Numerical simulations predicted a phase coverage of 361° at 1.05 THz while maintaining a reflectance above 20% and further demonstrated dynamically tunable beam steering and focusing [157]. These studies represent two complementary approaches to dynamic reconfigurability: high‐speed digital programming through integrated electronic circuits and continuous electrical tuning of graphene. In their demonstrated or proposed operating modes, these approaches primarily realize spatial wavefront shaping by discretely or continuously tuning the local phase response, without explicitly exploiting harmonic frequency conversion. By contrast, time‐periodic modulation, as a form of spatiotemporal modulation, can mediate energy exchange with the incident wave and generate frequency‐converted harmonics, thereby extending phase control to frequency‐converted wavefront shaping within the broader framework of spatiotemporal modulation.

When temporal modulation directly mediates frequency conversion and harmonic generation, a metasurface no longer serves solely as a dynamically reconfigurable wavefront element; rather, it can simultaneously control spatial momentum and spectral content, thereby functioning as a platform for joint space‐time manipulation. In 2018, Salary et al. introduced a harmonic wavefront‐engineering framework based on time‐varying metasurfaces [151]. By electrically programming the temporal phase delays of individual elements, they imparted controllable, harmonic‐order‐dependent spatial phase profiles to the frequency‐converted sidebands. In 2024, Amra et al. developed a theory of linear frequency conversion in time‐varying metasurfaces, showing that external temporal modulation can enable parametric frequency conversion within a linear‐response framework [158]. This mechanism can emulate the frequency‐mixing functionality of nonlinear optical devices without relying on intrinsic material nonlinearities. Beyond the discrete harmonics generated by periodic modulation, ultrafast transient changes in the optical medium can also induce continuous or broadband frequency shifts through temporal refraction or cross‐phase modulation. In 2026, Dhama et al. demonstrated tunable broadband frequency translation using a time‐varying epsilon‐near‐zero (ENZ) metasurface, in which rapid pump‐induced refractive‐index changes produced cross‐phase modulation [159]. As shown in Figure 5e, the ultrafast pump‐probe interaction induced both a frequency shift and a nonlinear phase change in the probe field as it propagated through the time‐varying ENZ metasurface, thereby enabling ultrafast phase modulation. Collectively, these studies demonstrate that time‐varying phase modulation can directly connect frequency conversion and harmonic generation with wavefront engineering, providing a critical foundation for the evolution of metasurfaces from static spatial‐phase control toward dynamic spatiotemporal manipulation.

For nonreciprocal propagation and direction‐selective control, time‐varying phase modulation can be co‐designed with a static spatial phase gradient or a traveling‐wave spatiotemporal modulation. By introducing directional biases in both frequency and in‐plane momentum, the forward and backward propagation processes can be made to satisfy distinct frequency‐momentum matching conditions, thereby enabling coordinated control over frequency, in‐plane wave‐vector, and propagation direction. In 2019, Guo et al. proposed a nonreciprocal metasurface based on space‐time phase modulation [160]. By superimposing a static spatial phase gradient and a traveling‐wave spatiotemporal phase modulation in a nonlinear metasurface, they realized nonreciprocal reflection in the near‐infrared regime, accompanied by unidirectional transitions in both energy and in‐plane momentum. In the same year, Zang et al. introduced a time‐modulated gradient metasurface in which the phase delays of low‐frequency modulation signals applied to individual unit cells were engineered to impart controllable spatial phase gradients to frequency‐converted harmonic fields, thereby enabling nonreciprocal wavefront engineering [161]. By exploiting the opposite signs of the phase‐gradient responses associated with up‐ and down‐conversion, this strategy enabled nonreciprocal beam steering and focusing. The former approach emphasizes direction‐dependent frequency‐momentum conversion arising from the combined action of a static spatial phase gradient and traveling‐wave temporal modulation, whereas the latter exploits the difference between the phase gradients associated with distinct frequency‐conversion pathways to achieve nonreciprocal wavefront control. Collectively, these studies demonstrate that temporal modulation can establish direction‐dependent coupling among spatial phase, frequency conversion, and propagation direction. Time‐varying metasurfaces can therefore not only dynamically reconfigure wavefronts, but also reshape the frequency‐conversion channels and wavefront responses associated with forward and backward propagation, offering a promising route toward magnet‐free nonreciprocal devices and direction‐selective wavefront manipulation.

At the system level, joint temporal‐phase control is progressively extending to array‐based platforms, including programmable metasurfaces and reconfigurable intelligent surfaces (RISs). This evolution is shifting the scale of control from dynamically tunable responses of individual meta‐atoms toward array‐level synthesis of spatiotemporal wavefronts. In 2024, Zhao et al. developed a 2‐bit RIS prototype featuring four discrete phase states and rapid, independent control of the entire array [162]. The system enabled reconfigurable beamforming, three‐dimensional pencil‐beam scanning and tracking, and dynamic manipulation of vortex beams. In 2025, Wang et al. incorporated spatiotemporal modulation into near‐field focusing and established quasi‐continuous phase compensation at the *k*  =   + 1 harmonic, thereby reducing the required number of meta‐atoms while preserving favorable focusing performance [163]. As shown in Figure 5f, programmed time delays provided additional phase compensation for the target harmonic, improving near‐field focusing quality without altering the physical structure of the metasurface. Collectively, these studies demonstrate that introducing the temporal dimension extends phase modulation from element‐level dynamic responses to array‐level spatiotemporal coding, thereby enabling system‐scale functionalities such as dynamic beamforming and harmonic near‐field focusing.

### 3.7 Three‐Dimensional Joint Control

As metasurface design capabilities continue to advance, control schemes based on two coupled degrees of freedom are increasingly evolving toward the coordinated manipulation of three or more dimensions, driven by the demands of high‐capacity information multiplexing, complex structured‐light generation, and system‐level multifunctional integration. Such multidimensional manipulation requires not only the simultaneous participation of multiple electromagnetic degrees of freedom in constructing the target field, but also careful management of channel independence, inter‐channel crosstalk, and energy allocation. This expanded design framework enables a single metasurface to support substantially more complex electromagnetic responses, information‐encoding architectures, and multiplexed functionalities.

Joint phase‐amplitude‐polarization control typically employs polarization states as selection or multiplexing channels, while coordinately encoding the complex‐amplitude responses of different polarization components. This approach enables the integrated design of wavefronts, intensity distributions, and polarization‐dependent responses. In 2023, Sun et al. introduced a metasurface‐based complex‐amplitude modulation scheme based on full‐phase modulation [164]. By synergistically engineering propagation and PB‐phases, they independently tailored the complex amplitudes of the co‐ and cross‐polarized components under circularly polarized illumination and applied this capability to multifocal focusing and the generation of focused vortex fields. In the same year, Zhang et al. employed a metasurface coupler to achieve full‐range complex‐amplitude modulation of excited surface waves through circular‐polarization‐conversion channels, thereby realizing Airy surface‐wave generation and steering, as well as bifocal focusing [165]. This work extended complex‐amplitude modulation from free‐space waves to surface‐wave platforms. Building on these advances, Li et al. proposed a multiplexed interference metasurface in 2026 [166]. In this design, the PB‐phase encoded the target phase, interference among the diffraction fields of sub‐supercells regulated the amplitude, and sub‐supercells addressing the left‐ and right‐handed circular‐polarization channels were spatially interleaved. This architecture enabled complete control over phase, amplitude, and polarization within prescribed diffraction channels. Collectively, these studies demonstrate that integrating complex‐amplitude engineering, polarization‐channel multiplexing, and sub‐supercell interference can extend polarization‐resolved complex‐amplitude encoding toward complete phase‐amplitude‐polarization control in selected diffraction channels, thereby enhancing the capacity for multidimensional electromagnetic‐field encoding and multifunctional integration.

In joint polarization‐OAM‐spatial control, polarization or spin states typically serve as input‐selection and channel‐multiplexing degrees of freedom, OAM topological charge provides the modal basis for structured light, and propagation distance, focal plane, or beam direction is further exploited to define spatial channels. This framework enables coordinated control over multidimensional structured light fields. In 2019, Zhang et al. designed the PB‐phase distribution of a plasmonic metasurface using caustic theory [167]. By superimposing off‐axis vortex components with distinct circular‐polarization states and helical phase charges, they programmed the evolution of the intensity, polarization distribution, and OAM topological charge of vector vortex beams along the propagation direction, thereby realizing joint control over polarization, OAM, and longitudinal propagation space. In 2022, Liu et al. proposed an all‐dielectric spin‐multiplexed metasurface that combined propagation and PB‐phases to theoretically and numerically generate spin‐dependent focused vortex beams with distinct topological charges, focal lengths, and spatial positions under left‐ and right‐handed circularly polarized illumination [168]. As shown in Figure 5g, under linearly polarized incidence, the two orthogonal circularly polarized vortex components further interfered to form vector vortex beams with different modal structures, thereby establishing a programmable mapping among input spin, OAM mode, and spatial focusing channel. In 2023, Li et al. introduced a polarization‐dependent transmissive digital coding metasurface in the microwave regime [169]. By independently implementing 3‐bit phase coding in two orthogonal linear‐polarization channels, the device generated four OAM beams carrying two distinct topological charges along different prescribed directions, thus realizing joint multiplexing of polarization channels, OAM modes, and transverse spatial beam channels. In 2025, Ma et al. further developed a metasurface composed of interleaved quarter‐wave‐plate‐like meta‐atoms, in which the helical and hyperbolic focusing phases were encoded through the propagation and PB‐phases, respectively [170]. Theoretical and numerical analyses demonstrated the selective generation of circularly polarized vortex beams, linearly polarized vortex beams, and vector beams across five longitudinal focal planes, extending joint polarization‐OAM control into the longitudinal multifocal domain. Collectively, these studies show that polarization states and OAM modes can be jointly mapped onto spatial channels defined by propagation distance, beam direction, and focal plane. Metasurface control of structured light has therefore evolved from single‐plane, single‐mode generation toward continuously evolving longitudinal fields, transversely multiplexed multibeam architectures, and longitudinal multifocal addressing.

In joint space‐time‐frequency control, spatial coding defines the wavefront and propagation direction associated with each frequency channel, temporal modulation governs the frequency‐conversion process and harmonic power distribution, and the resulting frequency components serve as independent channels for carrying beams or information. In 2018, Zhang et al. introduced a space‐time‐coding digital metasurface in which periodically switched spatial coding sequences to integrate beam‐direction control with harmonic‐spectrum modulation on a single platform, thereby enabling coordinated design across spatial, temporal, and frequency channels [152]. In 2020, Barati Sedeh et al. proposed a space‐time shared‐aperture metasurface that partitioned a single aperture into multiple interleaved modulated subarrays [171]. By assigning distinct modulation frequencies and progressive modulation‐phase delays to different subarrays, the device achieved mutually independent beam scanning across multiple harmonic channels. As shown in Figure 5h, the interleaved subarrays driven at different modulation frequencies formed orthogonal frequency channels, while the spatially distributed modulation‐phase delays determined the beam direction of each channel. In 2023, Wang et al. further introduced a multi‐partition asynchronous space‐time‐coding digital metasurface [172]. By independently programming the modulation frequency, temporal waveform, and spatial phase distribution of each partition, the approach reduced the coupling among harmonic channels inherent to conventional space‐time coding and enabled independent control over the amplitude, phase, and scattering direction of multiple frequency components. The platform further demonstrated multiplexed information transmission using eight carrier frequencies. In 2025, Wu et al. proposed and experimentally demonstrated a space‐time holographic metasurface antenna capable of jointly encoding and reconstructing the amplitude, phase, and frequency information of object waves [173]. By combining spatiotemporal modulation with heterodyne holography, the platform realized frequency conversion and holographic beamforming in the far field, together with multifrequency two‐dimensional and three‐dimensional holographic imaging in the near field. Collectively, these studies show that joint space‐time‐frequency control has progressed from the simultaneous regulation of beam direction and harmonic power toward independent multifrequency multibeam manipulation, frequency‐channel decoupling, and full‐field multifrequency holographic reconstruction.

Joint control over three degrees of freedom extends metasurface functionality beyond pairwise coupling toward coordinated mappings among local complex responses, structured‐light modes, and spatiotemporal‐spectral channels. Future progress will require greater independent controllability of each degree of freedom and more efficient energy utilization, together with effective suppression of multichannel crosstalk. Equally important will be reducing the computational burden of multiobjective joint optimization and the hardware complexity associated with large‐scale driving and control. The implementation approaches, degree‐of‐freedom independence, representative functions, advantages, and remaining challenges of these joint‐control strategies are compared in Table 2.

**TABLE 2:** Comparison of joint control strategies for metasurface‐based multidimensional light‐field manipulation.

Joint degrees of freedom	Core implementation methods	Decoupling/independence	Representative functions	Major advantages	Key challenges	Refs.
Phase–amplitude	Co‐design of geometrical dimensions and orientation angles; decoupling through birefringence and Jones matrix design^a^; multipolar resonance and PB‐phase control	Independent or near‐independent control of amplitude and phase; some schemes can be extended to multichannel complex‐amplitude encoding	Complex‐amplitude holography, structured light generation, and multifunctional wavefront control	Improved light‐field reconstruction fidelity and flexible energy allocation	Residual amplitude‐phase coupling, limitations in efficiency and operating bandwidth, and structural and design complexity	[85, 86, 87, 88, 92]
Phase–polarization	PB‐phase and propagation‐phase engineering, together with Jones matrix design	Relatively independent control of spin or orthogonal polarization channels	Polarization‐multiplexed holography, spin‐selective focusing, and functional switching	High channel capacity and enhanced functional integration	Polarization‐conversion efficiency, inter‐channel crosstalk, and operating bandwidth	[20, 98, 105, 106]
Phase–wavelength^c^	Dispersion engineering, group delay compensation, and wavelength‐selective phase encoding	Continuous‐band responses require coordinated design, whereas discrete wavelength channels can be selectively or relatively independently engineered	Achromatic focusing, multiwavelength holography, and wavelength multiplexing	Integration of broadband achromaticity and wavelength multiplexing	Limited group delay range; trade‐offs among efficiency, operating bandwidth, numerical aperture, and device aperture; inter‐channel crosstalk	[108, 109, 111, 114]
Phase–OAM	Superposition of helical phase with focusing phase, beam‐steering phase, or axicon phase, combined with PB‐phase and off‐axis phase encoding	Relatively independent multiplexing based on the orthogonality of OAM modes with different topological charges	Vortex beam generation and focusing, perfect vortex beam shaping, and OAM multiplexing and demultiplexing	High‐dimensional mode space and increased channel capacity	OAM mode purity, inter‐channel crosstalk, and propagation stability	[82, 120, 123, 129, 132]
Spatial–polarization	Polarization‐selective Jones matrix design, spatial and depth multiplexing, near‐/far‐field encoding, and Janus full‐space control^b^	Relatively independent encoding across spatial‐polarization channels and input‐output polarization pathways	Polarization‐multiplexed and multichannel vectorial holography, near‐/far‐field imaging, three‐dimensional holography, and bidirectional full‐space display	High spatial information density, diverse readout conditions, and increased information capacity	Limited field of view, angular/depth‐dependent inter‐channel crosstalk, and trade‐offs among polarization purity, efficiency, and channel uniformity	[134, 138, 140, 141, 149]
Temporal–phase	Time‐varying meta‐atoms, digital coding, and spatiotemporal phase modulation	Dynamically programmable local phase, with selective control of harmonic amplitude, phase, and propagation direction	Dynamic beam scanning and focusing, frequency conversion, harmonic wavefront engineering, and nonreciprocal propagation	Dynamic reconfigurability and coordinated spatial‐frequency control	Response speed, modulation depth, power consumption, frequency conversion efficiency, and undesired harmonic leakage	[151, 152, 157, 159, 160]
Phase–amplitude–polarization	Coordinated propagation‐phase and PB‐phase engineering, polarization‐channel multiplexing, and sub‐supercell interference control	Independent complex‐amplitude modulation within polarization channels, extendable to joint phase‐amplitude‐polarization control in selected diffraction channels	Multifocal and vortex beam focusing, Airy surface‐wave generation, and joint phase‐amplitude‐polarization control	Coordinated encoding of wavefront, intensity, and polarization responses with high functional integration	Residual amplitude‐phase coupling, polarization‐channel crosstalk, energy utilization efficiency, and sub‐supercell coupling	[164, 165, 166]
Polarization–OAM–space^d^	Polarization/spin‐channel multiplexing combined with PB‐phase, propagation‐phase, helical phase, and focusing phase encoding	Relatively independent encoding of polarization‐OAM channels, with spatial separation according to propagation distance, beam direction, or focal plane	Longitudinal evolution of vector vortex beams, spin‐selective focused vortex beams, transverse multichannel OAM beams, and longitudinal multifocal structured light generation	Joint control of internal structured light modes and external spatial channels, enabling increased mode dimensionality and multiplexing capacity	Polarization purity, OAM mode purity, transverse/longitudinal inter‐channel crosstalk, energy allocation, and conversion efficiency	[167, 168, 169, 170]
Space–time–frequency^e^	Spatial coding, periodic/aperiodic temporal modulation, shared‐aperture architectures, and asynchronous zonal control	Reduced coupling among harmonic channels, enabling relatively independent addressing and selective control of multiple frequency channels	Harmonic and multifrequency beam scanning, multicarrier communication, spectral and radiation pattern reconstruction, radar cross‐section control, and false target generation	Dynamic programmability, multifrequency multibeam multiplexing, and system‐level information transmission	Response speed, parasitic harmonics and undesired spectral leakage, inter‐channel coupling, conversion efficiency, and synchronization and drive complexity	[152, 171, 172, 173]

^a^ The Jones matrix describes the complex‐amplitude mapping between input and output polarization components.

^b^ Janus control refers to differentiated electromagnetic responses or functionalities under illumination from opposite propagation directions or on different spatial sides.

^c^ In phase‐wavelength control, wavelength denotes spectral channels associated with different incident or operating wavelengths.

^d^ In polarization–OAM–space control, space denotes spatial channels defined by propagation distance, beam direction, or focal plane.

^e^ In space–time–frequency control, frequency mainly denotes harmonic, sideband, or carrier‐frequency channels generated or addressed through temporal modulation, whereas modulation frequency refers to the drive frequency governing the temporal variation of the metasurface response.

## 4 Applications of Metasurface‐Based Multidimensional Light‐Field Manipulation

### 4.1 Optical Information Processing and Computing

Optical information processing and computing provide a natural application framework for metasurface‐based multidimensional light‐field manipulation, because multiple degrees of freedom of light can be directly encoded into parallel information channels and transformed without relying entirely on electronic post‐processing. As demands for greater information capacity, higher processing speed, greater system‐level integration, and enhanced security continue to grow, conventional architectures based on bulk optical components or electronic post‐processing are increasingly constrained by device footprint, power consumption, limited parallelism, and processing latency. Metasurfaces address these limitations by performing compact and parallel operations across phase, amplitude, polarization, wavelength, and spatiotemporal modes, thereby enabling multidimensional information encoding and multiplexing, optical encryption, and optical computing.

For multidimensional information encoding and multiplexing, metasurfaces can map distinct information streams onto mutually independent optical degrees of freedom, thereby overcoming the capacity limitations associated with encoding in a single intensity, phase, or spatial‐pattern channel. Conventional single‐channel metasurface holograms typically reconstruct only a limited number of images under fixed operating conditions, whereas multidimensionally multiplexed metasurfaces exploit degrees of freedom such as frequency and polarization to produce distinct information outputs under different optical excitation conditions. In 2023, Zhang et al. introduced a four‐channel metasurface‐holography scheme for joint polarization‐frequency‐multiplexed encryption [174]. By simultaneously exploiting the frequency and polarization degrees of freedom, the number of information channels was increased multiplicatively relative to schemes based on a single multiplexing dimension. The device reconstructed independent holographic images under different combinations of frequency and polarization, highlighting the advantages of multidimensional multiplexing for enhancing information capacity and enabling encrypted readout. In 2024, Zhu et al. further demonstrated a single‐layer frequency‐multiplexed PB‐phase metasurface that generated independent holographic images at four distinct operating frequencies, confirming that frequency can serve as an effective information channel for multichannel imaging and optical encryption [175].

Beyond frequency and polarization multiplexing, spatial degrees of freedom—including incidence angle, propagation direction, and reflection/transmission channels—can also serve as independent information dimensions in metasurface holography. In 2021, Jang et al. introduced angle‐multiplexed metasurface holography, in which independent wavefront responses under different incidence angles enabled four distinct holographic images to be recorded and reconstructed using a single metasurface [176]. This approach provided a route toward multichannel information storage and angle‐selective readout. In 2022, Liu et al. developed a high‐fidelity multiplexed metasurface hologram operating in the microwave regime [177]. By jointly exploiting frequency, circular‐polarization state, and propagation direction, the device supported four independent holographic‐image channels within a single metasurface. The combined amplitude‐phase design suppressed inter‐channel crosstalk and improved reconstruction fidelity, demonstrating its potential for information display, data storage, and multichannel encrypted readout. In 2025, Zhang et al. further proposed a multidimensionally multiplexed PB‐phase metasurface holography scheme [178]. Using a fully modulated dielectric PB‐phase metasurface, they achieved simultaneous and independent control over spatial amplitude, phase, and polarization, and applied this capability to multichannel holographic information multiplexing. As shown in Figure 6a, polarization state, propagation direction, and image content were treated as independent channels, enabling on‐demand retrieval of multiple holographic images. The platform further exhibited broadband operation, high information capacity, low crosstalk, and relatively uniform energy allocation, offering a promising architecture for information broadcasting, optical anticounterfeiting, and secure information encryption.

**FIGURE 6:**
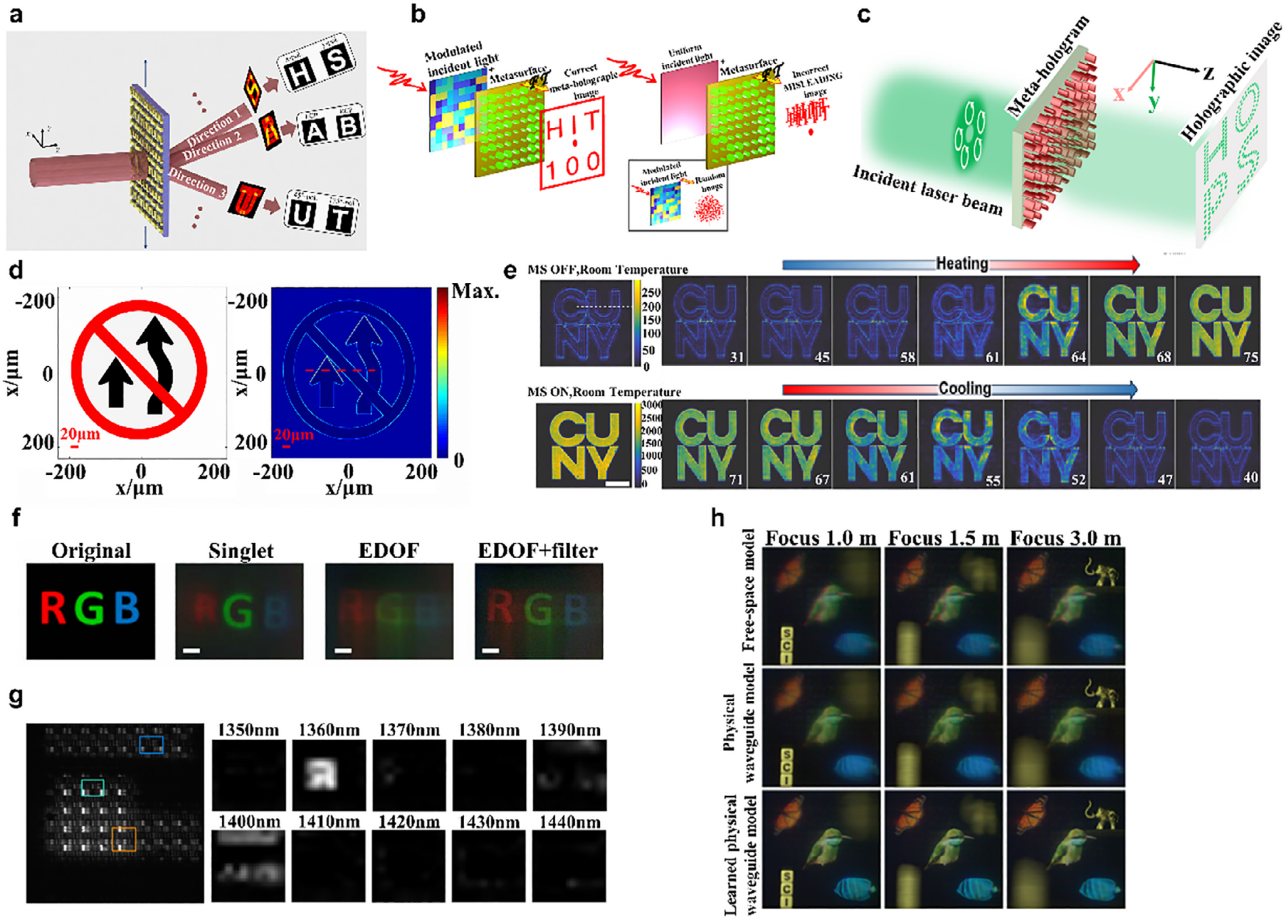
Representative applications of metasurface‐based multidimensional light‐field manipulation in information processing, computational imaging, and display. (a) Multichannel holographic information readout using multidimensional multiplexed PB‐phase metasurfaces. Reprinted under the terms of the CC BY 4.0 license [178]. Copyright 2025, The Authors. (b) Optical encryption and incorrect readout in reprogrammable metasurface holography. Reprinted under the terms of the CC BY 4.0 license [179]. Copyright 2020, The Authors. (c) Multichannel readout using higher‐order Poincaré‐sphere (HOPS)‐multiplexed metasurface holography. Reprinted with permission [181]. Copyright 2024, Elsevier Ltd. (d) Input image and numerical edge‐detection result from an ultrahigh‐NA dielectric metasurface. Reprinted with permission [186]. Copyright 2023, Elsevier B. V. (e) Temperature‐controlled switching between edge detection and bright‐field imaging using a VO_2_ phase‐change metasurface. Reprinted under the terms of the CC BY 4.0 license [187]. Copyright 2024, The Authors. (f) White‐light full‐color image recovery in metasurface computational imaging. Reprinted with permission [204]. Copyright 2018, The American Association for the Advancement of Science. (g) Spatially overlapped image acquisition and multiwavelength reconstruction using quasi‐bound‐state‐in‐the‐continuum (quasi‐BIC) metasurfaces. Reprinted with permission [211]. Copyright 2024, Optica Publishing Group. (h) Full‐color three‐dimensional (3D) holographic augmented‐reality (AR) display based on metasurface waveguides. Reprinted under the terms of the CC BY 4.0 license [212]. Copyright 2024, The Authors.

Optical encryption represents a further extension of multidimensional information encoding. Its central principle is to incorporate the phase matrix of the incident field, polarization state, vectorial‐mode structure, or reconfigurable near‐field channel into the decryption key, such that the target image or hidden information can be selectively recovered only under prescribed readout conditions. In 2020, Qu et al. proposed a reprogrammable metasurface‐holography encryption scheme in which the encrypted information was divided between the phase matrix of the incident field and that of the metasurface [179]. As shown in Figure 6b, the correct image was reconstructed only when the modulated incident field matched the metasurface phase matrix; mismatched readout conditions instead produced deceptive or random images. This strategy enabled updatable encryption keys for information concealment, optical authentication, and reprogrammable encryption. In 2022, Wang et al. introduced a chiral metasurface hologram based on the Born–Kuhn model [180]. By exploiting circular dichroism, the device enabled selective holographic reconstruction in different circular‐polarization channels and further encoded independent images in the transmission and reflection spaces. This readout mechanism, jointly defined by spin state, frequency, and spatial channel, enhanced the selectivity and security of hidden information, providing an effective route toward polarization‐dependent anticounterfeiting, full‐space information concealment, and optical encryption. In 2025, He et al. proposed a higher‐order Poincaré‐sphere (HOPS)‐multiplexed metasurface holography scheme, in which vector beams distributed over the HOPS served as a new class of information‐addressing channels for selective reconstruction of multiple holographic images from a single metasurface [181]. As illustrated in Figure 6c, illumination by HOPS vector beams of different orders produced distinct holographic images, thereby expanding the multiplexing dimensionality of metasurface holography and enabling high‐security optical anticounterfeiting and information encryption. Dynamic and reconfigurable metasurfaces provide an additional route toward optical encryption. In 2020, Rajabalipanah et al. introduced a real‐time terahertz meta‐encryption platform based on polarization‐multiplexed graphene computational holography [182]. Independent and dynamically updatable encrypted information was encoded into two near‐field channels, enabling customized image decryption under different polarization states and real‐time rewriting of the hidden information.

Unlike information encoding and encryption, optical computing seeks to perform direct mathematical operations on incident optical fields using engineered structures, rather than merely storing or displaying information. In 2014, Silva et al. introduced the concept of implementing analogue mathematical operations with metamaterials, providing an important theoretical foundation for subsequent developments in wave‐based analogue computing and metasurface optical processors [183]. This concept was later extended to two‐dimensional metasurface platforms. In 2019, Cordaro et al. demonstrated a mathematical operator based on a high‐index dielectric metasurface [184]. By engineering the spatial transfer function of a silicon nanobeam array, the device performed first‐ and second‐order spatial differentiation and experimentally realized optical edge detection. This work established that metasurfaces can directly process incident images within a subwavelength‐scale thickness, integrating edge‐extraction operations—traditionally requiring complex lens assemblies or digital post‐processing—into an ultrathin optical element. In the same year, Zhou et al. proposed a high‐efficiency dielectric‐metasurface approach to optical edge detection [185]. A PB‐phase‐gradient metasurface was used to implement spatial differentiation of input images, enabling broadband edge detection at multiple visible wavelengths. In 2024, Chen et al. numerically demonstrated a dielectric differential metasurface with an NA of 0.7 for 2D image edge detection [186]. By engineering the optical transfer function of a silicon nanocluster metasurface, the device enhanced the processing of high‐spatial‐frequency features, enabling two‐dimensional edge enhancement and high‐resolution optical differentiation. As shown in Figure 6d, the metasurface numerically produced clearly resolved edge‐detection results from the input image, demonstrating the potential of high‐NA dielectric metasurfaces for all‐optical image processing.

In recent years, metasurface‐based optical computing has evolved from fixed‐function filtering toward switchable and reconfigurable platforms for analogue information processing. In 2024, Cotrufo et al. introduced a reconfigurable image‐processing metasurface based on the phase‐change material VO_2_ [187]. By exploiting the insulator‐to‐metal transition of VO_2_ to modulate the nonlocal optical response of the metasurface, the device enabled thermally controlled switching between edge detection and bright‐field imaging in the near‐infrared regime. As shown in Figure 6e, the insulating state at room temperature produced high‐contrast edge enhancement, whereas heating VO_2_ into its metallic state suppressed the edge‐detection response and yielded an output approaching an unfiltered bright‐field image. Upon cooling, the edge‐detection functionality was restored, highlighting the potential of phase‐change metasurfaces for reconfigurable all‐optical image processing. In 2026, Chen et al. proposed a reconfigurable analogue‐computing metasurface based on thin‐film lithium niobate [188]. Electro‐optic tuning dynamically modified the spatial transfer function of the device, allowing a single platform to switch among first‐order differentiation, second‐order differentiation, and integration. The metasurface was applied to Gaussian‐signal processing and image edge detection, and in the second‐order differentiation mode it resolved edge features of approximately 10 µm. These results underscore the promise of electro‐optically reconfigurable metasurfaces for high‐speed, on‐chip optical computing and real‐time image processing. Related studies have also explored mechanical‐strain tuning, electrically controlled nonlocal responses in graphene, and low‐loss phase‐change materials. By dynamically reconfiguring the spatial‐frequency response or optical transfer function of the metasurface, these approaches have enabled switchable bright‐field imaging, low‐ and high‐pass filtering, edge detection, and spatial differentiation [189, 190, 191]. Collectively, these advances show that metasurface optical computing is progressing beyond static filtering elements toward tunable, reconfigurable, and multifunctional analogue information‐processing platforms.

More recently, metasurface‐based optical computing has begun to advance from device‐level analog operations toward system‐level intelligent information processing. In 2026, Fu et al. demonstrated a monolithic photonic neural network based on an active bound‐state‐in‐the‐continuum (BIC) metasurface, in which optical nonlinearity, long‐range coupling, and intrinsic temporal memory were physically co‐integrated to realize reservoir computing [192]. The platform was experimentally validated for brain magnetic resonance imaging image classification and human‐action recognition, highlighting the potential of metasurfaces as integrated physical computing substrates. In the same year, Peng et al. developed a hybrid photonic‐electronic vision‐processing engine that combined a 41‐million‐parameter optical metasurface front end with a co‐designed 87 000‐parameter digital back end, enabling real‐time object detection, semantic segmentation, three‐dimensional reconstruction, and video understanding in an edge‐deployable system [193]. In 2026, He et al. further advanced high‐capacity optical inference within the broader framework of metasurface and diffractive optical computing by developing an orbital‐angular‐momentum‐multiplexing diffractive neural network, in which orthogonal OAM modes served as parallel information channels for simultaneous multitask recognition [194]. Together, these studies highlight complementary routes toward system‐level integration, from monolithic metasurface‐based neuromorphic computing to co‐designed photonic‐electronic vision systems, illustrating the transition of metasurface optical computing from isolated analog operations toward integrated and deployable intelligent computing platforms.

The role of metasurfaces in optical information processing and computing has progressively expanded from single‐channel image reconstruction to multidimensional information multiplexing, secure encryption, and reconfigurable optical computing. Through coordinated control over multiple degrees of freedom of the optical field, a single metasurface can increase information capacity, enhance selective readout and decoding security, and perform parallel image processing directly in the optical domain. Metasurfaces therefore provide an important platform for high‐capacity, energy‐efficient, and highly integrated information‐processing systems.

### 4.2 Imaging and Display Technologies

Imaging and display technologies directly benefit from the ability of metasurfaces to integrate wavefront shaping, aberration correction, polarization and spectral control, and computational encoding within a single planar element. Conventional systems distribute these functions across multiple bulk optical components, resulting in large footprints, substantial weight, alignment complexity, and limited integration density. By implementing these functions through arrays of subwavelength meta‐atoms, metasurfaces enable compact imaging systems, flat‐panel and holographic displays, and lightweight near‐eye displays for augmented‐ and virtual‐reality applications.

In metalens‐based imaging, arrays of meta‐atoms are engineered to impart the phase profile required for lens‐like focusing, enabling conventional focusing and image‐formation functions within an ultrathin planar architecture. Compared with refractive lenses, metalenses offer markedly reduced thickness and weight, together with enhanced compatibility with integrated platforms, making them attractive for miniature cameras, on‐chip photonic systems, and portable imaging devices [195, 196]. Early efforts focused primarily on improving focusing efficiency, numerical aperture, and imaging resolution. In 2015, Arbabi et al. demonstrated a subwavelength‐thick planar microlens based on a high‐contrast dielectric transmit array [197]. Under different design conditions, the device achieved focal‐spot sizes as small as 0.57*λ* and focusing efficiencies of up to approximately 82%, while maintaining polarization‐insensitive operation. This work established an important foundation for high‐efficiency, high‐numerical‐aperture dielectric metalenses in compact imaging and integrated photonic systems. In 2016, Khorasaninejad et al. fabricated high‐numerical‐aperture visible metalenses using high‐aspect‐ratio TiO_2_ nanostructures [57]. At NA = 0.8, the devices achieved diffraction‐limited focusing at wavelengths of 405, 532, and 660 nm, with corresponding focusing efficiencies of 86%, 73%, and 66%. The metalenses resolved nanostructures with subwavelength separations and enabled imaging with magnifications of up to approximately 170×, delivering image quality comparable to that of commercial high‐NA microscope objectives. These results highlighted the potential of low‐loss dielectric metalenses for high‐resolution microscopy, laser‐based microscopy, and spectroscopic detection. More recently, metalens research has progressed from proof‐of‐concept demonstrations at the micro‐ and nanoscale toward large‐area fabrication and imaging in realistic environments. In 2024, Park et al. reported an all‐glass visible metalens with a diameter of 100 mm and applied it to astronomical imaging [198]. The system directly imaged celestial objects including the North America Nebula and the Moon, demonstrating that large‐aperture metalenses are becoming viable for real‐world imaging and underscoring their potential for lightweight spaceborne optics and large‐area planar imaging systems. Collectively, these advances show that metalenses are evolving beyond isolated focusing demonstrations toward high‐resolution, large‐aperture, and engineering‐ready imaging platforms.

Beyond advances in imaging resolution and aperture size, active reconfigurability has further expanded the functionality of metalens‐based imaging. In 2026, Kim et al. demonstrated an electrically reconfigurable illumination‐engineering metalens by embedding a polyaniline (PANI) thin film within the metalens, enabling voltage‐programmable control of the illumination numerical aperture and continuous transitions among bright‐field, quasi‐dark‐field, and dark‐field imaging modes under incoherent illumination [199]. This work extends metalens functionality from static wavefront manipulation toward active illumination engineering and multimodal imaging.

Chromatic aberration remains a major obstacle to broadband imaging with metalenses. Because the phase response of metasurface meta‐atoms is typically strongly wavelength‐dependent, uncompensated metalenses readily exhibit focal‐length shifts and image blurring under broadband illumination. To address this limitation, a range of strategies has been developed, including dispersion engineering, wavelength‐dependent phase compensation, joint phase‐group‐delay control, and tunable achromatic focusing. In 2017, Shrestha et al. established a library of meta‐atoms with distinct dispersion responses to realize a broadband achromatic metalens in the near‐infrared regime [200]. The device maintained an approximately constant focal length over the wavelength range of 1.3–1.69 µm, thereby effectively suppressing focal drift under broadband illumination. In 2018, Wang et al. proposed a transmissive broadband achromatic metalens for visible light based on GaN integrated‐resonator meta‐atoms [201]. By combining the PB‐phase with wavelength‐dependent phase compensation introduced by the integrated resonant elements, the device preserved an approximately invariant focal length across the 400–660 nm visible range and demonstrated full‐color imaging. This work extended achromatic metalenses from near‐infrared broadband focusing toward visible full‐color imaging. In 2019, Aiello et al. introduced a visible achromatic zoom metalens in which polarization modulation enabled continuous tuning of the focal length [202]. The device further demonstrated white‐light imaging and switching among different focal planes, broadening the prospects of metalenses for dynamic imaging and variable‐focus optical systems. In 2021, Lu et al. proposed a broadband achromatic superoscillatory metasurface that combined geometric and transmission phases to achieve sub‐diffraction focusing at a common focal plane across the 400–700 nm visible spectrum [203]. This approach provided a new design framework for broadband super‐resolution color imaging and machine vision. Collectively, these studies show that achromatic metalenses have progressed from near‐infrared broadband focusing toward visible full‐color imaging, broadband super‐resolution focusing, and dynamically tunable imaging, providing an important foundation for compact broadband optical systems.

Beyond hardware‐level achromatization through nanostructural dispersion engineering, computational imaging and deep learning have opened alternative routes to broadband metasurface imaging. Rather than requiring the metalens to eliminate all aberrations optically, computational imaging adopts an “optical encoding‐algorithmic reconstruction” paradigm, in which image information is recovered computationally to balance device miniaturization against imaging performance. In 2018, Colburn et al. integrated metasurface optics with computational imaging by designing a metalens featuring an extended depth of field and a spectrally quasi‐invariant point‐spread function. Combined with a downstream reconstruction algorithm, the system recovered full‐color images under white‐light illumination [204]. As shown in Figure 6f, this approach markedly improved the recognizability of red–green–blue (RGB) color targets, demonstrating the potential of metalens‐based optical encoding coupled with computational reconstruction to mitigate broadband chromatic aberration and enable compact full‐color imaging systems. In 2023, Maman et al. further combined flat‐lens imaging with deep learning, using a neural‐network model to correct both axial and lateral chromatic aberrations under broadband illumination and thereby achieve high‐quality full‐color image reconstruction in natural‐light scenes [205]. In 2024, Seo et al. integrated a mass‐producible metalens with a diameter of 10 mm into a deep‐learning‐based image‐restoration framework [206]. The method compensated for chromatic and off‐axis aberrations, as well as image degradation arising from fabrication imperfections, and produced full‐color metalens images with quality approaching that of the reference images. Collectively, these studies indicate that metasurface imaging is evolving beyond the optimization of standalone optical components toward a co‐designed architecture that integrates front‐end optical encoding by the metalens, image acquisition by the sensor, and computational reconstruction. This emerging imaging paradigm provides an important foundation for compact full‐color imaging and deployment in real‐world environments.

Recent studies have further advanced the system‐level integration of metasurfaces with hardware and processing, enabling more compact and intelligent optical imaging systems. In 2025, Fröch et al. demonstrated a compact metasurface‐based optical imaging platform in which a 1‐cm‐aperture, *f*/2 broadband visible meta‐optic was directly integrated with a camera and jointly optimized with a learned computational reconstruction backend [207]. This optical‐computational integration enabled polarization‐insensitive full‐color imaging under ambient illumination with a diagonal field of view of approximately 30° and video‐rate exposure times, illustrating how metasurface integration and computational co‐design can mitigate the bandwidth limitations of large‐aperture flat optics. In 2026, Lee et al. demonstrated a highly integrated metasurface‐based neural phase‐microscopy platform that combined a single‐layer complex‐amplitude metasurface, compact optical hardware, and physics‐informed artificial intelligence (AI) algorithms [208]. The metasurface integrated multiple optical functions into a single planar element, replacing bulky interferometric optics and active modulators and enabling single‐shot quantitative phase imaging with a resolution better than 840 nm at 74 Hz. Together, these studies highlight complementary routes toward system‐level integration in metasurface imaging, from computationally co‐designed meta‐optic cameras to AI‐assisted metasurface‐based phase microscopy, illustrating the transition of metasurface imaging from standalone optical components toward compact and jointly optimized optical‐computational systems.

Metasurfaces also offer compelling advantages for polarization and spectral imaging. Polarization carries information about material stress, surface reflection, scattering behavior, and chiral response that is often inaccessible to conventional intensity‐based imaging, whereas spectral information enables material identification, target detection, and portable spectroscopic analysis. Traditional polarization‐ and spectral‐imaging systems typically rely on multiple optical components, repeated measurements, or complex filtering architectures, making miniaturization, real‐time operation, and on‐chip integration difficult to achieve. In 2019, Rubin et al. introduced a dielectric‐metasurface polarization‐analyzing grating in which different diffraction orders corresponded to distinct polarization‐analysis channels [209]. This architecture produced multiple polarization‐encoded subimages in a single exposure and enabled reconstruction of the full Stokes vector, providing a new route toward compact full‐Stokes polarization cameras. In 2023, Zuo et al. further integrated a metasurface polarization‐filter array directly onto a CMOS image sensor to realize MetaPolarIm, an on‐chip full‐Stokes polarization‐imaging sensor [210]. The device achieved single‐shot full‐Stokes imaging in both the red and green spectral bands, establishing a practical pathway toward high‐speed, CMOS‐compatible, on‐chip polarimetric imaging. In 2024, Shao et al. proposed a compact multispectral imaging device based on a quasi‐BIC metasurface [211]. Arrays of meta‐atoms with distinct resonant responses were used to modulate the near‐infrared spectrum, enabling high‐resolution spectral reconstruction across the 1340–1450 nm wavelength range. As shown in Figure 6g, the device reconstructed spatial images at different wavelength channels from a single acquisition of spatially overlapping infrared images, highlighting the potential of metasurfaces for miniaturized multispectral imaging and portable spectroscopic sensing. Collectively, these studies show that metasurfaces can not only replace or simplify conventional polarization‐ and spectral‐analysis components, but also be integrated with image sensors and computational reconstruction algorithms to drive imaging systems toward greater compactness, multichannel functionality, and real‐time operation.

In near‐eye augmented‐reality (AR) displays, metasurfaces can perform waveguide coupling, beam steering, chromatic‐aberration correction, virtual‐real image fusion, and overall system miniaturization. Contemporary AR platforms must deliver wide fields of view, full‐color imagery, and low‐aberration transmission within a tightly constrained form factor, placing stringent demands on optical couplers, color correction, and system‐level integration. In 2024, Gopakumar et al. developed a full‐color three‐dimensional holographic AR display that integrated inverse‐designed metasurface grating couplers, a compact dispersion‐compensating waveguide architecture, and artificial‐intelligence‐driven holographic algorithms [212]. As shown in Figure 6h, the system enabled virtual 3D images to be overlaid onto real‐world scenes in an optical see‐through AR configuration, highlighting the potential of metasurface waveguides for system‐level integration in near‐eye displays. In 2025, Tian et al. proposed an achromatic metasurface waveguide for full‐color AR displays [213]. By combining inverse‐designed input and output metasurface couplers with a monolithic high‐index waveguide, the system achieved wide‐field full‐color beam coupling while substantially improving chromatic aberration, color uniformity, and angular uniformity. This architecture offers a promising route toward compact full‐color near‐eye displays. Dynamic reconfigurability has also begun to extend metasurface displays beyond static wavefront manipulation. In 2022, Tang et al. combined electrically driven liquid‐crystal tuning with a layer‐folded metasurface to construct a dynamic AR holographic display [214]. Distinct holographic images could be switched within a real scene by varying the applied voltage, demonstrating the transition of metasurface display devices toward actively tunable and dynamic operation. Collectively, these studies show that metasurfaces in AR near‐eye displays can serve not only as waveguide couplers and chromatic‐correction elements, but also as platforms that integrate computational holography and dynamic modulation. This convergence is driving near‐eye display systems toward lighter weight, full‐color operation, and reconfigurable functionality.

In three‐dimensional display applications, metasurfaces can exploit metalens arrays, metagratings, and multi‐polarization wavefront control to enable near‐eye 3D displays, full‐color video‐rate displays, and multiview autostereoscopic displays. These approaches show considerable promise for white‐light three‐dimensional image reconstruction, broadband and low‐crosstalk holography, reduced visual fatigue, miniaturized near‐eye holography, and wide‐field‐of‐view three‐dimensional visualization [215, 216, 217, 218, 219]. In 2024, Fan et al. developed an integral‐imaging near‐eye 3D display based on a nanoimprinted metalens array [220]. By integrating a large‐area metalens array with a commercial microdisplay, the system achieved video‐rate, true three‐dimensional near‐eye display at an average frame rate of 67 frames per second, while maintaining a display‐module weight of approximately 7.23 g. The authors further constructed an optical see‐through AR prototype that reproduced motion parallax and monocular accommodation cues, demonstrating the potential of metalens arrays for lightweight and perceptually realistic near‐eye 3D displays. In the same year, Ahmad et al. proposed an ultrathin metasurface for ultra‐multiview 3D displays [221]. Through coordinated wavefront control of linearly and circularly polarized light, the device multiplexed viewpoints across multiple polarization channels. Numerical demonstrations showed the reconstruction of 24 distinct viewpoints distributed among the linear‐polarization, left‐handed circular‐polarization, and right‐handed circular‐polarization channels. Collectively, these studies highlight the substantial potential of metasurfaces for lightweight near‐eye 3D displays, multiview glasses‐free visualization, and immersive display systems.

The application of metasurfaces in imaging and display technologies has progressed from early demonstrations of single‐function focusing and basic image formation toward system‐level capabilities, including broadband achromatic imaging, computationally enhanced imaging, multidimensional polarization and spectral information acquisition, AR near‐eye displays, and three‐dimensional visualization. Through multidimensional light‐field manipulation and the co‐design of optical hardware and computational algorithms, metasurfaces provide a new technological pathway toward lighter, flatter, more highly integrated, and increasingly intelligent imaging and display systems.

### 4.3 Optical Communication and Information Transmission

Optical communication and information transmission increasingly exploit metasurfaces as compact and programmable interfaces for channel generation, beam routing, signal multiplexing, and adaptive link control. The rapid development of high‐speed optical communication, terahertz communication, millimeter‐wave communication, and sixth‐generation (6G) wireless networks places increasingly stringent demands on channel capacity, energy efficiency, dynamic reconfigurability, and system‐level integration. These requirements are difficult to meet using conventional architectures based on bulk optical components, complex antenna arrays, and radio‐frequency front ends. By encoding and multiplexing information across wavelength, polarization, orbital angular momentum, and propagation direction, metasurfaces enable vortex‐beam optical communication, terahertz information transmission, programmable modulation, and multiplexed multi‐user communication.

In optical communication, OAM provides an important degree of freedom for increasing system capacity. OAM‐carrying vortex beams possess helical phase fronts, and OAM modes with different topological charges are mutually orthogonal under ideal conditions, allowing them to serve as independent information carriers for mode‐multiplexed transmission. In 2004, Gibson et al. presented an early free‐space information‐transmission system based on OAM‐state encoding [222]. By employing eight distinct OAM states over a free‐space link of approximately 15 m, they demonstrated the feasibility of using OAM modes as an additional dimension for optical information encoding. In 2012, Wang et al. experimentally demonstrated terabit‐scale free‐space optical communication based on OAM multiplexing [223]. By combining OAM mode multiplexing with polarization multiplexing and spatial multiplexing, the system achieved an aggregate transmission rate of up to 2.56 Tbit s^−1^, showing that OAM multiplexing can substantially increase the channel capacity and spectral efficiency of free‐space optical links. To address mode crosstalk and transmission instability induced by atmospheric turbulence in practical links, Zhu et al. proposed an OAM mode‐group multiplexing scheme in 2023, improving the robustness of OAM‐multiplexed free‐space communication under turbulent conditions [224]. Collectively, these studies establish that OAM can function not only as an information‐encoding state but also as a set of parallel transmission channels, thereby laying the application foundation for compact metasurface devices capable of OAM‐beam generation, multiplexing, and information transmission.

Metasurfaces provide a compact device platform for OAM‐beam generation, multidimensional multiplexing, and information encoding. Conventional OAM‐beam generators typically rely on spatial light modulators or bulk phase elements, which are difficult to reconcile with integrated and chip‐scale architectures. By contrast, metasurfaces can directly impart helical and focusing phase profiles to incident light, while also enabling complex‐amplitude modulation through the spatial arrangement of subwavelength elements. In 2019, Jiang et al. introduced a monolithic, non‐interleaved metasurface vortex multiplexer that exploited the coherent superposition of all meta‐atoms within a shared aperture to achieve high‐capacity and flexible mode multiplexing of higher‐order Poincaré sphere beams [225]. The device generated vector vortex beams with 12 and 16 independent channels, achieving total experimental efficiencies of approximately 69% and 65%, respectively. This work established a new design strategy for multimode vector‐beam multiplexing and high‐capacity information transmission using planar photonic platforms. In 2024, Liu et al. proposed a longitudinal encoding scheme based on focused vector vortex beams generated by a silicon metasurface [226]. Through joint control of the transmission‐phase and PB‐phase, combined with a spin‐polarization multiplexing mechanism, the device generated OAM superposition states carrying prescribed encoded information at distinct focal planes, thereby enabling information encoding and decoding along the propagation direction. By incorporating the longitudinal spatial dimension into vector‐vortex‐beam encoding, this approach further expanded the potential of metasurfaces for multidimensional optical information encoding and encrypted transmission.

In terahertz and millimeter‐wave information transmission, OAM is regarded as an important degree of freedom for increasing channel capacity and spectral efficiency because of the orthogonality among distinct OAM modes. In 2014, Yan et al. provided an early experimental demonstration of OAM‐multiplexed communication at millimeter‐wave frequencies [227]. At a carrier frequency of 28 GHz, the system employed eight OAM channels distributed across two orthogonal polarizations to achieve a data transmission rate of 32 Gbit s^−1^ and a spectral efficiency of approximately 16 bit s^−1^ Hz^−1^, thereby providing system‐level validation of OAM mode multiplexing for wireless communication. Compared with millimeter waves, terahertz waves offer higher carrier frequencies and substantially larger available bandwidths, making them promising for future high‐speed wireless communication. However, terahertz functional devices continue to face challenges associated with high loss, limited beam‐control capability, and insufficient integration density. Metasurfaces can flexibly manipulate the phase, amplitude, and polarization of terahertz waves within a low‐profile architecture, providing an important platform for terahertz OAM‐beam generation, multiplexing, and integrated communication devices. In 2021, Yang et al. proposed a broadband transmissive terahertz vortex‐beam generator based on a thermally tunable PB‐phase metasurface [228]. By varying the temperature of the InSb meta‐atoms, the operating frequency was tuned across 1.8–4.5 THz, corresponding to a fractional bandwidth of 85.7%, while high‐purity OAM beams with topological charges of +1 and −2 were generated. This work demonstrated that thermally tunable semiconductor materials can enable reconfiguration of the operating frequency of terahertz OAM devices without altering their geometrical architecture, offering an effective route toward broadband and tunable terahertz OAM components.

Terahertz OAM metasurfaces have further evolved from single‐function vortex‐beam generators toward platforms capable of dynamic full‐space control, multimode multiplexing, and high‐efficiency output. In 2022, Yang et al. proposed a VO_2_‐assisted terahertz vortex‐beam generator in which the phase transition of vanadium dioxide enabled dynamic switching between transmission and reflection modes [229]. As shown in Figure 7a, the device generated diagonally directed vortex beams, four‐beam vortex arrays, and focused vortex beams in a transmission mode at 1.26 THz and a reflection mode at 1.06 THz. By enabling coordinated control of OAM beams in both the transmission and reflection half‐spaces, this work introduced a new device architecture for full‐space terahertz OAM manipulation and high‐capacity information multiplexing. In 2024, Chong et al. further demonstrated a polarization‐multiplexed terahertz OAM comb based on an all‐silicon metasurface [230]. As illustrated in Figure 7b, anisotropic silicon‐pillar meta‐atoms independently manipulated *x‐* and *y*‐polarized incident waves, enabling a single metasurface to generate polarization‐multiplexed OAM combs composed of multiple equally spaced modes with nearly uniform intensities. The number of modes, the position of the OAM spectrum, and the mode spacing could all be engineered, providing a new route toward multimode, multi‐polarization terahertz communication and high‐dimensional information encoding. In 2025, Xuan et al. proposed a high‐performance transmissive terahertz metasurface for focused‐vortex‐beam generation [231]. Using an all‐dielectric silicon‐pillar architecture, the device experimentally achieved a focusing efficiency of 91% and an OAM mode purity of 93.9%. The authors further numerically demonstrated a focused‐vortex‐beam deflector and a dual‐focus vortex metasurface. Collectively, these advances show that terahertz OAM metasurfaces are progressing beyond early single‐function beam generators toward integrated information‐manipulation platforms that combine broadband tunability, dynamic full‐space control, polarization multiplexing, and high‐efficiency operation. Such platforms provide important support for future high‐capacity terahertz communication, high‐dimensional information processing, and on‐chip integrated systems.

**FIGURE 7:**
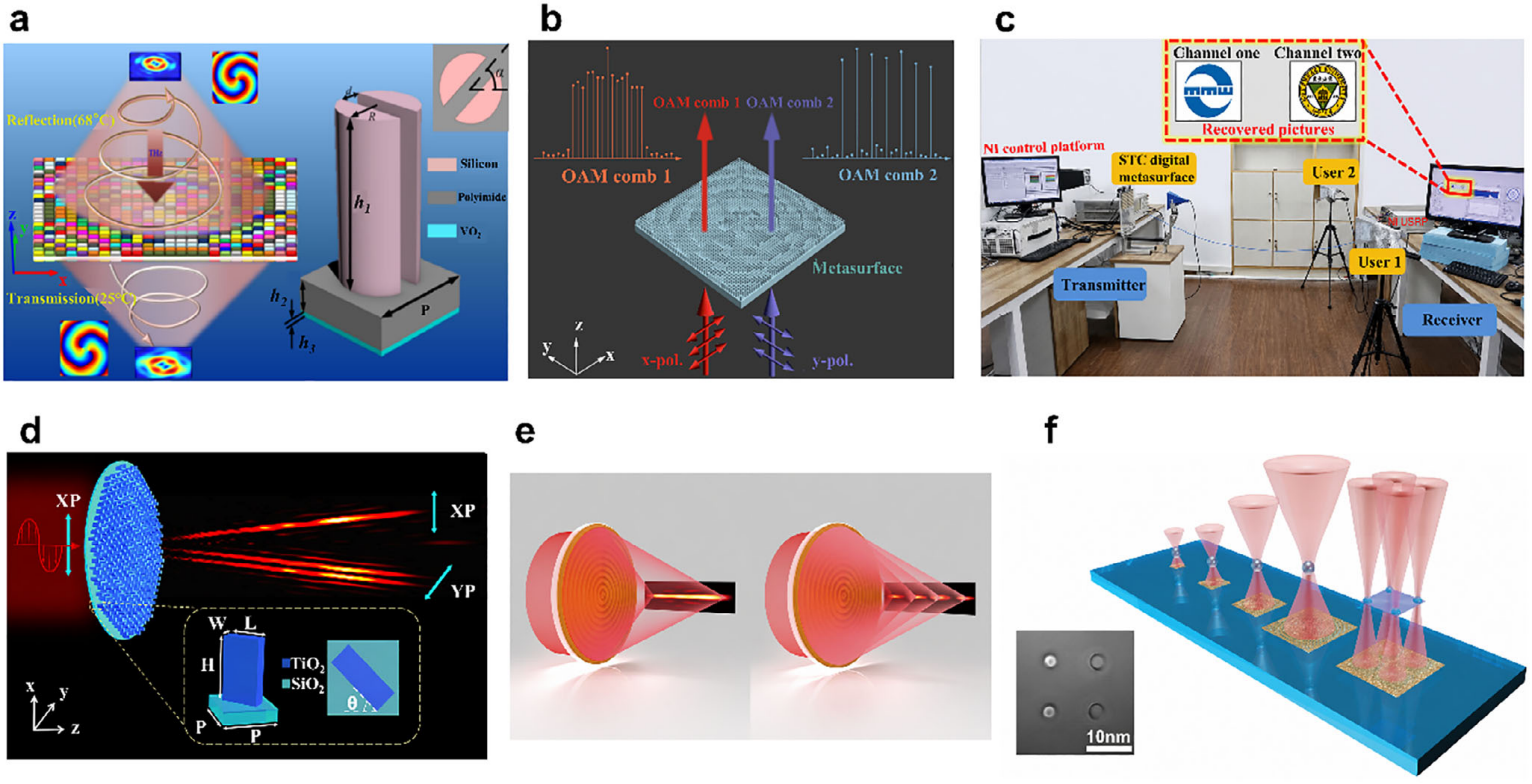
Representative applications of metasurface‐based multidimensional light‐field manipulation in optical communication and structured optical‐field manipulation. (a) Transmission/reflection OAM beam control using VO_2_‐assisted terahertz metasurfaces. Reprinted with permission [229]. Copyright 2022, Optica Publishing Group. (b) Polarization‐multiplexed terahertz OAM comb generation using all‐silicon metasurfaces. Reprinted under the terms of the CC BY 4.0 license [230]. Copyright 2024, The Authors. (c) Dual‐channel image wireless transmission based on space–time‐coding digital metasurfaces. Reprinted with permission [234]. Copyright 2021, The Authors, under exclusive licence to Springer Nature Limited. (d) Polarization‐rotating Bessel beams generated by all‐dielectric metasurfaces. Reprinted with permission [251]. Copyright 2023, Elsevier B.V. (e) Super‐resolved optical needles and axial multifocal arrays generated by graphene oxide metalenses. Reprinted under the terms of the CC BY 4.0 license [253]. Copyright 2021, The Authors. (f) High‐NA reflective metasurface optical tweezers and multipoint trapping. Reprinted under the terms of the CC BY 4.0 license [258]. Copyright 2023, The Authors.

Beyond OAM multiplexing, programmable metasurfaces and space–time‐coding metasurfaces offer alternative architectures for information‐transmission systems. By using external electrical control or time coding to reconfigure the electromagnetic responses of individual elements in real time, these metasurfaces can directly implement beam scanning, multibeam generation, signal modulation, and information loading at the physical layer. Such capabilities can reduce the complexity associated with radio‐frequency chains, mixers, and antenna arrays in conventional wireless communication systems. In 2020, Chen et al. proposed an amplitude‐modulation architecture based on a time‐varying programmable metasurface [232]. A field‐programmable gate array controlled the switching states of PIN diodes, enabling modulation of the radiated amplitude while maintaining an essentially stable phase response. Experiments at 5.85 GHz revealed a pronounced amplitude‐modulation envelope, validating the feasibility of using programmable metasurfaces to directly perform information loading and signal modulation. This work introduced a promising route toward simplified wireless transmitters. In 2023, Lan et al. developed a real‐time programmable terahertz metasurface based on gallium nitride technology, operating at approximately 0.34 THz [233]. Using 1‐bit digital coding sequences, the device achieved wide‐angle beam scanning, multibeam generation, and diffuse‐wavefront manipulation. It enabled beam scanning with an angular precision of approximately 1° across a 70 GHz bandwidth, together with switching speeds of up to 100 MHz. The authors further demonstrated point‐to‐point tracking transmission of terahertz signals, highlighting the potential of real‐time programmable metasurfaces for terahertz communication, dynamic beamforming, and mobile networking.

Building on these advances, digital coding and space–time‐coding metasurfaces have further driven information‐transmission systems from the manipulation of electromagnetic waves toward direct information modulation. In 2021, Zhang et al. proposed a spatial‐frequency‐multiplexed wireless communication scheme based on a space–time‐coding digital metasurface [234]. By engineering the space–time coding matrix to control the harmonic frequency and radiation direction simultaneously, the system independently transmitted distinct digital information streams to users at different spatial locations. As shown in Figure 7c, a dual‐channel communication link was constructed using a 2‐bit space‐time‐coding digital metasurface, enabling the real‐time transmission of two images to two users at a data rate of 2.5 Mbps. This demonstration highlighted the value of metasurfaces for wireless communication exploiting both spatial diversity and frequency diversity. In 2022, Wan et al. further demonstrated the joint modulation of electromagnetic waves and digital signals on a single programmable metasurface platform [235]. The system enabled beam‐steerable single‐channel communication and the independent transmission of information to multiple users, providing system‐level validation that a programmable metasurface can simultaneously perform beam control and information modulation. In the same year, Ke et al. introduced a spatial‐frequency‐polarization multiplexing wireless communication system based on an anisotropic space–time‐coding digital metasurface [236]. By independently engineering time‐varying control‐voltage sequences for different polarization orientations, the system achieved joint multiplexing across the frequency, polarization, and spatial dimensions. Parallel transmission of multiple information streams was realized on a compact dual‐polarization metasurface platform, increasing channel capacity and improving the utilization of spatial resources while offering a new architecture for coordinated multi‐user wireless communication. More broadly, programmable and space–time‐coding metasurfaces integrate beamforming, spectrum control, polarization multiplexing, and digital information loading within a single physical platform, providing an important route toward low‐complexity, high‐capacity, and intelligent wireless communication systems. Metasurface‐based communication architectures are also evolving from basic wavefront manipulation toward adaptive link optimization. Low‐sidelobe dual‐beam antennas, broadband achromatic anomalous‐reflection metasurfaces, and adaptively programmable metasurfaces have respectively employed independent amplitude‐phase control, global optimization of aperiodic reflective apertures, and closed‐loop sensing‐analysis‐control to achieve multibeam sidelobe suppression, broadband angle‐stable beam redirection, and adaptive communication‐quality enhancement in complex and non‐line‐of‐sight environments [237, 238, 239].

The application of metasurfaces in optical communication and information transmission has progressed from early OAM‐state encoding and multiplexed free‐space transmission toward terahertz vortex‐beam manipulation, programmable signal modulation, space‐time‐coded multi‐user communication, and intelligent link optimization. Compared with conventional spatial light modulators, bulk phase elements, and complex antenna arrays, metasurfaces offer a low‐profile architecture, straightforward integration, multidimensional manipulation, and programmable reconfigurability, making them promising device platforms for future high‐speed optical communication, terahertz communication, and 6G wireless networks. Nevertheless, several critical challenges must be addressed before widespread practical deployment, including OAM mode crosstalk, long‐distance transmission stability, broadband and high‐efficiency manipulation, real‐time response speed, and system‐level integration.

### 4.4 Structured Optical‐Field Manipulation and Optical Manipulation

Structured optical‐field manipulation and optical manipulation represent major application domains of metasurface‐based multidimensional light‐field manipulation. Structured optical fields typically exhibit spatially varying distributions of phase, amplitude, polarization, or angular momentum, encompassing vector beams, vortex beams, optical skyrmions, Bessel beams, Airy beams, optical needles, and multifocal optical fields. These tailored fields offer considerable value for high‐resolution imaging, light–matter interactions, the trapping of micro‐ and nanoparticles, and on‐chip optical manipulation. Conventional methods for generating structured optical fields generally rely on spatial light modulators, q‐plates, axicons, or cascaded assemblies of multiple optical components, resulting in bulky systems with limited integration density. By enabling control over multiple degrees of freedom within an ultrathin planar architecture, metasurfaces provide a promising technological route toward the compact generation of structured optical fields and the system‐level integration of optical manipulation platforms.

For vectorial optical fields and polarization‐structured light, metasurfaces can exploit PB‐phase, propagation‐phase, and anisotropic responses to precisely tailor spatial polarization distributions, providing an effective platform for the compact generation of structured optical fields such as radially polarized light, azimuthally polarized light, cylindrical vector beams, and vector vortex beams. In 2016, Yue et al. introduced a monolithic plasmonic‐metasurface structured‐beam generator that produced radially and azimuthally polarized vortex beams through local modulation of the phase and transverse polarization distribution [240]. This work demonstrated that a single metasurface can replace conventional cascaded optical components for the compact generation of vector‐vortex structured optical fields. In 2017, He et al. further proposed a switchable singular‐beam generator based on a dielectric metasurface, enabling the generation of and conversion among vortex beams, cylindrical vector beams, and cylindrical vector vortex beams at a wavelength of 1550 nm [241]. The system could flexibly switch between optical fields containing phase singularities and polarization singularities. The average conversion efficiency from cylindrical vector beams to vortex beams was approximately 47.7%, whereas that from vortex beams to cylindrical vector vortex beams reached 82.4%, highlighting the versatility of dielectric metasurfaces for manipulating multiple classes of structured optical fields. In 2021, Wang et al. developed a method for generating complex vectorial optical fields using a spatially varying full‐Jones‐matrix metasurface, enabling simultaneous control of the wavefront and local polarization distribution [242]. Beyond producing far‐field vectorial optical fields with vortex wavefronts and spatially varying elliptical‐polarization distributions, the approach also generated cylindrical vector vortex surface‐plasmon‐polariton waves carrying orbital angular momentum. This work established a general platform for the compact near‐ and far‐field generation of arbitrarily prescribed vectorial optical fields.

For the generation of vortex and singular structured optical fields, metasurfaces can produce not only individual vortex beams, but also ring vortex beams, perfect vortex beams and their composite forms, as well as complex vortex optical fields with spatially nonuniform singularity distributions. In 2020, Han et al. proposed a reflective metasurface for the generation and superposition of ring vortex beams [243]. By integrating an axicon phase, a helical phase, and a linear phase gradient within a single device, the metasurface enabled both the generation and spatial separation of ring vortex beams. The superposition state of different ring vortex beams could be continuously controlled by varying the polarization state of the incident light, and polarization‐state measurement was further demonstrated. This approach provided a new route toward polarization sensing and the manipulation of complex vortex structured optical fields. In 2022, Ahmed et al. introduced a multichannel metasurface for generating grafted perfect vortex beams [244]. Using a monolithic bilayer geometric metasurface operating at 650 nm, the device produced superpositions of grafted perfect vortex beams comprising different combinations of topological charges across four spatial channels. It enabled nonuniform OAM distributions and asymmetric singularity patterns, while the singularity positions could be tuned by introducing an initial phase difference. This work established a new structured‐light platform for generating complex singular optical fields, with potential applications in singular optics and quantum science. Related studies on perfect vector vortex beams and high‐topological‐charge OAM laser emission have further expanded the application scope of metasurfaces in complex vortex optical fields and singular optics [245, 246]. Collectively, these advances show that metasurface‐based vortex and singular‐field manipulation is progressing beyond the simple loading of a single helical phase toward ring‐shaped optical fields, perfect vortices, multichannel multiplexing, multiple‐singularity distributions, and increasingly sophisticated control of optical angular momentum.

Beyond conventional structured light such as vector and vortex beams, recent metasurface research has extended toward topologically nontrivial structured optical fields, among which optical skyrmions have attracted increasing attention. In 2024, He et al. demonstrated a metafiber‐integrated photonic skyrmion generator based on a polarization‐dependent metasurface on a fiber tip, enabling a switchable optical skyrmion with subwavelength polarization features [247]. In 2025, Jacob et al. introduced an all‐dielectric chiral metasurface capable of generating cylindrical vector vortex beams and optical Stokes skyrmions using polarization‐dependent PB‐phase modulation [248]. More recently, Liu et al. extended metasurface‐enabled skyrmion generation into the quantum regime by demonstrating a chip‐integrated metasurface‐quantum‐emitter platform for room‐temperature generation of single‐photon skyrmions, including high‐order anti‐skyrmion and skyrmionium states [249]. These developments illustrate the expansion of metasurface‐based structured‐light manipulation from conventional vectorial and vortex fields toward topologically nontrivial structured light and, more recently, quantum skyrmionic states.

For non‐diffracting beams and three‐dimensional focal‐field manipulation, metasurfaces can implement planar phase distributions to enable the compact generation of structured optical fields such as Bessel and Airy beams. In 2017, Chen et al. demonstrated wavelength‐independent subwavelength Bessel‐beam generation using a dielectric metasurface [250]. The resulting meta‐axicon exhibited a numerical aperture of up to 0.9 and generated Bessel beams with a full width at half maximum of approximately *λ*/3, while maintaining an approximately wavelength‐invariant transverse intensity distribution across the visible spectrum. This work provided a compact source of non‐diffracting beams for laser processing, imaging, and optical manipulation. In 2024, Zhi et al. designed and numerically demonstrated polarization‐rotating Bessel beams and corresponding beam arrays based on an all‐dielectric PB‐phase metasurface, offering a complementary strategy for polarization encoding and multichannel manipulation of non‐diffracting optical fields [251], as shown in Figure 7d. In 2026, Wu et al. designed a tunable terahertz non‐diffracting‐beam metasurface based on vanadium dioxide and photosensitive silicon [252]. Through thermal and optical‐intensity control, the device enabled the reconfigurable generation of two‐dimensional Airy beams, circular Airy beams, and zeroth‐ to second‐order Bessel beams. Numerical simulations further verified non‐diffracting propagation, self‐bending, self‐healing, and self‐focusing behavior. Collectively, these studies indicate that metasurface‐based control of non‐diffracting beams is expanding from static visible‐light devices toward dynamically reconfigurable terahertz structured optical fields.

Beyond non‐diffracting propagation, metasurfaces can exploit complex‐amplitude modulation, three‐dimensional phase engineering, and tunable material responses to generate optical needles, multifocal arrays, and complex focal‐field distributions. In 2021, Wang et al. demonstrated a super‐resolved optical needle and an axial multifocal array using a graphene‐oxide metalens [253]. Through simultaneous phase and amplitude modulation, the device generated an extended‐depth‐of‐focus optical needle with a length of approximately 16*λ*, together with four axially distributed focal spots. As shown in Figure 7e, the experimentally measured intensity distribution in the *x–z*‐plane confirmed the ability of the device to manipulate both extended‐depth‐of‐focus and axial multifocal optical fields. This work provided a new strategy for compact focal‐field engineering in microscopic imaging, light‐field shaping, and optical manipulation. In 2026, Cui et al. proposed a three‐dimensionally tunable metalens array based on the phase‐change material Sb_2_S_3_ [254]. Reversible refractive‐index modulation enabled dynamic switching of focal spots throughout three‐dimensional space. The authors further constructed a 2 × 2 metalens array that could be reconfigured between “X”‐shaped and “Y”‐shaped multifocal trapping configurations, offering a new design framework for ordered multiparticle assembly, cell patterning, and biological micromanipulation. Collectively, these studies show that metasurface focal‐field manipulation is progressing beyond single‐focus and static‐field generation toward extended‐depth‐of‐focus optical needles, multifocal arrays, dynamically tunable three‐dimensional focal spots, and reconfigurable array‐based trapping architectures.

In optical tweezers, optical torque, and micro/nano‐manipulation, metasurfaces can integrate tight focusing, multifocal generation, vortex‐beam manipulation, and polarization multiplexing within an ultrathin device, thereby providing a compact route to particle trapping, translation, rotation, and parallel multiparticle manipulation. In 2017, Ma et al. proposed a multifocal optical‐vortex metalens for nanoparticle trapping and manipulation [255]. The device generated multiple focused vortex optical fields carrying prescribed OAM at distinct spatial positions, and its capability to trap and manipulate different classes of nanoparticles was numerically demonstrated. This work provided a new design strategy for integrated optical tweezers and lab‐on‐a‐chip systems. In 2020, Chantakit et al. experimentally demonstrated a two‐dimensional optical‐tweezers system based on an all‐dielectric silicon metalens [256]. The system enabled polarization‐sensitive two‐dimensional translation and release of polystyrene microspheres in water. Using a vortex metalens, the authors further observed the transfer of OAM to the particles and their motion along annular trajectories, validating the feasibility of all‐dielectric metasurface optical tweezers for miniaturized biophotonic manipulation. In 2022, Li et al. experimentally realized optical trapping and manipulation using a multifunctional metasurface [257]. Through joint control of the transmission‐phase and PB‐phase, incident light with opposite circular polarizations generated either a focused Gaussian beam or a focused annular beam carrying OAM. The system enabled microsphere trapping and translation, orbital motion of microparticles, and angular‐momentum‐driven particle spinning, demonstrating that metasurface optical tweezers can combine optical gradient forces, orbital angular momentum, and SAM to support multimodal microparticle manipulation. In 2023, Xiao et al. reported reflective holographic‐metasurface on‐chip optical tweezers with a numerical aperture of up to 1.2 [258]. As shown in Figure 7f, high‐NA focusing combined with multifocal holographic design enabled multisite trapping of extended microstructures, with a maximum trap stiffness of approximately 430 pN µm^−1^ W^−1^. These results highlight the potential of metasurface optical tweezers for high‐stiffness trapping, on‐chip integration, and multisite manipulation of microstructures. Collectively, these advances show that metasurface optical tweezers are progressing beyond single‐focus trapping toward vortex‐driven operation, parallel multifocal manipulation, angular‐momentum control, and on‐chip integrated optical manipulation.

The application of metasurfaces in structured optical‐field manipulation and optical manipulation has evolved from single‐phase loading and static beam generation toward multifunctional control of vectorial optical fields, vortex and singular optical fields, non‐diffracting beams, three‐dimensional focal fields, on‐chip optical tweezers, optical skyrmions, and other topologically structured fields [259, 260]. Through the coordinated design of phase, amplitude, polarization, angular momentum, and material responses, metasurfaces can generate complex structured optical fields within an ultrathin platform and harness them for the trapping, transport, rotation, and parallel manipulation of micro‐ and nanoparticles. Further advances are nevertheless required in device efficiency, dynamic tuning range, optical field stability, optical‐force calibration accuracy, and compatibility with liquid and biological environments. The representative application areas, principal controlled degrees of freedom, key performance metrics, advantages, and technical bottlenecks are summarized in Table 3.

**TABLE 3:** Applications and performance characteristics of metasurface‐based multidimensional light‐field manipulation.

Application area	Primary optical degrees of freedom	Key performance metrics	Representative functions	Major advantages	Key challenges	Refs.
Optical information processing and computing	Phase, amplitude, polarization, and wavelength	Information capacity, reconstruction and computational accuracy, inter‐channel crosstalk, numerical aperture, efficiency, response speed, and dynamic reconfigurability	Multidimensional information encoding and multiplexing, optical encryption, analogue optical computing (spatial differentiation and edge detection), and reconfigurable image processing	High information capacity and parallelism, low processing latency, and compatibility with compact system integration	Trade‐offs among information capacity, inter‐channel crosstalk, efficiency, and fidelity; limited spatial bandwidth and dynamic programmability	[174, 178, 186, 187]
Imaging and display	Phase, polarization, and wavelength	Spatial resolution, field of view, numerical aperture, imaging efficiency, spectral resolution, color fidelity, and inter‐channel crosstalk	Metalens imaging and computational imaging, full‐color holographic augmented‐reality and three‐dimensional displays, and multispectral and polarization imaging	Ultrathin form factor, compact system architecture, high integration density, and diverse information channels	Chromatic dispersion, speckle, and off‐axis aberrations; trade‐offs among field of view, efficiency, color uniformity, and image quality; and challenges in large‐aperture fabrication and system‐level integration	[197, 204, 210, 211, 212]
Optical communication and information transmission	Polarization, OAM, space, and frequency	Channel capacity, bit‐error rate, mode crosstalk, insertion loss, OAM mode purity, and response speed	OAM mode generation and multidimensional multiplexing, dynamic beam control, Spatial‐frequency multiplexing, and multi‐user communication	Scalable multiplexing dimensionality, increased channel capacity, and support for dynamically reconfigurable communication links	OAM mode stability and beam divergence, inter‐channel crosstalk, insertion loss, transmitter–receiver alignment, and system compatibility	[229, 230, 233, 234]
Structured optical‐field manipulation and optical manipulation	Phase, amplitude, polarization, and OAM	Mode purity, polarization‐conversion efficiency, numerical aperture, focal‐field size, non‐diffracting propagation length, and trap stiffness	Generation of vector vortex and Bessel beams, construction of optical needles and multifocal arrays, and optical trapping and multisite microparticle manipulation	Customizable light‐field modes, compact form factor, high functional integration, and suitability for on‐chip optical manipulation	Limited conversion efficiency, insufficient dynamic reconfigurability, constraints on mode purity and propagation stability, and challenges in achieving long working distances and large‐scale parallel manipulation	[240, 251, 253, 258]

## 5 Challenges and Future Directions

Metasurface‐based multidimensional light‐field manipulation provides a new technical platform for optical information processing, imaging and display, optical communication, and structured optical‐field manipulation. Through coordinated control of phase, amplitude, polarization, wavelength, spatiotemporal mode, and OAM, metasurfaces can realize multichannel, multifunctional, and highly integrated light‐field manipulation that is difficult to achieve with bulk optics. However, as the number of controllable degrees of freedom and application demands increase, metasurfaces face challenges in efficiency, operating bandwidth, inter‐channel crosstalk, dynamic reconfigurability, fabrication precision, scalable manufacturing, and system‐level integration [261, 262].

From the perspective of multidimensional co‐design, inter‐channel crosstalk arises from the incomplete physical independence of different optical degrees of freedom. In complex‐amplitude modulation and polarization‐, wavelength‐, and OAM‐multiplexed metasurfaces, the same geometrical parameters can simultaneously affect phase, amplitude, polarization‐conversion efficiency, dispersion, and mode purity. Moreover, near‐field coupling between neighboring meta‐atoms can cause the actual optical responses to deviate from those predicted using periodic unit‐cell models, thereby degrading the designed wavefront response and increasing inter‐channel crosstalk [263, 264]. Recent inverse‐design and nonlocal‐aware optimization strategies provide promising routes for incorporating such interactions into the design process and jointly optimizing complex metasurface responses [28, 263]. Nevertheless, maintaining low crosstalk becomes increasingly difficult as the number of encoded channels and controlled optical degrees of freedom increases.

Metasurface devices combining broadband response with high efficiency face fundamental trade‐offs among transmission or reflection efficiency, operating bandwidth, numerical aperture, field of view, and functional complexity. High‐efficiency operation requires high transmission or reflection, complete phase coverage, and suppression of undesired scattering and diffraction channels, whereas broadband and achromatic operation additionally requires controlled spectral dispersion over multiple wavelengths. These requirements become increasingly difficult to satisfy simultaneously in multifunctional and high‐dimensional metasurfaces. For example, achromatic metalenses must simultaneously satisfy phase, group‐delay, and group‐delay‐dispersion requirements, leading to trade‐offs among device aperture, numerical aperture, operating bandwidth, and focusing efficiency [109, 200]. In high‐NA metasurfaces, discrete spatial sampling can further introduce unintended diffraction and efficiency loss; recent lattice‐based anti‐aliasing strategies have shown that the sampling lattice and spectral morphology can be jointly engineered to suppress such parasitic diffraction [32]. Multilayer architectures, dispersion engineering, nonlocal‐response design, and inverse optimization provide promising routes for expanding the accessible design space, although simultaneously achieving high efficiency, broad bandwidth, large numerical aperture, and multidimensional functionality remains challenging [28, 262].

Dynamic reconfigurability is essential for metasurfaces to evolve from static optical elements into intelligent photonic platforms. Liquid crystals, graphene, VO_2_, phase‐change materials, semiconductors, microelectromechanical systems, and electro‐optic materials have been widely explored for tunable and reconfigurable metasurfaces [265, 266]. Programmable circuits and time‐varying control schemes have further enabled dynamic beam control, tunable absorption, reconfigurable holography, intelligent reflecting surfaces, and spatiotemporal modulation [267]. Nevertheless, modulation depth, response speed, power consumption, operational stability, operating bandwidth, and fabrication compatibility remain challenging, particularly for high‐speed, high‐efficiency, large‐area metasurfaces operating in the visible and near‐infrared regimes.

Design methodology and fabrication are equally important. Metasurfaces for multidimensional light‐field manipulation involve numerous structural parameters and complex objective functions, making brute‐force parameter sweeps computationally inefficient. Machine learning, transfer learning, physics‐informed neural networks, topology optimization, and adjoint optimization can explore substantially larger design spaces and rapidly map desired optical responses to meta‐atom structures [264, 268]. Nevertheless, computationally optimized designs must satisfy practical fabrication constraints, including minimum feature size, inter‐element spacing, alignment tolerance, material uniformity, and large‐area process stability. Fabrication precision is particularly critical for multidimensional metasurfaces because deviations in feature size, height, sidewall profile, material properties, and layer‐to‐layer alignment can simultaneously perturb the designed phase, amplitude, polarization, and spectral responses. Such deviations can reduce optical efficiency, increase inter‐channel crosstalk, and degrade device‐to‐device reproducibility, making fabrication‐aware and tolerance‐robust design increasingly important for practical implementation [28, 262, 264].

Recent advances in scalable nanofabrication have significantly expanded the manufacturable size of metasurface devices. Deep‐ultraviolet projection lithography has progressed from centimeter‐scale wafer‐level metalenses to 80–100 mm‐aperture devices through multiple‐exposure and field‐stitching strategies, demonstrating the feasibility of large‐area and semiconductor‐compatible metasurface fabrication [198, 269, 270].

Beyond photolithography, replication‐based approaches, including wafer‐scale nanoimprint, roll‐to‐plate processing, and fully automated roll‐to‐roll nanoimprinting, have enabled scalable replication of centimeter‐scale visible metalenses and increasingly high‐throughput manufacturing [271, 272, 273]. Collectively, these developments indicate a transition from wafer‐scale batch fabrication toward low‐cost, high‐throughput, and continuous manufacturing, supporting the practical deployment and commercialization of meta‐optical devices [198, 269, 270, 271, 272, 273, 274]. Beyond advances in manufacturing scale and throughput, closed‐loop design‐fabrication‐characterization workflows that explicitly incorporate fabrication errors and experimental feedback will be essential for improving device reproducibility, robustness, and reliability.

The translation of recent metasurface advances into practical applications further requires system‐level integration. Although metasurfaces are progressing toward commercial deployment, scalable manufacturing and control, together with integration with light sources, detectors, waveguides, modulators, electronic drivers, and packaging platforms, remain major challenges [23, 24, 275]. In optical computing, metasurface‐based platforms increasingly require coordinated integration with photonic and electronic processing architectures, detectors, and readout systems to translate device‐level optical operations into scalable and intelligent information‐processing platforms [192, 193, 194]. In optical communication, metasurface‐enabled systems must be co‐designed with transmitters, receivers, and multiplexing and demultiplexing modules while suppressing inter‐channel crosstalk and preserving communication sensitivity [276]. In imaging and display, metasurfaces must be jointly optimized with image sensors, computational reconstruction algorithms, and near‐eye display architectures to achieve compact form factors without sacrificing field of view, color fidelity, or image quality [277, 278]. In optical tweezers and biophotonics, planar metalenses must ensure compatibility with liquid and microfluidic environments and provide stable optical trapping performance [279], while quantitative optical force measurements require reliable characterization of trap stiffness [280]. Such system‐level co‐design is expected to further expand metasurfaces from standalone optical components toward compact, intelligent, and application‐oriented optical platforms.

Overall, metasurface‐based multidimensional light‐field manipulation is evolving from single‐function wavefront engineering toward high‐dimensional optical information manipulation, dynamic reconfigurability, intelligent design, and increasingly integrated photonic systems. Future progress will require coordinated advances in meta‐atom design, material platforms, device architectures, intelligent optimization, scalable manufacturing, and system‐level integration. Only by achieving an appropriate balance among optical performance, dynamic reconfigurability, manufacturability, robustness, and system compatibility can metasurfaces for multidimensional light‐field manipulation progress from laboratory demonstrations toward practical deployment.

## 6 Summary

In summary, we have reviewed the technologies and applications of metasurface‐based multidimensional light‐field manipulation. Starting from generalized Snell's law, phase discontinuity, and meta‐atom scattering, we introduced the physical principles by which metasurfaces manipulate light fields and reviewed the basic mechanisms for controlling phase, amplitude, polarization, wavelength, and OAM. We then discussed representative strategies for joint phase‐amplitude, phase‐polarization, phase‐wavelength, phase‐OAM, spatial‐polarization, temporal‐phase, and higher‐dimensional control, highlighting the role of coordinated degrees of freedom in multichannel multiplexing, complex light‐field synthesis, and high‐dimensional information encoding. Representative applications in optical information processing and computing, imaging and display, optical communication and information transmission, and structured‐light generation and optical manipulation demonstrate the potential of multidimensional metasurfaces for improving device integration, information capacity, and functional complexity.

The essence of metasurface‐based multidimensional light‐field manipulation is to transform multiple physical degrees of freedom of light into designable, encodable, and multiplexable information channels. Compared with conventional bulk optical systems, metasurfaces provide more compact, flexible, and multifunctional light‐field manipulation at the subwavelength scale and on ultrathin platforms, offering a new technical route toward lightweight, highly integrated, and multichannel optical systems.

Although practical multidimensional metasurfaces still require breakthroughs in efficiency, bandwidth, dynamic reconfigurability, fabrication, and system‐level integration, their advantages in high‐dimensional optical information manipulation and multifunctional photonic integration are already clear. With continued progress in low‐loss materials, nanofabrication, intelligent inverse design, reconfigurable control, and on‐chip integration, metasurface‐based multidimensional light‐field manipulation is expected to play an increasingly important role in high‐capacity communication, computational imaging, optical computing, advanced displays, quantum information, and micro/nano‐manipulation, and to drive next‐generation optical systems toward ultrathin, multifunctional, integrated, and intelligent architectures.

## Author Contributions

**Heliang Yin**: writing – original draft, visualization, formal analysis, data curation, investigation. **Shuo Sun**: conceptualization, writing – review and editing, formal analysis, supervision, methodology. **Jiahao Zhang**: visualization, writing – original draft, formal analysis, validation. **Jianuo Hu**: validation, writing – original draft, formal analysis, software. **Yubin Qiao**: project administration, writing – review and editing, visualization, investigation. **Liang Chen**: writing – review and editing, project administration, resources, funding acquisition, validation, supervision. **Jin Li**: writing – review and editing, supervision, visualization, project administration.

## Conflicts of Interest

The authors declare no conflicts of interest.

## Data Availability

The data that support the findings of this study are available from the corresponding author upon reasonable request.
